# Redundant *SCARECROW* genes pattern distinct cell layers in roots and leaves of maize

**DOI:** 10.1242/dev.177543

**Published:** 2019-07-19

**Authors:** Thomas E. Hughes, Olga V. Sedelnikova, Hao Wu, Philip W. Becraft, Jane A. Langdale

**Affiliations:** 1 Department of Plant Sciences, University of Oxford, South Parks Road, Oxford OX1 3RB, UK; 2 Genetics, Development, and Cell Biology Department, Iowa State University, Ames, IA 50011, USA

**Keywords:** Radial-patterning, SCARECROW, Kranz, Mesophyll, Bundle-sheath, Maize

## Abstract

The highly efficient C_4_ photosynthetic pathway is facilitated by ‘Kranz’ leaf anatomy. In Kranz leaves, closely spaced veins are encircled by concentric layers of photosynthetic bundle sheath (inner) and mesophyll (outer) cells. Here, we demonstrate that, in the C_4_ monocot maize, Kranz patterning is regulated by redundant function of SCARECROW 1 (ZmSCR1) and a previously uncharacterized homeologue: ZmSCR1h. *ZmSCR1* and *ZmSCR1h* transcripts accumulate in ground meristem cells of developing leaf primordia and in *Zmscr1;Zmscr1h* mutant leaves, most veins are separated by one rather than two mesophyll cells; many veins have sclerenchyma above and/or below instead of mesophyll cells; and supernumerary bundle sheath cells develop. The mutant defects are unified by compromised mesophyll cell development. In addition to Kranz defects, *Zmscr1;Zmscr1h* mutants fail to form an organized endodermal layer in the root. Collectively, these data indicate that ZmSCR1 and ZmSCR1h redundantly regulate cell-type patterning in both the leaves and roots of maize. Leaf and root pathways are distinguished, however, by the cell layer in which they operate – mesophyll at a two-cell distance from leaf veins versus endodermis immediately adjacent to root vasculature.

## Abstract (precis)

**Summary:** Two duplicated maize *SCARECROW* genes control the development of the endodermis in roots and the mesophyll in leaves.

## INTRODUCTION

**Fig. 1.**
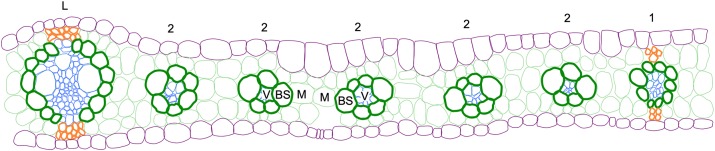
**Maize Kranz anatomy.** Cartoon depiction of a transverse maize leaf section. Distinct cell types are indicated by different colours; mesophyll (M), light green; bundle sheath (BS), dark green; vasculature (V), blue; epidermis, purple; sclerenchyma, orange. Lateral (L), rank 1 (1) and rank 2 (2) vein orders are indicated above each vein.

The C_4_ photosynthetic pathway, which is responsible for around 21% of global primary productivity, despite being found in only ∼3% of plant species (Ehleringer et al., 1997; Sage et al., 2011), is underpinned by a specialized leaf anatomy known as Kranz (the German word for wreath) (reviewed by Sedelnikova et al., 2018) (Fig. 1). Unlike in C_3_ plants, where photosynthesis occurs only in the mesophyll cells, the C_4_ pathway is separated between bundle sheath (BS) and mesophyll (M) cells, with the two cell types forming concentric wreaths around leaf veins (reviewed by Langdale, 2011). Efficient operation of the C_4_ cycle relies on an increased BS-to-M cell ratio relative to that seen in C_3_ plants, an increase that is achieved by altering vein density so that vascular bundles are often separated by only two M cells in a recurring vein-BS-M-M-BS-vein pattern across the leaf. In C_4_ monocots such as maize, this high vein density results from the formation of small ‘rank-2’ intermediate veins in between the lateral and rank-1 intermediate veins that are common to both C_3_ and C_4_ species (Esau, 1943; Russell and Evert, 1985; Sharman, 1942). Given the higher yields found in many C_4_ plants, there are ongoing attempts to engineer the C_4_ pathway into C_3_ crops (Hibberd et al., 2008; von Caemmerer et al., 2012; Wang et al., 2016); however, such attempts require a far better understanding of how vein spacing and leaf cell fate are regulated in C_4_ species.

Our understanding of the genetic components that regulate the development of Kranz anatomy is extremely limited, in part because traditional approaches to gene discovery, such as mutant screens, failed to reveal any regulators of vein spacing or BS/M cell fate (Langdale, 2011). More recent transcriptomic analyses identified candidate genes that are expressed in a manner consistent with roles in Kranz patterning (Fouracre et al., 2014; Wang et al., 2013), but in most cases gene function has yet to be validated. One candidate, the maize GRAS protein SCARECROW1 (ZmSCR1), has been shown to regulate aspects of Kranz patterning in that *Zmscr1* mutants have subtle alterations in vascular, BS and M development (Slewinski et al., 2012). In *Arabidopsis thaliana* (hereafter referred to as *Arabidopsis*) the *ZmSCR1* orthologue radially patterns cell-types in the root (Di Laurenzio et al., 1996; Wysocka-Diller et al., 2000); AtSCR prevents movement of AtSHORTROOT (AtSHR) beyond the cell layer adjacent to the vasculature, which ensures specification of endodermal cells in that layer (Cui et al., 2007). However, an organized endodermal cell layer is present in *Zmscr1* mutants (Slewinski et al., 2012), suggesting that gene function may have diverged between maize and *Arabidopsis*. Given that the root endodermis and the leaf BS are considered analogous cell types (Esau, 1943; Nelson, 2011), it is possible that an ancestral SCR patterning function was recruited in the leaf rather than the root in maize, but the subtle phenotype reported in leaves of *Zmscr1* mutants precludes an understanding of the precise role played during Kranz development.

Both gene and whole-genome duplication events are highly prevalent throughout the plant phylogeny (Adams and Wendel, 2005; Blanc and Wolfe, 2004) and if retained in the genome, duplicated genes are free to sub- or neo-functionalize (Moore and Purugganan, 2005; Ohno, 1970). Perhaps more commonly, however, gene duplicates function redundantly. Indeed, there are many examples illustrating the importance of genetic redundancy in plants, and without understanding phylogenetic context, loss-of-function data can be difficult to interpret (Strable et al., 2017; Yi et al., 2015). This is particularly important in maize, which, in addition to undergoing three ancient whole-genome duplication events common to monocots, has also undergone a more recent event not shared with its close relative *Sorghum bicolor* (Messing et al., 2004; Schnable et al., 2009; Swigonova et al., 2004). It is thus likely that *ZmSCR1* acts redundantly with a duplicate gene to pattern cell types in maize.

To better understand the role of ZmSCR1 in maize development, we first constructed a phylogeny of *SCR*-related genes, which revealed that *ZmSCR1* has a previously overlooked homeologue duplicate *ZmSCR1h*. When transposon-induced loss-of-function alleles of both genes were combined, double mutants exhibited leaf and root phenotypes that were not seen in segregating single mutant siblings. Cell-type specification was perturbed in both the leaf and root of *Zmscr1;Zmscr1h* double mutants, with endodermal defects observed in the root. Intriguingly, however, M rather than BS cell development was primarily perturbed in the leaf. We present a quantitative analysis of single and double *Zmscr1;Zmscr1h* mutant leaf phenotypes, plus expression data for both genes in developing wild-type maize leaf primordia. The results are discussed in the context of how SCR function has diversified in flowering plants.

## RESULTS

### *SCR* is duplicated in maize

**Fig. 2.**
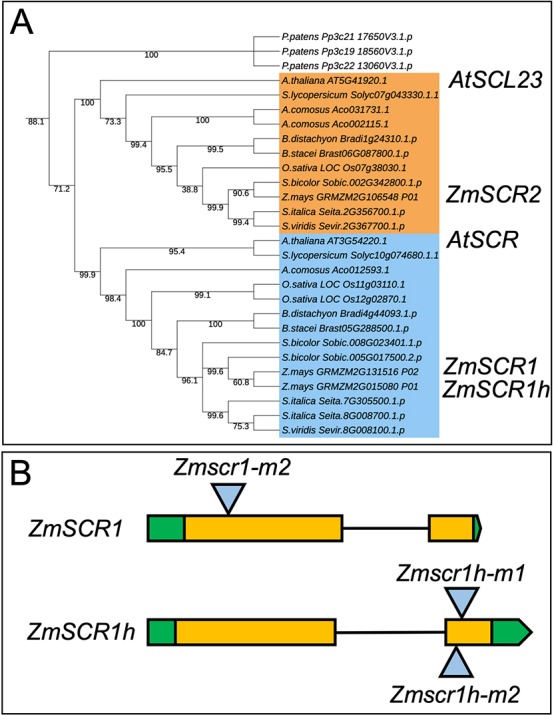
**Transposon insertions in maize *AtSCR* orthologues.** (A) Maximum likelihood phylogeny of SCR genes. Bootstrap values are indicated below branches. Light-blue shading indicates the *AtSCR* clade, light-orange shading indicates the *AtSCL23* clade. *Physcomitrella patens* sequences were included as an outgroup. (B) Cartoon depiction of *Mutator* transposon insertions in *ZmSCR1* and *ZmSCR1h*. All three alleles were in the W22 inbred background from the UniformMu project. UTRs (green), exons (orange), introns (black horizontal line) and transposon insertion site (blue triangle) are indicated.

To determine phylogenetic relationships between *SCR*-related genes in land plants, a maximum likelihood phylogeny was constructed. Fig. 2A shows that two clades of *SCR* genes are present in both eudicots and monocots, with the underlying duplication event inferred after the divergence of *Physcomitrella patens* and vascular plants. In *Arabidopsis*, the *SCR* clade contains a single gene (*AtSCR*), with the closest related homologue (*SCR-LIKE 23* – *AtSCL23*) in the sister clade. Apart from *Ananas comosus*, the sampled monocot genomes contain single-copy orthologues of *AtSCL23.* In contrast, *SCR* has independently duplicated in at least four monocot genomes (maize, *Sorghum bicolor*, *Setaria italica* and *Oryza sativa*), and the apparent single copy in *Setaria viridis* is likely an annotation error. The maize *SCR* duplicates reside on syntenic regions of chromosomes 4 (*ZmSCR1*) and 2 (*ZmSCR1h*), and have previously been annotated as likely homeologue gene pairs that arose through the recent maize whole-genome duplication (Schnable et al., 2011). Sequence comparisons reveal 85% amino acid identity between ZmSCR1 and ZmSCR1h, and both contain an N-terminal domain that prevents intercellular movement of the AtSCR protein (Gallagher and Benfey, 2009). These observations suggest that ZmSCR1 and ZmSCR1h act redundantly, and in a cell-autonomous manner.

### Transposon insertion alleles of *ZmSCR1* and *ZmSCR1h* cause loss of function

To test the hypothesis of functional redundancy, we first identified transposon insertion alleles for each gene. Two *Zmscr1* alleles (*-m1* and *-m2*) have been reported previously (Slewinski et al., 2012), and we identified two independent *Zmscr1h* alleles (*Zmscr1h-m1* and *-m2*) in the UniformMu transposon insertion collection (Fig. S1A) (McCarty et al., 2005). In both *Zmscr1h-m1* and *-m2*, a *Mutator* (*Mu*) element was predicted to be inserted in the second exon of the *ZmSCR1h*-coding sequence (Fig. 2B). Seed stocks for both alleles, plus *Zmscr1-m2*, which is in the same UniformMu W22 background, were obtained from the Maize Genetics Stock Centre (maizecoop.cropsci.uiuc.edu/). Single *Mu* insertions in the genes of interest are documented for the *Zmscr1-m2* and *Zmscr1h-m1* lines, whereas the *Zmscr1h-m2* line contains four additional *Mu* elements inserted at other loci (Fig. S1A). Insertion positions were confirmed by PCR amplification of genomic DNA, using primers in the transposon and in the adjacent genic region (Fig. S1B-D). In all cases, the size of the amplified product was consistent with the predicted insertion site. Primers flanking the *Mu* element enabled homozygous mutant individuals to be identified (Fig. S1B-D).

To confirm that the transposon insertion alleles compromised gene function, transcripts were amplified and sequenced, using RNA extracted from homozygous mutant leaf primordia as a starting template. Reverse transcriptase (RT)-PCR revealed that in all cases, the *Mu* element was present in the *ZmSCR1* or *ZmSCR1h* transcript, at the position predicted by the insertion site (Fig. S1E). As such, even if transcripts were translated, a non-functional protein would be produced.

### Loss-of-function *ZmSCR1h* mutants do not exhibit cell-type patterning defects

**Fig. 3.**
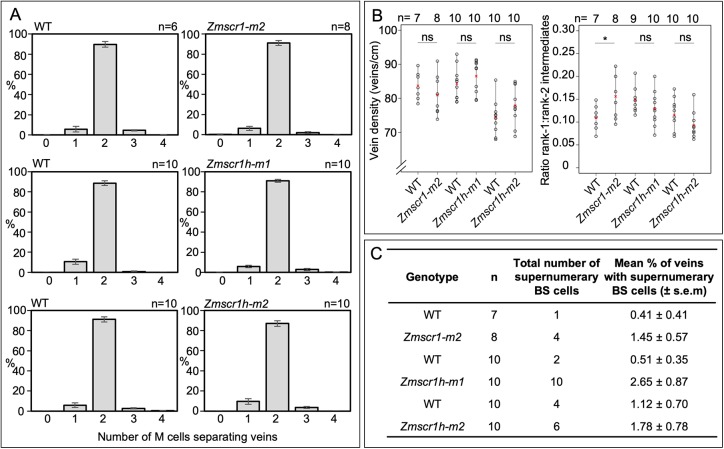
**Phenotype of *Zmscr1* and *Zmscr1h* single mutants.** (A) Quantification of mean percentage of M cells between vein pairs. In each case, the wild-type plot on the left represents a segregating sibling from the same family as the mutant presented in the corresponding plot on the right. Data are mean±s.e.m. (B) Quantification of vein density and the ratio of rank1 to rank 2 intermediate veins in leaf 5. In each case, data from segregating wild type (left) and corresponding mutant (right) are presented. Means are indicated by red crosses. Statistical significance between wild type and mutant was assessed using Student's *t*-test (two-tailed). ns, no significant difference; *P≤0.05. (C) Quantification of mean number of supernumerary BS cells in leaf 5. In each case, data from segregating wild type (top) and corresponding mutant (bottom) are presented. Raw phenotypic data are provided in Table S1.

To determine whether *Zmscr1h* mutants display similar defects in Kranz patterning to those reported in *Zmscr1* mutants (Slewinski et al., 2012), leaf traits were compared between *Zmscr1-m2*, *Zmscr1h-m1* and *Zmscr1h-m2* single mutants, and corresponding wild-type siblings segregating in each line. There was no qualitative difference between wild-type and either *Zmscr1* or *Zmscr1h* single mutants in overall plant growth (Fig. S2A-D), or in general Kranz patterning (Fig. S2E-H). Quantification of the number of M cells between veins (Fig. 3A), vein density across the leaf (Fig. 3B), and the ratio of rank-1:rank-2 intermediate veins (Fig. 3B), failed to confirm previous reports of altered vein density and interveinal M cell number in *Zmscr1-m2* mutants, which may reflect differences in sampling strategies and growth conditions between the two studies (Slewinski et al., 2012). However, *Zmscr1-m2* mutants did exhibit a small but significant increase in the ratio of rank-1:rank-2 intermediate veins (Fig. 3B). No significant difference was observed between wild-type and either *Zmscr1h* mutant allele for any of the measured traits. Previous reports of supernumerary BS cells in *Zmscr1-m2* mutants (Slewinski et al., 2012) were confirmed, and the trait was also seen in *Zmscr1h-m1* and *Zmscr1h-m2* single mutants (Fig. 3C). However, very few instances were observed above background levels in segregating wild-type siblings and over 97% of veins in mutant leaves were surrounded by a normal BS cell layer (Fig. 3C). As such, it can be concluded that mutations in *ZmSCR1* or *ZmSCR1h* cause only minor perturbations to Kranz patterning mechanisms.

In the absence of any major defects in *Zmscr1h* mutant leaves, and given that root development is perturbed in *Arabidopsis* mutants, we also examined cell-type patterning in single mutant roots. In *Atscr* mutants, instead of distinct layers of endodermis and cortex differentiating around the vasculature, a single cell layer with characteristics of both cell types forms (Di Laurenzio et al., 1996). Notably, qualitative histological analysis of *Zmscr1-m2*, *Zmscr1h-m1* and *Zmscrh1-m2* root sections revealed normal development, with a clear endodermal boundary between the vasculature and the multiple cortical layers that are characteristic in maize (Fig. S2I-L). This observation suggests either that *ZmSCR1* and *ZmSCR1h* act redundantly to form an endodermal layer in the maize root, or that the role of the SCR pathway in patterning endodermis has diverged between *Arabidopsis* and maize.

### *Zmscr1;Zmscr1h* double mutants exhibit stunted growth

To distinguish hypotheses of redundant versus divergent function of ZmSCR1 and ZmSCR1h, double mutant lines were generated with *Zmscr1-m2* and the two independent *Zmscr1h* alleles. In F2 populations, 6/107 and 5/78 individuals were genotyped as *Zmscr1-m2;Zmscr1h-m1* and *Zmscr1-m2;Zmscr1h-m2*, respectively, which matched the expected segregation ratio (χ^2^ *P*>0.05 in both cases). Although *Zmscr1;Zmscr1h* double mutants sometimes formed tassels when grown in the greenhouse, they rarely produced ears, and plants were never successfully self-pollinated. As such, phenotypic analysis was initially carried out using F2 populations segregating 1 in 16 for the homozygous double mutants. More-detailed characterization was undertaken with F3 progeny of self-pollinated F2 *Zmscr1-m2/+;Zmscr1h-m1* plants, which segregated 1 in 4 for the *Zmscr1-m2;Zmscr1h-m1* homozygous double mutants. In all experiments, comparisons were made to wild-type plants segregating in the same population.

**Fig. 4.**
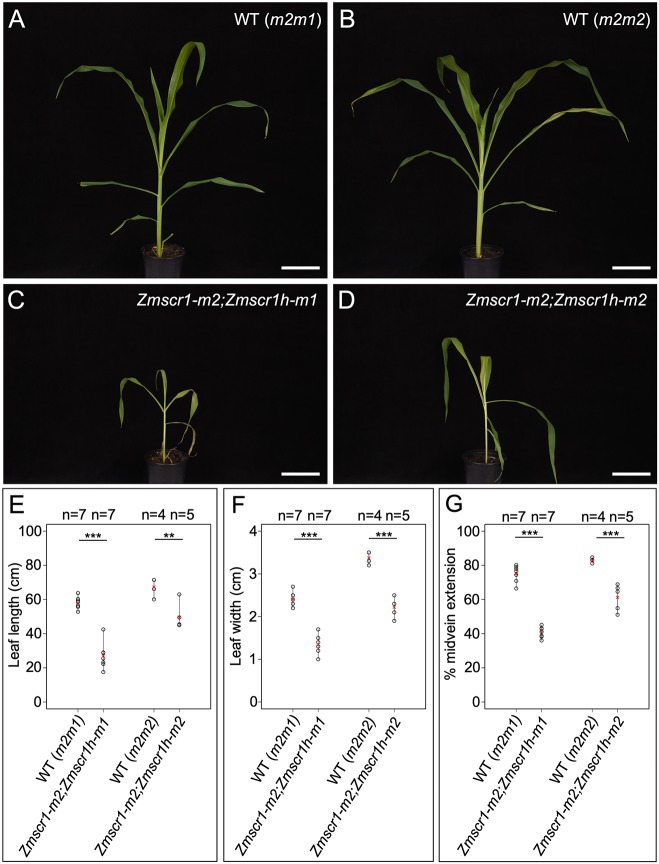
**Growth is stunted in *Zmscr1;Zmscr1h* mutants.** (A-D) 32-day-old segregating wild type (A,B) and *Zmscr1;Zmscr1h* double mutant siblings (C,D). Scale bars: 10 cm. (E-G) Quantification of leaf blade length from ligule to tip (E); leaf width at the midpoint along the proximal/distal axis (F); and the percentage extension of the midvein (G). Quantification was undertaken either 31 (*m2m1*) or 32 (*m2m2*) days after planting. Means are indicated by red crosses. Statistical significance between wild type and mutants was assessed using Student's *t*-tests (two-tailed): **P≤0.01; ***P≤0.001. Raw phenotypic data are provided in Table S1.

Unlike segregating single mutants, double mutants exhibited slower growth and reduced plant size (Fig. 4), similar to that reported for *Atscr* mutants (Di Laurenzio et al., 1996). Furthermore, *Zmscr1-m2;Zmscr1h-m1* and *Zmscr1-m2;Zmscr1h-m2* mutant leaves were, on average, 46% and 74% the length of corresponding wild-type leaves, respectively, with leaf width also proportionally reduced (Fig. 4E,F). Despite the plants being smaller, they were not markedly developmentally delayed, because the emergent leaf in *Zmscr1-m2;Zmscr1-m1* double mutants was only around one plastochron (the time interval between initiation of leaves at the shoot apex) behind that of wild type after 31 days of growth (Fig. S3A). Strikingly, however, emerging leaves in both double mutants were droopy (Fig. 4C,D). This phenotype was associated with a reduction in midrib length such that it extended, on average, 41% (*Zmscr1-m2;Zmscr1h-m1*) and 61% (*Zmscr1-m2;Zmscr1h-m2*) of the total leaf length compared with 75% and 83% in leaves of wild-type siblings (Fig. 4G). Defective growth was more severe in field-grown plants, suggesting an environmental influence.

### ZmSCR1 and ZmSCR1h redundantly regulate formation of the endodermis in maize roots

**Fig. 5.**
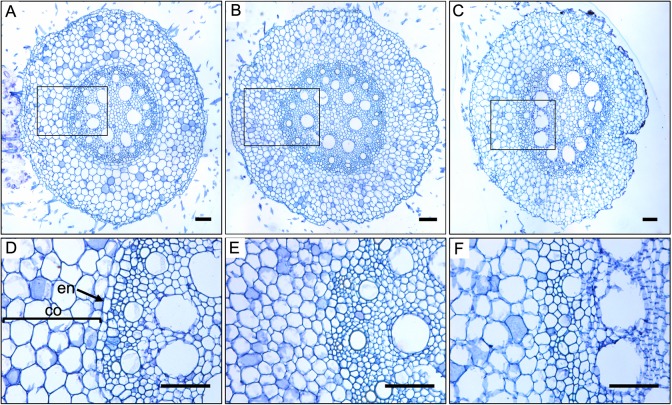
**ZmSCR1 and ZmSCR1h regulate endodermis formation during root development.** Representative transverse sections of roots from wild-type plants segregating in the *Zmscr1-m2;Zmscr1h-m1* background (A,D) plus *Zmscr1-m2;Zmscr1h-m1* (B,E) and *Zmscr1-m2;Zmscr1h-m2* (C,F) double mutants. Sections were taken from the maturation zone of either primary roots 4 days after germination (A,B,D,E) or seminal roots 35 days after germination (C,F). D-F are enlargements of the areas indicated by the rectangles in the corresponding whole-root image in A-C, respectively. Cortex (co) and endodermis (en) are indicated. Scale bars: 100 µm.

Given the environmental influence on the stunted growth phenotype of *Zmscr1;Zmscr1h* double mutants, and the well-characterized phenotype of *Atscr* mutants, we hypothesized that loss of *ZmSCR1* and *ZmSCR1h* function disrupted differentiation of the root endodermis. Transverse sections from the maturation zone of *Zmscr1-m2;Zmscr1h-m1* primary roots were therefore examined. Fig. 5 shows that overall size and structure of the root is normal, with apparently typical differentiation of cortex cells (Fig. 5A,B). However, there is no clear boundary between the vasculature and cortex (Fig. 5E). This pattern was also apparent in the seminal roots of *Zmscr1-m2;Zmscr1h-m2* mutants (Fig. 5C,F). These results demonstrate that ZmSCR1 and ZmSCR1h redundantly regulate formation of an organized endodermal layer in maize roots, possibly in an analogous manner to AtSCR in *Arabidopsis*.

### Bundle sheath cell specification is not perturbed in *Zmscr1;Zmscr1h* mutant leaves

**Fig. 6.**
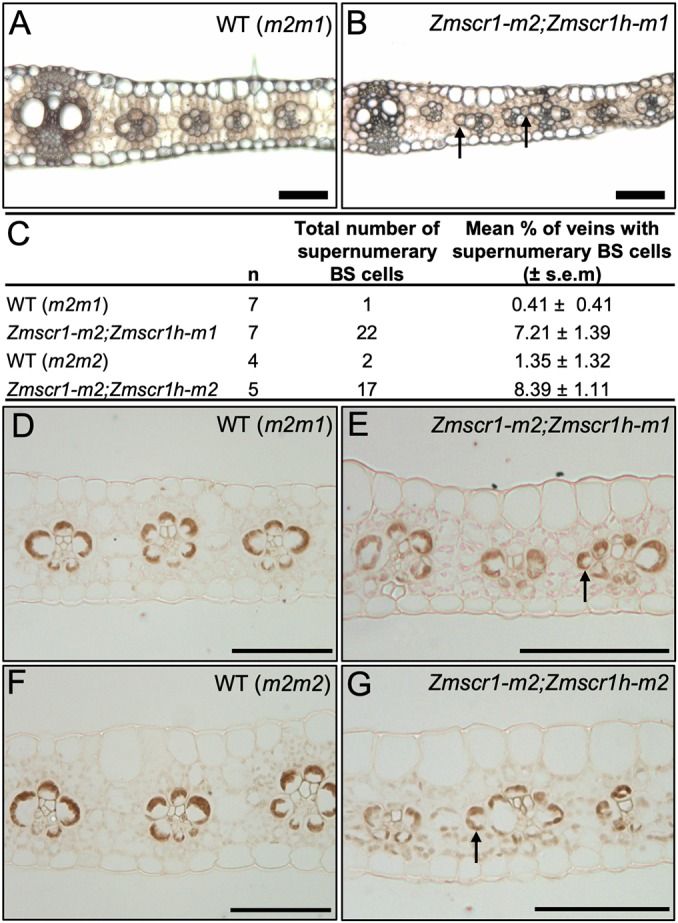
***Zmscr1;Zmscr1h* mutants form supernumerary BS cells.** (A,B) Representative fresh-cut transverse sections of fully expanded leaf 5 from wild-type (*m2m1* segregant) (A) and *Zmscr1-m2;Zmscr1h-m1* mutant (B) plants. (C) Quantification of the frequency of extra BS cells in different mutant backgrounds. Wild-type (*m2m1*) data are also presented in Fig. 3C. Raw phenotypic data are provided in Table S1. (D-G) Immunolocalization of ZmNADP-ME in BS cell chloroplasts of wild-type [*m2m1* segregant (D); *m2m2* segregant (F)], *Zmscr1-m2;Zmscr1h-m1* (E) and *Zmscr1-m2;Zmscr1h-m2* (G) leaves. Arrows in B,E,G indicate extra BS cells that are not in contact with the vasculature. Scale bars: 100 µm.

The original thesis that the SCR pathway may regulate Kranz patterning in the maize leaf was predicated on the long-held view that the root endodermis and leaf BS are analogous cell types, and that radial patterning mechanisms may be conserved in root and leaf (Slewinski, 2013). Given the disruption of an organized endodermal layer in *Zmscr1;Zmscr1h* double mutants (Fig. 5), if leaf and root pathways are conserved, BS cell formation should be severely perturbed in leaves. To test whether this is the case, the position and number of BS cells was quantified across double mutant leaves (Fig. 6A,B, Fig. 3C). Crucially, in cleared transverse sections, where BS cells can be clearly distinguished from M cells on the basis of cell shape and cell wall architecture, all veins formed a BS cell layer (Fig. 6A,B). As such, whereas the SCR pathway is necessary for the formation of an organized endodermal layer in the root, it is not required for the development of a BS cell layer around leaf veins.

Although double mutants did not produce deficiencies in the layer of BS cells around each vein, instances of supernumerary BS-like cells outside of the normal layer were observed (Fig. 6A,B). Quantification of this phenotype revealed significantly higher frequencies in both *Zmscr1;Zmscr1h* double mutants than in single mutants (Fig. 6C). However, even in the double mutants, over 90% of leaf veins had normal BS cell layers with no supernumerary cells. To resolve whether both supernumerary and normally positioned BS cells in *Zmscr1;Zmscr1h* mutants were functionally equivalent to wild-type BS cells, immunolocalization using an antibody against NADP-malic enzyme (NADP-ME) was carried out. NADP-ME accumulated specifically in BS chloroplasts of wild-type maize leaves (Fig. 6D,E) and was detected in supernumerary BS-like cells in double mutant leaves (Fig. 6F,G). Interestingly, there are examples of normally positioned BS cells that do not accumulate NADP-ME at high levels in *Zmscr1;Zmscr1h* leaves (Fig. 6F,G), implying a possible defect in photosynthetic differentiation. This would fit with the previous finding that there are BS cells in *Zmscr1* mutants that fail to accumulate starch (Slewinski et al., 2012). Given the BS identity, cell position, and low frequency occurrence of these supernumerary cells, it is most likely that they are formed by late divisions of cells that are already differentiated as BS in the layer around the vein; however, a direct role for ZmSCR1 and ZmSCR1h in BS cell patterning cannot be eliminated.

### *ZmSCR1* and *ZmSCR1h* transcripts accumulate in ground meristem cells of leaf primordia

**Fig. 7.**
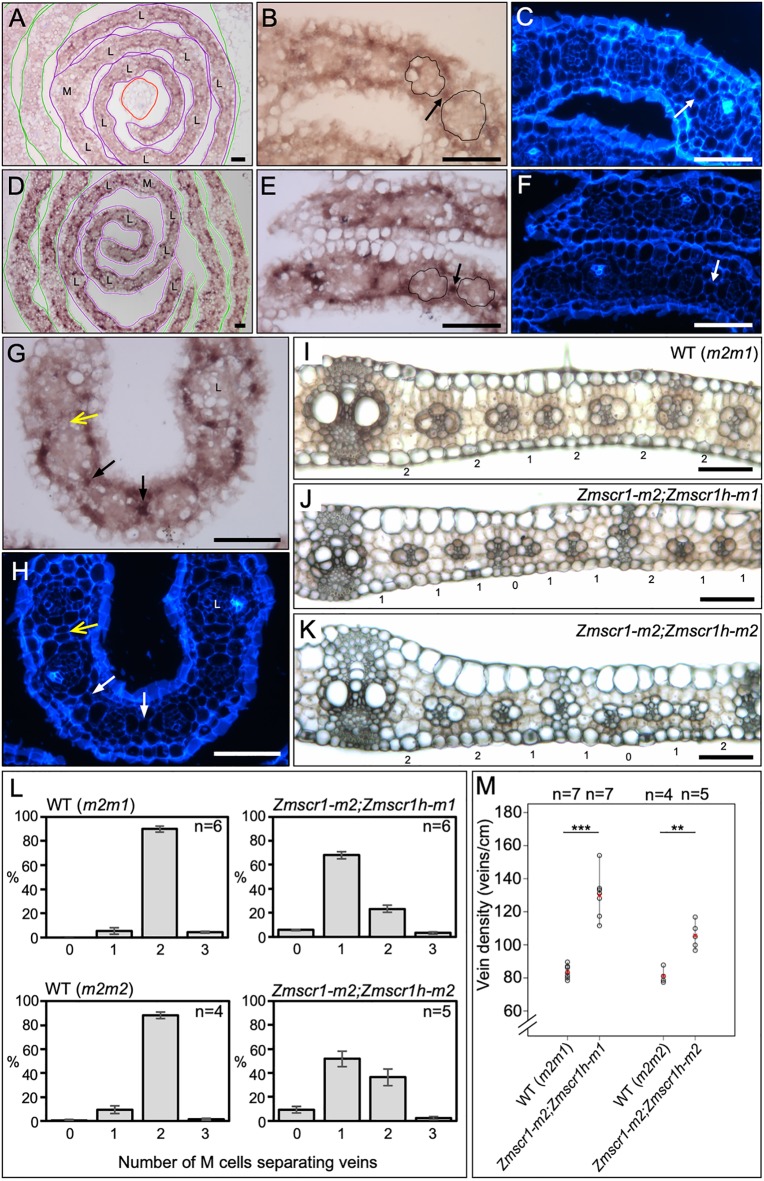
**ZmSCR1 and ZmSCR1h regulate divisions of ground meristem cells.** (A-H) *In situ* hybridization of *ZmSCR1* (A-C) and *ZmSCR1h* (D-H) in developing leaf primordia. Plastochron (P) numbers are indicated by coloured outlines: red, P3; purple, P4; green, P5 (A,D). Higher-magnification P4 cross-sections were imaged under both bright-field (B,E,G) and UV (C,F,H) illumination to show hybridization signal and calcofluor staining of cell walls, respectively. Black and white arrows indicate single ground meristem cells separating developing veins, yellow arrows indicate two ground meristem cells separating developing veins. The midvein (M) and lateral veins (L) are indicated on P4 primordia. Scale bars: 50 µm. (I-K) Representative fresh transverse sections of wild-type (*m2m1* segregant) (I), *Zmscr1-m2;Zmscr1h-m1* (J) and *Zmscr1-m2;Zmscr1h-m2* (K) fully expanded leaf 5. Scale bars: 100 µm. Numbers below each section are the number of M cells separating the vascular bundles. Note that I is the same image as Fig. 6A. (L) Quantification of the mean percentage of vascular bundles separated by 0-3 M cells in wild-type and *Zmscr1;Zmscr1h* fully expanded leaf 5. Data are mean±s.e.m. (M) Quantification of vein density in *Zmscr1;Zmscr1h* double mutants and corresponding wild-type segregants. Means are indicated by red crosses. Statistical significance between wild type and mutants was assessed using Student's *t*-tests (two-tailed): **P≤0.01; ***P≤0.001. The wild types (*m2m1*) in L and M are also presented in Fig. 3A,B. Raw phenotypic data are provided in Table S1.

As the protein sequences of both ZmSCR1 and ZmSCR1h predict cell-autonomous function, we sought to determine where the genes might act by determining spatial and temporal patterns of transcript accumulation during early leaf development. To this end, *in situ* hybridization was undertaken with developing wild-type leaf primordia. Fragments in the first exon (*ZmSCR1*) or the 3′UTR (*ZmSCR1h*), which were predicted to be gene specific (Fig. S4A-D), were used to distinguish *ZmSCR1* and *ZmSCR1h*. A sense version of the *ZmSCR1h* probe was used as a negative control, (Fig. S4E-H). In plastochron (P)4 and P5 leaf primordia, transcripts of both genes were detected in the layer of ground meristem cells that surrounds developing veins, but were not detected in dividing procambial centres or in procambial-derived BS precursor cells (Fig. 7A-H). Notably, strong signal was observed in the single ground meristem cells that are present between developing veins at P4 (Fig. 7B,C,E-H). These cells divide to form the two M cells present in the recurring vein-BS-M-M-BS-vein units that characterize Kranz anatomy in maize. *ZmSCR1h* transcripts could not be detected in the regions of primordia where two interveinal cells were already present (Fig. 7G,H). These results seemingly contrast with a previous finding that *ZmSCR1* (previously referred to as *ZmSCR*) transcripts accumulate in developing veins of leaf primordia (Lim et al., 2005). Importantly, the Lim et al. (2005) study was undertaken before the maize genome sequence was available and, as such, the specificity of the probe sequence used is unclear. Furthermore, leaf sections were not counterstained, which makes it hard to resolve the different cell types. Our finding, that gene-specific probes from distinct regions of *ZmSCR1* and *ZmSCR1h* transcripts reveal very similar signal accumulation patterns in ground meristem cells encircling developing vascular bundles, led us to hypothesize that ZmSCR1 and ZmSCR1h pattern the cell layer encircling the BS during the development of Kranz anatomy.

### Impaired mesophyll cell divisions are associated with higher vein density in *Zmscr1;Zmscr1h* mutant leaves

Consistent with transcript accumulation in ground meristem cells between developing veins of wild-type P4 leaf primordia, there was a marked increase in the number of veins separated by only a single M cell in mature fully expanded leaves of *Zmscr1;Zmscr1h* mutants (Fig. 7I-L). In segregating wild-type backgrounds (and in single mutants; Fig. 3A), around 90% of all veins were separated by two M cells, whereas in both *Zmscr1-m2;Zmscr1h-*m1 and *Zmscr1-m2;Zmscr1h-m2* mutants, most veins were separated by one M cell (68% and 52% of veins surveyed, respectively) (Fig. 7L). Veins with no M cells in between were observed at 5-10% frequency in double mutants. This low-frequency phenotype could result from the failure to specify a M precursor cell (and any subsequent divisions) or from ectopic BS cell divisions displacing M cells, either of which would effectively lead to vein anastomoses. The more penetrant phenotype suggests that ZmSCR1 and ZmSCR1h act redundantly to promote the cell division that generates two M cells in the characteristic vein-BS-M-M-BS-vein unit of Kranz.

To determine whether the decrease in M cell number between veins resulted in higher vein density across the leaf, or whether a compensatory reduction in vein number was manifest, vein number and leaf width were quantified in wild-type and double mutant leaves. Fig. 7M shows that total vein density is significantly increased in *Zmscr1;Zmscr1h* double mutants. As well as reflecting fewer M cell divisions, this phenotype could also reflect the reduced width of *Zmscr1;Zmscr1h* mutant leaves (Fig. 4F), as both leaf width and cell size influence vein density in monocots. Either way, the increased vein density in *Zmscr1;Zmscr1h* mutants does not reflect an increase in the total number of veins formed across the width of the leaf, as there are fewer total veins formed in the narrower leaves of *Zmscr1;Zmscr1h* mutants (Fig. S2B). Rather than implying a direct role for ZmSCR1 and ZmSCR1h in the initiation and development of leaf veins, it is most parsimonious to suggest that the reduced width of *Zmscr1;Zmscr1h* leaves offsets the increased vein density to the extent that total vein number is still lower than wild type.

### The relative proportions of intermediate vein ranks is altered in *Zmscr1;Zmscr1h* mutants

**Fig. 8.**
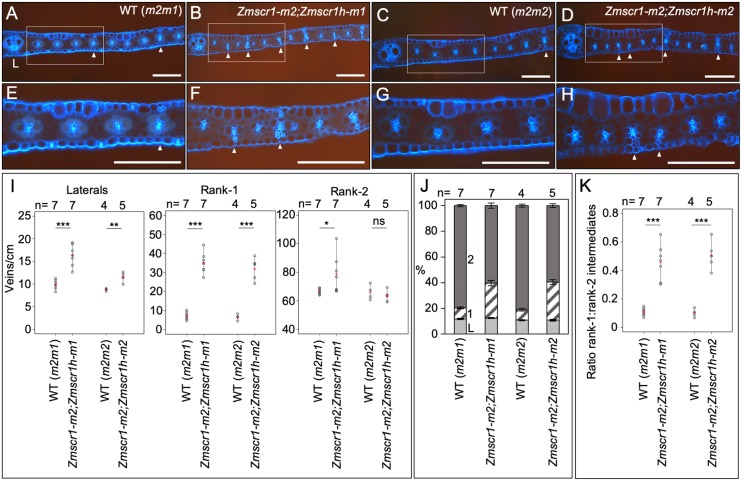
***Zmscr1;Zmscr1h* mutants form altered ratios of rank 1 to rank 2 intermediate veins.** (A-H) Representative fresh-cut transverse sections of fully expanded leaf 5 from wild-type [*m2m1* segregant (A,E); *m2m2* segregant (C,G)] and mutant [*Zmscr1-m2;Zmscr1h-m1* (B,F); *Zmscr1-m2;Zmscr1h-m2* (D,H)] leaves imaged under UV illumination to show fluorescence associated with lignin. Boxes in A-D indicate areas that are enlarged in E-H, respectively. White arrowheads indicate rank 1 intermediate veins with accompanying lignified sclerenchyma. L indicates a lateral vein. Scale bars: 100 µm. (I) Quantification of the number of different vein types per cm of leaf width. (J) The relative proportions of lateral veins (L, light grey), rank 1 intermediate veins (1, striped) and rank 2 intermediate veins (2, dark grey). Data are mean±s.e.m. (K) Quantification of the ratio of rank 1 to rank 2 intermediate veins. In I and K, means are indicated by red crosses; statistical significance between wild type and mutants was assessed using Student's *t*-test (two-tailed). ns, no significant difference; *P≤0.05, **P≤0.01, ***P≤0.001. Wild-type (*m2m1*) data in K are also presented in Fig. 3B. Raw phenotypic data are provided in Table S1.

Although a role for *ZmSCR1* and *ZmSCR1h* in regulating the number of leaf veins was deemed unlikely, quantification of vein numbers and ranks in *Zmscr1;Zmscr1h* mutants revealed a striking shift in the proportion of each vein type that developed. *Zmscr1;Zmscr1h* mutants formed lignified sclerenchyma ad- and abaxially to the vein at far greater frequency than in wild type (Fig. 8). In both C_3_ and C_4_ monocots, sclerenchyma is associated with both lateral and rank 1 intermediate veins. Rank 2 intermediate veins that do not form lignified sclerenchyma only form in C_4_ leaves with Kranz anatomy. In a typical wild-type maize leaf, two lateral veins are separated by between one and three evenly spaced rank 1 intermediate veins. These are in turn separated by multiple rank 2 intermediate veins, leading to a ratio of rank 1 to rank 2 veins of less than 0.2 (indicating on average more than five rank 2 veins to every rank 1 vein) (Fig. 8A,C,E,G,K). In *Zmscr1;Zmscr1h* leaves, more veins with sclerenchyma were observed than in wild type (Fig. 8B,D,F,H). Quantification of the numbers of each vein type in *Zmscr1;Zmscr1h* leaves revealed a small increase in the number of lateral veins forming per cm of leaf width (Fig. 8I), and there were slightly more rank 2 intermediate veins in the *Zmscr1-m2;Zmscr1h-m1* mutant but not in *Zmscr1-m2;Zmscr1h-m2* (Fig. 8I). Strikingly, the number of rank 1 intermediate veins that form per cm of leaf width is increased around fourfold in both *Zmscr1;Zmscr1h* double mutants (Fig. 8I). When expressed as a proportion of the total number of veins, which eliminates the influence of the increased vein density in *Zmscr1;Zmscr1h* mutants, it is clear that the percentage of lateral veins remains unaltered; however, there is a significant increase in the percentage of rank 1 intermediate veins and a concomitant reduction in the percentage of rank 2 intermediate veins (Fig. 8J). This results in a consistent rank 1:rank 2 ratio of around 0.5 (indicating only two rank 2 veins to every rank 1 vein) is observed in *Zmscr1;Zmscr1h* double mutants (Fig. 8K), a ratio that is much greater than that seen in single *Zmscr1-m2* mutants (Fig. 3B). Notably, the extra rank 1 intermediates are not evenly spaced as in wild type, but instead can be immediately adjacent to other rank 1 intermediate veins (Fig. 8D), and sclerenchyma is preferentially positioned on the abaxial side of the vein. Given that *ZmSCR1* and *ZmSCR1h* transcripts accumulate in ground meristem cells both ab- and adaxially to developing veins (Fig. 7A-H), the observed shift in vein ranks in double mutants might suggest that, in wild-type leaves, ZmSCR1/ZmSCR1h promote cell divisions and/or specification of M cells in these regions, and in so doing suppress sclerenchyma formation.

## DISCUSSION

Pattern formation is a fundamental process in both plant and animal development. In plants, radial patterning around the vasculature is of particular importance, and in the root is regulated by the SHR/SCR pathway (Cui et al., 2007). Here, we have shown that maize encodes two SCR genes that are equally orthologous to *AtSCR* (Fig. 2), and that *Zmscr1;Zmscr1h* double mutants exhibit a perturbed growth phenotype (Fig. 4) that closely resembles that seen in *Atscr* mutants (Di Laurenzio et al., 1996). Endodermis differentiation is disrupted in *Zmscr1;Zmscr1h* roots, with no clear organized cell layer between the cortex and vasculature (Fig. 5), which is consistent with the reported localization of *ZmSCR1* transcripts in the developing endodermis (Lim et al., 2005). However, this phenotype appears somewhat distinct from *Atscr* mutants in which a single organized ground-tissue layer with features of both the endodermis and cortex surrounds the vasculature (Di Laurenzio et al., 1996). In leaves, *ZmSCR1* and *ZmSCR1h* transcripts accumulate preferentially in ground meristem cells that will divide and differentiate into M cells (Fig. 7A-H), and fewer M cells are found in mature leaves of *Zmscr1;Zmscr1h* mutants than in wild type (Fig. 7I-L). All of the phenotypic perturbations observed in double mutants are either absent or significantly less frequent in single mutants (Fig. 3), indicating that ZmSCR1 and ZmSCR1h function redundantly. Collectively, these results demonstrate that the SCR radial patterning mechanism operates in both roots and leaves of maize.

The canonical SHR/SCR pathway, in which AtSCR prevents AtSHR movement more than one cell layer away from the root vasculature, was characterized in the context of roots with a single layer of both endodermis and cortex (Cui et al., 2007). However, maize and other monocots form multiple cortex layers (Clark and Harris, 1981; Dolan et al., 1993; Hochholdinger et al., 2018; Wu et al., 2014). It has been proposed that the number of cortex cell-layers in monocots is regulated by the extent to which SHR moves away from the root vasculature (Henry et al., 2017; Wu et al., 2014). Although SHR movement has not been confirmed in maize, there is no obvious change in the number of cortex cell layers formed in *Zmscr1;Zmscr1h* mutants, despite the endodermal layer being disrupted (Fig. 5). This can be explained in one of three ways: (1) SHR is not involved in regulating cortical cell layers in maize; (2) SCR does not restrict SHR movement in maize roots, a suggestion supported by the finding that movement of monocot SHR proteins was not constrained *in planta* by interaction with AtSCR (Wu et al., 2014); or (3) SHR is necessary but not sufficient to induce the formation of extra cell layers, consistent with the finding that *Atscr* mutants form only one ground-tissue layer despite SHR movement being unconstrained (Di Laurenzio et al., 1996). Crucially, all of these alternatives indicate that the canonical SHR/SCR pathway is modified in roots that develop multiple layers of cortex.

The root endodermis and leaf BS are considered analogous (Esau, 1943; Nelson, 2011), and as such it has been suggested that the differentiation of both cell types is regulated by the same genetic mechanism (Slewinski, 2013). However, in *Zmscr1;Zmscr1h* mutants, endodermis differentiation is disrupted such that no organized cell layer is apparent between the cortex and vasculature, whereas all leaf veins have a ring of surrounding BS cells (Fig. 6). In some cases, supernumerary BS cells that resemble those seen in the maize *tangled1* mutant (Jankovsky et al., 2001), are also observed around leaf veins (Fig. 6). These supernumerary cells might imply a role for ZmSCR1 and ZmSCR1h in BS patterning; however, in the *tangled1* mutant these supernumerary cells result from abnormal late divisions caused by perturbations in cell-division planes throughout the leaf (Jankovsky et al., 2001), suggesting that similar compensatory cell divisions may occur in *Zmscr1;Zmscr1h* mutants in response to the observed aberrant divisions of ground meristem cells. Although a role for ZmSCR1 and ZmSCR1h in specifying BS cell fate remains formally possible, given that there is no evidence of preferential BS expression either early in leaf development (Fig. 7A-H) or in mature leaves (Chang et al., 2012; Denton et al., 2017; Li et al., 2010; Tausta et al., 2014), and that amino acid sequences predict both proteins are immobile (Gallagher and Benfey, 2009), a role in M patterning is more likely. Perturbations in *Zmscr1;Zmscr1h* leaves validate this suggestion in that most veins are separated by only one M cell, indicating impaired division of the single ground meristem cell that is marked by high *ZmSCR1* and *ZmSCR1h* transcript accumulation (Fig. 7). In addition, sclerenchyma forms ab- and adaxially to veins where M cells would normally develop (Fig. 8), suggesting that ZmSCR1 and ZmSCR1h inhibit the longitudinal divisions that give rise to sclerenchyma (Bosabalidis et al., 1994; Esau, 1943) and/or promote M cell differentiation. Taken together, these results refute the hypothesis that the endodermis and BS are patterned by the same mechanism, and instead suggest that SCR functions to promote the development of endodermal cells in the root and M cells in the leaf.

The most-consistent mutant phenotype in *Zmscr1;Zmscr1h* leaves is the increased number of veins separated by only one M cell, which accounts for 68% (*Zmscr1-m2;Zmscr1h-m1*) or 52% (*Zmscr1-m2;Zmscr1h-m2*) of veins in double mutants compared with less than 10% in wild type (Fig. 7L). However, penetrance is clearly not complete as 23% and 36% of veins are still separated by two M cells (Fig. 7L). Notably, this contrasts with complete penetrance of the endodermal defects in *Zmscr1;Zmscr1h* mutant roots (Fig. 5). We hypothesize that this difference reflects single versus multiple clonal origins of endodermal and M cells. At least in *Arabidopsis*, all endodermal cells arise from divisions of initials that are distinct from those that form the vasculature (Dolan et al., 1993). By contrast, although the central ground meristem layer gives rise to all leaf veins and BS cells plus M cells in that layer (Langdale et al., 1989), once procambium has been specified, the two origins of central M cells can be distinguished (Jankovsky et al., 2001). Lineage analyses found that 67% of sectors induced after procambium initiation comprised a complete ring of BS cells but no M cells, whereas 33% of sectors consisted of a few cells in the BS layer plus one or more adjacent M cells (Jankovsky et al., 2001). As such, two-thirds of M cells in the central leaf layer originate from ground tissue that is clonally distinct from the BS, whereas one-third are clonally related to adjacent BS cells. These proportions are consistent with the percentages of veins separated by one (∼60%) versus two (∼40%) M cells in *Zmscr1;Zmscr1h* mutants, and thus suggest that the division and differentiation of M cells originating from the same precursor cell as the vascular bundle is regulated by a SCR-independent mechanism. This implies that the transition from ground meristem to M cell is regulated by at least two distinct mechanisms within the maize leaf.

It has been hypothesized that SHR/SCR-mediated patterning of cell types in the leaf is specific to C_4_ Kranz anatomy (Slewinski, 2013). Current evidence is supportive of Kranz-specific SHR/SCR roles in that *Atscr* mutant leaves exhibit only a slight enlargement of BS cell size (Cui et al., 2014) and constitutive expression of *ZmSCR1* in rice failed to disrupt any aspect of leaf development (Wang et al., 2017). These observations suggest that SCR is neither necessary nor sufficient to regulate the spatial arrangement of cell types in the inner leaf layers of C_3_ plants. Transcripts of one of the two rice SCR orthologues localize to cells that give rise to stomata (Kamiya et al., 2003), and both *Osscr* mutants and *ZmSHR1* overexpression rice lines induce changes in stomatal cell patterning (Schuler et al., 2018; Wu et al., 2019), suggesting that the SHR/SCR pathway may regulate cell-type specification in the epidermis rather than in the inner leaf layers of rice. However, this suggestion needs further investigation given that our data reveal a role for SCR in M-cell development and that M cells are a common feature of both C_3_ and C_4_ leaves. Based on current evidence, we conclude that the SHR/SCR pathway represents a flexible regulatory module that has been co-opted to pattern cell types in a range of developmental contexts in both roots and shoots of flowering plants.

## MATERIALS AND METHODS

### Plant stocks and growth conditions

UniformMu seed stocks harbouring *Mutator* insertions in either *ZmSCR1* (GRMZM2G131516) or *ZmSCR1h* (GRMZM2G015080) were acquired from the maize genetics COOP stock centre (maizecoop.cropsci.uiuc.edu) (Fig. S1A). Plants were grown in the field at Iowa State University, USA, and individuals harbouring the *Zmscr1-m2* allele were outcrossed to those harbouring either the *Zmscr1h-m1* or *-m2* allele. F1 plants heterozygous for both insertions were self-pollinated to yield F2 populations segregating 1/16 for both segregating wild-type and *Zmscr1;Zmscr1h* double mutants. Self-pollinations of *Zmscr1-m2/+;Zmscr1h-m1* F2 plants resulted in F3 families where *Zmscr1-m2;Zmscr1h-m1* homozygous double mutants segregated 1/4. The inbred line B73 was used for *in situ* hybridization experiments.

For developmental analyses, plants were grown in a greenhouse in Oxford, UK with a 16 h/8 h light regime. Daytime temperature was maintained at 28°C and night-time temperature at 20°C. Supplemental light was provided when natural light was lower than 120 μmol photon m^−2^ s^−1^. Seeds were germinated in warm, damp vermiculite and transferred after 1 week to 12 cm diameter pots containing a 3:1 mix of John Innes No. 3 Compost (J. Arthur Bower) and medium vermiculite (Sinclair Pro).

### Genotyping

A first round of genotyping was undertaken on genomic DNA extracted with a sodium dodecyl sulfate (SDS) high-throughput 96-well plate protocol. Leaf tissue was homogenized with 500 μl SDS extraction buffer [200 mM Tris (pH 7.5), 250 mM NaCl, 25 mM EDTA, 0.5% SDS] in 96-well collection microtubes. Plates were then centrifuged at 6000 rpm (5796 ***g***) for 10 min, and 200 μl supernatant removed and mixed with 200 μl isopropanol in a 96-well polypropylene plate. After 10 min incubation at room temperature, plates were centrifuged and the supernatant discarded. DNA pellets were washed in 70% (v/v) ethanol, centrifuged and air dried before being resuspended in 100 μl distilled H_2_O.

Individuals identified with genotypes of interest were subjected to a second round of genotyping using genomic DNA extracted using a modified cetyl-trimethyl-ammonium bromide (CTAB) protocol optimized to yield high-quality DNA (Murray and Thompson, 1980). Leaf tissue was homogenized at room temperature in CTAB buffer [1.5% (w/v) CTAB, 75 mM Tris-HCl (pH 8), 15 mM EDTA pH 8, 1.05 M NaCl] and heated to 65°C for 30 min. An equal volume of 24:1 chloroform:isoamyl alcohol was added and mixed, before samples were centrifuged. The resultant supernatant was mixed with 2.5 volumes of 100% (v/v) ethanol. The precipitate was collected by centrifugation and washed with 70% (v/v) ethanol before drying and resuspending in 100 μl distilled H_2_O.

The presence of mutant alleles and the sites of insertion were elucidated by PCR using a 1:1 mix of two primers (EOMu1 and EOMu2, Fig. S1B-D) designed to amplify out from both the 5′ and 3′ end of the *Mutator* element, and a primer amplifying from the gene sequence adjacent to the predicted insertion site (Fig. S1B-D). The presence of the wild-type allele was confirmed using a pair of primers that flanked the insertion site (Fig. S1B-D). In some cases, the ‘wild-type’ primers amplified across the transposon producing a larger product size from mutant alleles than from wild-type alleles. However, in most cases there was no amplification with these primers when only the mutant alleles were present. PCR amplifications were carried out in a total reaction volume of 10 µl containing 5 µl of 2×GoTaq master mix (Promega) and 2.5 µl of 4 M betaine. Reaction conditions were as follows: 95°C for 5 min; 35 cycles of 95°C for 30 s, 57-64°C for 30 s and 72°C for 60-90 s; and 72°C for 10 min. All PCR experiments were designed and tested using homozygous single mutant lines to ensure that primers only amplified from the correct gene sequence. All PCR products were assessed by agarose gel electrophoresis.

To determine whether transposons were retained in the transcripts from mutant alleles, RNA was extracted using an RNeasy Plant Mini Kit following the manufacturer's instructions (Qiagen). Extracted RNA was DNaseI-treated using TURBO DNase (Invitrogen) and 2 µg RNA was used for cDNA synthesis using a Maxima First Strand cDNA synthesis kit (Thermo Scientific). RT-PCR was carried out on 1/10 cDNA dilutions using primers amplifying from the transposon to the flanking genomic region (Fig. S1E).

### Analysis of fresh leaf sections

Plants were photographed 32 days after planting, and fully expanded leaf 5 (i.e. the fifth leaf to emerge after germination) was removed at the ligule for phenotypic analysis. Leaf length, width and midrib extension (the point along the proximal/distal axis at which the midrib was no longer visible) were recorded. Segments of leaf encompassing the midrib and the three or four adjacent lateral veins were cut from the midpoint along the proximal/distal axis and positioned upright in 7% agar. Once cooled, blocks were trimmed and mounted such that veins were vertically orientated. Sections (50-60 μm) were cut using a vibratome and then cleared for around 10 min in 3:1 ethanol:acetic acid. Sections were incubated in 70% ethanol overnight, then floated on slides with 70% ethanol (v/v) and covered with a coverslip. Leaf sections were imaged using a Leica DMRB microscope with QImaging MicroPublisher camera (QImaging, www.qimaging.com) and Image-Pro Insight software (MediaCybernetics, www.mediacy.com). Images were taken using bright-field (which enabled BS and M cells to be identified) and UV (which enabled sclerenchyma and thus vein orders to be determined) illumination. Images were tiled together so that the region of leaf between two lateral veins was represented. Subsequent quantification of segment width was undertaken using the ImageJ software package (https://imagej.nih.gov/).

### Tissue fixation and embedding

Segments of primary and seminal roots were fixed in ice-cold 90% acetone for 15 min, rinsed with 100 mM phosphate buffer (pH 7), placed in 3:1 ethanol:acetic acid for a further 15 min and then transferred to 70% (v/v) ethanol. B73 shoot apices were harvested on ice after 7 days growth, prior to the emergence of the first leaf through the coleoptile. Harvested apices were fixed and vacuum infiltrated for 1 min in ice-cold 4% (w/v) paraformaldehyde. Fresh paraformaldehyde was added following vacuum infiltration and samples left overnight. The following day, samples were dehydrated through ice-cold 10%, 30%, 50% and 70% (all v/v) ethanol for 2 h each. Segments of leaf were cut from the midpoint along the proximal/distal axis of fully expanded leaf 5, encompassing approximately three lateral veins adjacent to the midvein. Leaf segments were fixed for 30 min in 3:1 ethanol: acetic acid and transferred to 70% ethanol. All fixed tissue was stored at 4°C in 70% ethanol (v/v), and prior to embedding root samples were placed in 0.7% agar.

Fixed tissue was dehydrated and embedded in paraffin wax using a Tissue-Tek VIP machine (Sakura, www.sakura.eu). Samples were dehydrated at 35°C through 70%, 80%, 90% [with 1% (w/v) eosin], 95% and three times 100% (all v/v) ethanol for 1 h each. Samples were then incubated three times at 35°C in histoclear for 1 h each. Finally, samples were wax infiltrated by four incubations of 2 h in paraffin wax at 65°C. Embedded tissue was then placed in wax blocks and left to solidify at 4°C overnight. Wax blocks were trimmed and 10 μm transverse sections cut using a Leica RM2135 rotary microtome and placed on slides at 37°C to dry overnight.

### Toluidine Blue staining of root sections

Slides were placed in histoclear twice for 10 min each to remove wax, before being taken through an ethanol re-hydration series (1 min in each). Slides were stained in 0.05% (w/v) Toluidine Blue (50 mM citrate buffer, pH 4.4) for 5 s, rinsed in distilled H_2_O and then dried and mounted using a drop of entellen (Merck Millipore). Images were taken using bright-field illumination as above.

### *In situ* hybridization

*In situ* hybridization was carried out using wax-embedded shoot apices as described by Schuler et al. (2018), with digoxygenin (DIG)-labelled RNA probes designed to specifically detect either *ZmSCR1* or *ZmSCR1h* transcripts (Fig. S4A-D). The *ZmSCR1* probe was a 108 bp region towards the end of the first exon, which shared 78% identity with the corresponding region of *ZmSCR1h*. The *ZmSCR1h* probe was a different 108 bp region in the 3′UTR. The current predicted gene model for *ZmSCR1* does not include the majority of this 3′UTR region (Phytozome12), and as such the probe should be highly specific for *ZmSCR1h*. If this gene model is incorrect, and *ZmSCR1* encodes a longer 3′UTR, then the *ZmSCR1h* probe shares 63% sequence identity with the *ZmSCR1* gene (Fig. S3). A sense version of the *ZmSCR1h* probe was used as a negative control (Fig. S4E-H). Post-hybridization washes were undertaken with 0.005× (*ZmSCR1*) and 0.01× (*ZmSCR1h*) SSC buffer made from a 20× SSC stock (3M NaCl, 0.3M Na_3_citrate), calculated to ensure stringency.

### Immunolocalization

Slides were dewaxed twice for 10 min in histoclear, then transferred through 99% (×2), 95% and 85% ethanol (all v/v) for 2 min each. Slides were incubated in 3% (v/v) H_2_O_2_ in methanol for 15 min, and rehydrated through 70%, 50% and 30% ethanol (all v/v) and finally distilled H_2_O twice. Slides were then incubated in PBS/BSA buffer [0.15 M NaCl, 10 mM phosphate buffer (pH 7), 1 mgml^−1^ BSA] for 5 min, drained and incubated for 15 min in 0.1% (w/v) goat IgG (Sigma Aldrich) (in PBS/BSA) and rinsed in PBS/BSA. Slides were then incubated for 15 min with a 1/1000 dilution (in PBS/BSA) of ZmNADP-ME antibody (Langdale et al., 1987), before being rinsed twice for 15 min each in PBS/BSA and incubated for 15 min in a 1/100 dilution (in PBS/BSA) of biotinylated goat anti-rabbit secondary antibody (Sigma Aldrich). Slides were rinsed as before and incubated with a 1/100 dilution (in PBS/BSA) of biotinylated/streptavidin/horseradish peroxidase complex (GE Healthcare) before a final PBS/BSA rinse. SIGMA*FAST* 3,3′-diaminobenzidine (DAB) tablets (Sigma Aldrich) were used to prepare a staining solution as per the manufacturer's instructions, including 0.03% (w/v) NiCl_2_. Slides were covered in staining solution and observed until colour had developed sufficiently (usually ∼1 min), before being rinsed in distilled H_2_O and dehydrated through the original ethanol series. Finally, slides were mounted using DPX (Sigma Aldrich) and visualized using bright-field microscopy in the same way described for leaf and root histology.

### Quantification and statistics

Quantification of leaf segment width was undertaken using the ImageJ software package. Raw phenotypic data gathered during this study are provided in Table S1. Statistical analysis was undertaken using RStudio (www.rstudio.com). Student's *t*-tests were used to test for differences in leaf length, leaf width, midvein extension, vein density and vein order ratios between mutants and the corresponding segregating wild-type lines. Standard errors of the mean were calculated for M and BS data.

### Phylogeny construction

Primary transcript proteomes from eleven species were downloaded from Phytozome12 (Goodstein et al., 2012); *Zea mays* (B73), *Sorghum bicolor*, *Setaria italica*, *Setaria viridis*, *Oryza sativa*, *Brachypodium distachyon*, *Brachypodium stacei* and *Ananas comosus* were chosen for monocots, *Arabidopsis thaliana* and *Solanum lycopersicum* for dicots, and *Physcomitrella patens* as an outgroup. The ZmSCR1 (GRMZM2G131516) primary protein sequence was used as a query in a BLASTp search (evalue of 1e-3) against this proteome database, with the top 100 hits retained. The gene model from one sorghum *SCR* orthologue (Sobic008G023401.1) was incorrect, as it was predicted to begin without a start codon. The upstream region of this sequence was interrogated and an in-frame start codon was identified. No suitable expression data were available to validate this corrected gene model, but as the original model was definitely incorrect, the new version was used for alignment. The 100 sequences (Table S2) were aligned using MergeAlign (Collingridge and Kelly, 2012) and the resultant alignment (Fig. S5) was used to generate a maximum likelihood phylogeny using IQtree (Hoang et al., 2018; Trifinopoulos et al., 2016). In parallel, the alignment was trimmed using trimAl to remove poorly aligned regions, such that columns with fewer than 30% of the sequences represented were discounted from further analysis (Capella-Gutiérrez et al., 2009). Trimming did not alter the topology of the resultant tree, and as such the untrimmed version is presented here. Trees were visualized using the Interactive Tree of Life (iTOL) software (Letunic and Bork, 2016).

## Supplementary Material

Supplementary information
