# Supplementary material for: Redundant SCARECROW genes pattern distinct cell layers in roots and leaves of maize
Source: Development. 2019 Jul 19;146(14):dev177543. doi: 10.1242/dev.177543 (PMC6679360; doi:10.1242/dev.177543)
Supplement: Supplementary information [file develop-146-177543-s1.pdf]

Table S1. Phenotypic data gathered during this study.

Click here to Download Table S1

Table S2. Top 100 BLASTp hits returned using ZmSCR1 as a query.

Click here to Download Table S2

A

Gene	Accession	UniformMu ref	Seed stock	Allele name	Other Mu insertions mapped in seed stock
ZmSCR1	GRMZM2G131516	mu1038565	UFMu-03744	*Zmscr1-m2*	None
ZmSCR1h	GRMZM2G015080	mu1013874	UFMu-01221	*Zmscr1h-m1*	None
ZmSCR1h	GRMZM2G015080	mu1019839	UFMu-01078	*Zmscr1h-m2*	4

B

Primer name	Sequence (5'-3')
SCR1-F2	CCTCCTCCTTCTTCCCCAAA
EOMu1	GCCTCCATTTCGTCGAATCCC
EOMu2	GCCTCTATTTCGTCGAATCCG
SCR1-F4	AACTCACCCCCAACTCCACT
SCR1-AB1-R1	ATGAGCTGCGTGATGGAGAC
SCR1h-F9	CGCTAGCATTGTCGTTCTGGTAC
SCR1h-F11	ACGCACATCCTGACTGACAG
SCR1h-R11	AGATCTACCACTGGCTGGGT
ZmUbiCE-F	TGCGTTAATCACGAGACAGG
ZmUbiCE-R	AATCACAAAGACAGGCAGGG

Primer pair	Product size (bp)
SCR1-F2/ EOMuMix	322
SCR1-F4/ SCR1-AB1-R1	761
SCR1h-F9/ EOMuMix	Zmscr1h-m1 -369<br>Zmscr1h-m2- 312
SCR1h-F11/ SCR1h-R11	588
SCR1h-R11/ EOMuMix	Zmscr1h-m1 -389<br>Zmscr1h-m2- 446
ZmUbiCE-F/ZmUbiCE-R	gDNA- 352<br>cDNA- 267

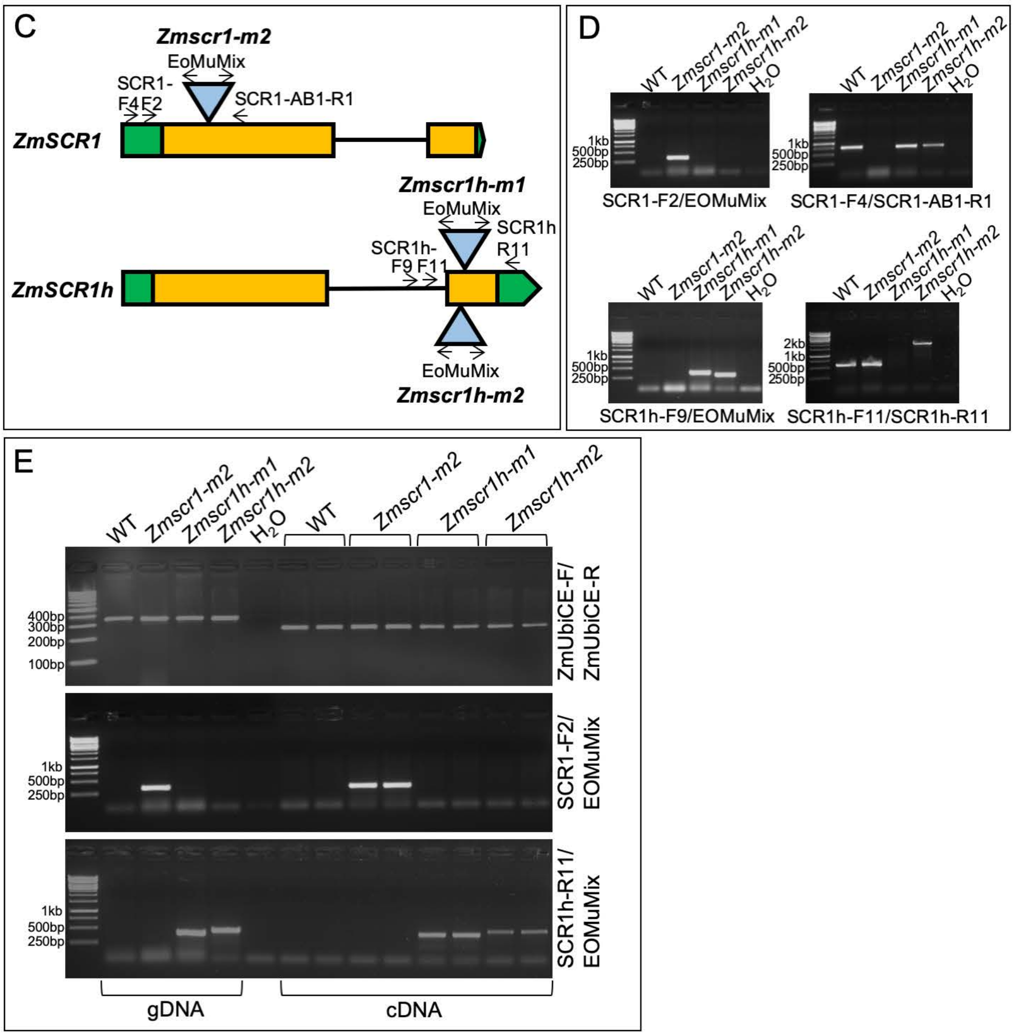

Figure S1. Validation of insertion alleles. A) Details of alleles provided by the UniformMu project. **B)** Primers used in this study. **C)** Schematic representation of each mutant allele, with the position of genotyping primers indicated. **D)** Agarose gels showing fragments amplified from WT and mutant alleles of both *ZmSCR1* and *ZmSCR1h*, with different primer pairs as indicated. Genomic DNA was used as template in all cases. **E)** Agarose gels of fragments amplified from genomic DNA or cDNA (copy of RNA extracted from leaf primordia), using primer pairs as indicated. Transposon sequences are present in the transcripts of each mutant allele.

Development • Supplementary information

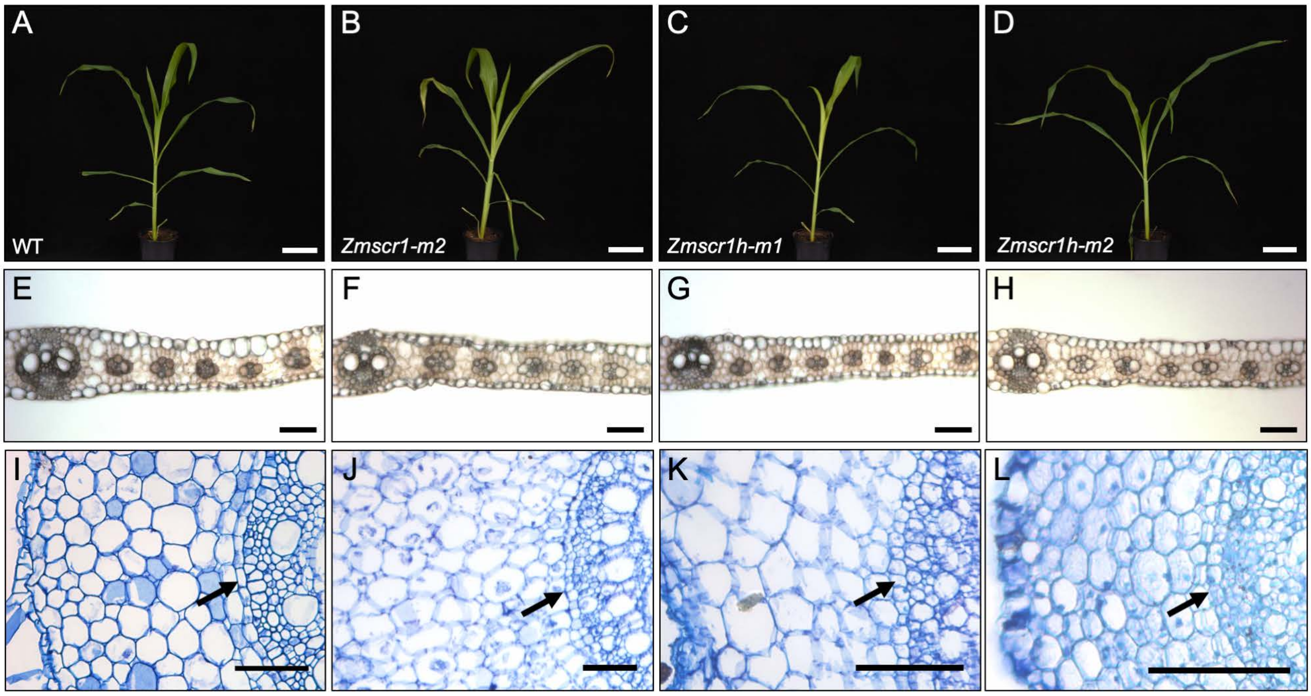

Figure S2. Growth and development characteristics of *Zmscr1* and *Zmscr1h* single mutants. **A-D)** Representative whole plant phenotype at 32 days after planting. **E-H)** Representative transverse sections of fully expanded leaf 5 of WT (E), *Zmscr1-m2* (F) *Zmscr1h-m1* (G) and *Zmscr1h-m2* (H) plants. Representative transverse sections of seminal roots of WT (I), *Zmscr1-m2* (J) *Zmscr1h-m1* (K) and *Zmscr1h-m2* (L) plants. Arrows indicate the endodermal layer positioned between vasculature and cortex. Scale bars = 10cm (A-D); 100µm (E-L).

A	Genotype	Latest emerged leaf 31 days after planting (n=7)	Mean	Mode
	WT (*m2m1*)	8, 8, 8, 8, 7, 8, 8	7.86	8
	Zmscr1-m2;Zmscr1h-m1	6, 7, 6, 7, 7, 6, 7	6.57	7

B	Genotype	n	Total number of veins across leaf width (± s.e.m)
	WT (*m2m1*)	7	201.54 ± 3.13
	Zmscr1-m2;Zmscr1h-m1	7	172.69 ± 8.54
	WT (*m2m2*)	4	273.53 ± 5.51
	Zmscr1-m2;Zmscr1h-m2	5	233.96 ± 11.13

Figure S3. Quantification of *Zmscr1;Zmscr1h* growth characteristics. A) Quantification of the latest emerged leaf in segregating WT and *Zmscr1-m2;Zmscr1h-m1* mutants. **B)** Quantification of the total number of veins across the width of the leaf, calculated by multiplying leaf width by vein density. s.e.m indicates standard error of the mean.

Development • Supplementary information

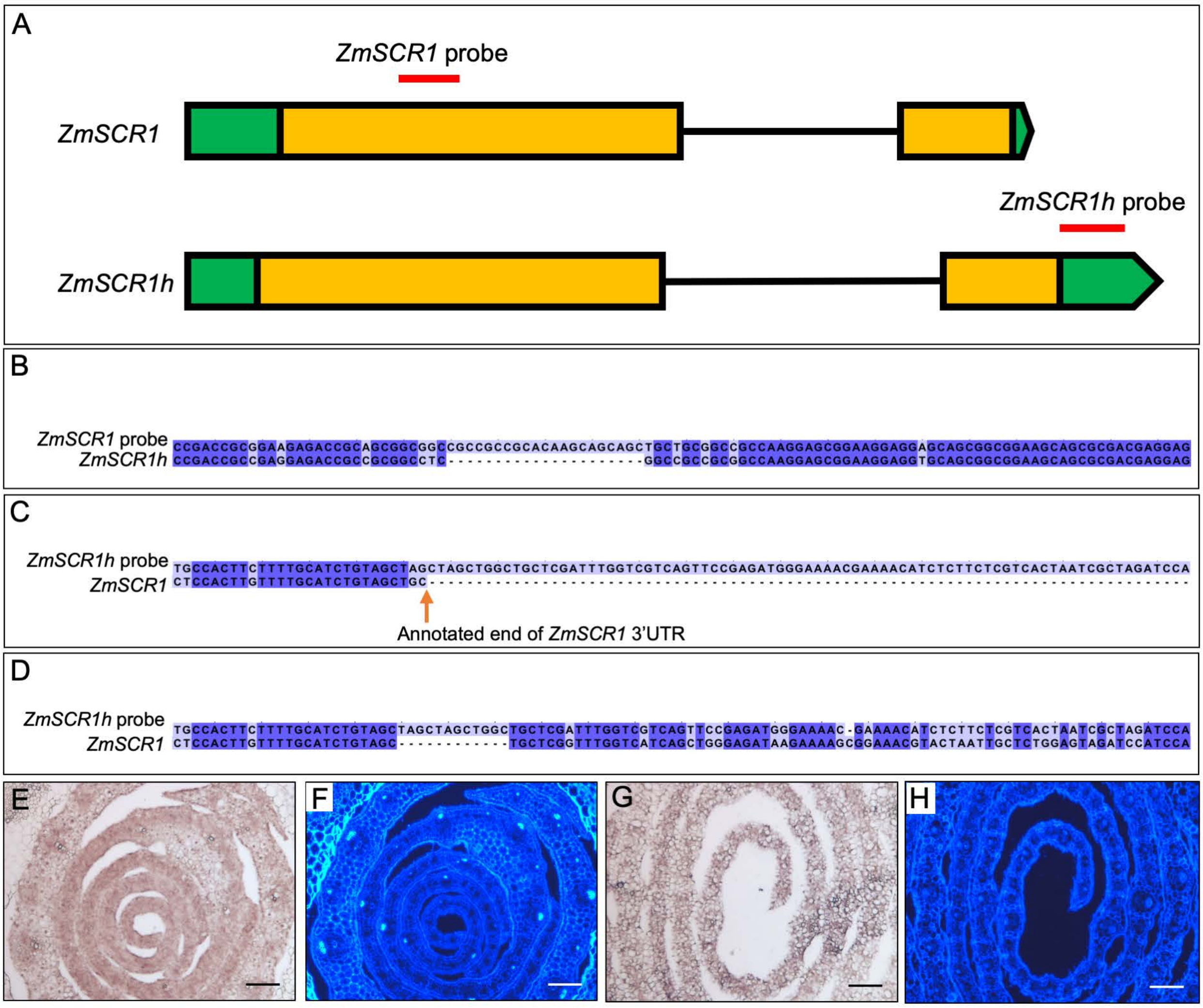

Figure S4. *In situ* hybridization probe design. A) Cartoon showing position of *ZmSCR1* and *ZmSCR1h* probes. Green indicates UTRs; orange indicates exons; black lines indicate introns; Red lines indicate hybridization probes. **B-D)** Alignment of the *ZmSCR1* probe with the *ZmSCR1h* gene sequence (B); the *ZmSCR1h* probe with the *ZmSCR1* annotated gene sequence (C); or the *ZmSCR1h* probe with the annotated *ZmSCR1* gene sequence and region downstream of the of the 3'UTR end (D). Dark blue indicates identical sequence, light blue indicates differences. **E-H)** *In situ* hybridization of sense *ZmSCR1h* probe used as a negative control for background signal accumulation. Cross sections were imaged under both brightfield (A, C) and UV (B, D) illumination to show hybridization signal and calcofluor staining of cell walls respectively. Both younger (A, B) and older leaf primordia (C, D) were hybridized. Scale bars = 100µm.

Development • Supplementary information

Figure S5. Protein alignment of top 100 BLASTp hits returned using ZmSCR1 as a query.

```
>Pp3c18_4830V3.1.p
----------------------------------------------------------------------
----------------------------------------------------------------------
-----------------------------MTLQILSKRSC-----PVE----VGPSAKRW----------
----------------------------KSQSSW-AIEGVAPVAATSAAAEAAEGSACVKQEDPITL---
----------------------------------------------------------------------
-----QEPTSVLDLQ-------------TSPGRSS-SSGSLSSHSSLSGDETSFPSLAGTDICSEDQTSS
ELLPVSTVES-------VHNDHDIASWMECMEDPNGPLALTDFSKCEEGGEVRQ-AEVKLDGGIETGIDH
FVNADIDFGFEL-SLDDNDHEAFANYMLSDPGLCLMDE--------------------------------
------------------------------------PKFLNFPEGFQDAHRLLELGDS--LSSMIDSAPG
IG--------------------------------------------------------------------
----------------------VGEVQSSD-A---------------------SSTPGSDVSYEPGYSGS
QNW--ESNPLEV--------------------QQPLDSGLQLV-HLLLACAEAIEESNFDTARPMLSRLK
AISNPYGDP-MQRISLYFADALSDRLTKESE-------TPVSAAP---------ISSPVELD--TDLAYQ
S-FYE---------------VLPFAKFTHFTANQAIFEAVGYHNKIHVVDLDIQ-QG--L----QWPSFL
QTLA-LRP----GGP----P-SLKITAVGTNAA------SLQLTKRRLSEFAQALEVPFELI-VLV-EDL
DNL-DK------EKF----------QIE-------PDEALAVNCSQVLHRLS-GSEAV---LQKLLLLLR
S-LNPEVVTLLEVEAN--HN--------------GANLISRFVEALHYYCALFDALEAS-----------
-----VS------SDS-PDRFRIENITLASEIRGIVALEGSGRG-AR-HVKS-ETWQSHFTKCGFRNRPL
SSYA-VQQAQLLL--GYFVT------GETPTYKLS---EE--FGVLIMGWQDTPVMAVSSWSC-------
--------------------------
>Solyc09g066450.1.1
----------------------------------------------------------------------
----------------------------------------------------------------------
----------------------------------------------------------------------
----------------------------------------------------------------------
----------------------------------------------------------------------
--------------------------------------MMKGESELIHQHTPLDQPNEQWDYCSPNHPSK
TLITENRANN-------PDQRNELSQWVEQVTRQLLIED-------------------------------
----------------------------------------------------------------------
----------------------------------------------------------------------
----------------------------------------------------------------------
-------LPENEFSDT------DTATYHVS-ALLGELRPKKIARRDCNSEGIGGQQQHEWNSSTHDDHHV
ARDEMGVKGM----------------------SGLDEQGLNLI-TLLLEGAVAISVDNLGEAHRVLLELT
QVASPYGPSCAERVVAYFAKAMASRVINSWL-------GICSPL-------------INYKT--VHTALQ
A-FNN---------------ISPFIKFAHFTSNQAILEAFHRRDRVHIIDVDIM-QG--L----QWPALF
HILA-TRM----EGP----P-HVTMTGVGTSME------LLIETGKQLSNFAKRLGMSFEFH-PVV-GKT
GEI-DI------STF----------KIS-------RGEAIAIHWVQ--HSLY-DATGP---DWKTMRLLQ
Q-LSPRVVTLVEQEIA--L---------------GGSFLDRFVGSLHYYSTIFDSLGAF-----------
-----LE------SDD-SSRHSVEHGLLYREINNILAIGGPAR--NG-EDKF-RHWRSELSKNGFIQVPM
STNS-MAQAQLIL--NMFPP------AH--GYSLV---QG--DGTLRLGWKDTSLFTASAWTSPNSR---
--------------------------
>LOC_Os11g03110.1
----------------------------------------------------------------------
--------MGSSSLLLFPSSSSSAT-------HSSYSPSSSSHAITSLLPPLP-----------------
-----------SDHHLLLYLDH-QEQH-H----------------LAAAMVRKRPASDMD----------
----------------------------------------------------------------------
----------LPPPRRHVTGDLSDVTAAAAPS------------SASAQLPALPTQL-------P-AFH-
-----HTDMDLAAP-------APPPPQQQVAAGEGGP----PSTAWVDGIIRDIIAS----S-GAAVSVA
QLIHNVREII-------RPCNPDLASILELRLRSLL----------------------------------
----------------------------------------------------------------------
--------------------------------------------------------------------TS
```

```
DPAPPPPPPPSHP----ALLPPDATAPPPPPT-SVAALPPPPPPQ---PDKRRR-------E-----PQC
QEQEPNQP----QSPK------PP-----------------------------TAEET--------AAAA
AAAKERKEEQRR--------------------KQRDEEGLHLL-TLLLQCAESVNADNLDEAHRALLEIA
ELATPFGTS-TQRVAAYFAEAMSARLVSSCL-------GLYAPLPNPS-PA----AARLHGR--VAAAFQ
V-FNG---------------ISPFVKFSHFTANQAIQEAFEREERVHIIDLDIM-QG--L----QWPGLF
HILA-SRP----GGP----P-RVRLTGLGASME------ALEATGKRLSDFADTLGLPFEFC-PVA-DKA
GNL-DP------EKL----------GVT-------RREAVAVHWLR--HSLY-DVTGS---DSNTLWLIQ
R-LAPKVVTMVEQDLS--H---------------SGSFLARFVEAIHYYSALFDSLDAS-----------
-----YS------EDS-PERHVVEQQLLSREIRNVLAVGGPAR--TG-DVKF-GSWREKLAQSGFRVSSL
AGSA-AAQAVLLL--GMF-P------SD--GYTLI---EE--NGALKLGWKDLCLLTASAWRPIQAS-GR
--------------------------
>GRMZM5G821439_P01
----------------------------------------------------------------------
----------------------------------------------------------------------
----------------------------------------------------------------------
----------------------------------------------------------------------
----------------------------------------------------------------------
----------------------------------------------------------------------
---------------------MDLHQLLKYRLTGANVYYEFPTENNLANTPWPA----------------
--------SPLKSEFSSSSYTPLSSQLECDNLSALSNTPDNQ-SSTETISAQ---SISPLEVDSSYKQAA
I-LRENTQVRPDPLYT-----TSRHNMQHALREIE-TVLMA---PDADDAATSTKHEFEEHKPAQLMRQR
SRTWSHESRQ------------------------------------------------------------
-----------------------------------------------------PSPGVVRTQLAS-GYPT
ASYEFRPEKRLR--------------------ELREDPQSMVK-QLLTKCAEALSEERIEEFLNLVQQAR
GVVSITGEP-IQRLGAYLLEGLVARHANSGT-------NIYRALK---------CREPESNE--LLSYMK
I-LYN---------------ICPYLKFGYMAANGAIAEALRNEDRIHIIDFQIA-QG--T----QWITLI
QALA-ARP----GGP----P-HVRITGIDDPVSEYARGEGLDLVGKMLKSMSEEFRIPLEFT-PLPGIYA
TQV-TK------EML----------DIR-------SGEALAVNFTLQLHHTPDESVDVNNPRDGLLRMVK
G-LSPKVTTLVEQESH--TN--------------TTPFLMRFTETLDYYSAMFESIDTN-----------
-----LP------RDN-KERINVEQHCLAKDIVNIIACEGKDRV-ER-HELL-GKWRSRLTMAGFRPYPL
SSYV-NSVIRKLL--ACY--------SD--KYTLE---EK--DGAMLLGWKNRKLISASAWH--------
--------------------------
>Pp3c19_8310V3.1.p
----------------------------------------------------------------------
----------------------------------------------------------------------
-----------------------------------MAYQY-----SPGGSRWKPTGGTLV----------
----------------------------DGRLRHDKFTQASDAVQQLEELHTSLGSVSQDSLNIPAY---
----------------------------------------------------------------------
----------------------------------------------------------------------
YTLGSSSQAV-------SNCSTDLAGWIDCMIEELSSNT-------------------------------
----------------------------------------------------------------------
----------------------------------------------------------------------
-----------------------------------------ACPIMAPQQQHGLLEGSFLKNDHDASSCR
DSLLETGSHRLSNVQF------QDTSAARN-K---------------------SSTAPHNGTSQVNAIRT
TAAGLEQQLNKM--------------------GEDENNGIQLV-HSLLACAESIQRGNLSFAEETLRRIE
LLSLPPG-P-MGKVATHFIGALTRRIYGVASSSGNN------------------SSSNQSDS--LLGLLH
FYFYE---------------SCPFLRFAHFTANQAILEAVTGLKEVHVIDFNLM-QG--L----QWPALI
QALS-LRQ----GGP----P-RLRLTGIGPPQP--SGSDTLQEIGTKLAELAKTVRVDFEFRGVIA-VKL
DDI-KP------WML----------QIR-------HGEAVAVNSVLQLHKLL-YSAGPEAPIDAVLLLVR
E-LKPKIFTIVEHEAN--HN--------------QPSFLGRFIEALHYYSTMFDALEAC-----------
----NLP------SEN-NEQV-LIEMYLGREIYNIVACEDGART-ER-HENL-FQWRLRLLKAGYRPIQL
GLNA-FKQASMLL--TMFS-------GE--GYRVE---EK--LGCLTLGWHSRPLIAASAWKCA------
```

```
--------------------------
>Pp3c21_17650V3.1.p
----------------------------------------------------------------------
----------------------------------------------------------------MFIEEQ
IRWIMEVIIWWTWTSRASIRDGFFSSGLAQARSGNVQHLY-----QTQPLLINAEPGNSL----------
----------------------------GKI-ADAVPSHQHQHVPQFDMPQHRTHSFSAHNASENVK---
----------------------------------------------------------------------
--------------------------------DQFAGLDEDAVSQWVDGMIRGMMEV------MPGMPIE
HLFANLSEAL-------APGYVNAERVIRSRLHPLYASARGRVQNTLPIDLARA-GKRPRVEYNDGAKGQ
VYRENANSSQNTSQTF------------------------------------------------------
-------------------------------------KNAISGLHDRQENVMQIQAA---VSHPSPELRE
DQQLPFSSSQKRTDDNLQLSLEPADERSLRHLHTTRQPSL-SKFGHQGVTSRDSPGPSHLKQYPQIQPHY
HQKPKLGNTILDFDVR------SSVSQCSA-Q---------------------NARDCQGLPSLPLDVPQ
TSSQKRQSHE----------------------PSTSQEGLQLL-ALLLQCAEAVSSGNHDEANTILPQLR
EQVTPYGSS-VQRVVAYFAEGMASRLVTSCL-------GINSPLPRN--------DLVNNPS--FTSAIQ
V-FNE---------------ICPFVKFSHFTAIQAISEAFEGMNNVHVIDMDIM-HG--L----QWHLLL
QNLA-KRP----GGP----P-HVHITGLGTSVE------TLDATGKRLIDFAATLGVSFQFT-AVA-EKF
GKL-DP------SAL----------KVE-------FSDALAVHWMH--HSLY-DVSGC---DSATLGLMH
K-LSPKIITIVEQDLR--H---------------GGPFLNRFVEALHYYSALFDSLGAS-----------
-----YN------RKS-LKRHMVEQQLLSCEIKNILAIGGPGR--SG-TTKF-DHWRDKLSEAGFNPVAL
SAQA-VHQAALLLSQGFY-P------GE--GYTLL---ED--LGALKLGWEDLCLFTASAWTST------
--------------------------
>Seita.8G008700.1.p
----------------------------------------------------------------------
--------MGSSSLLLFPSSSSAASPA-----YSHATA--STSSHSSLLPPLP-----------------
-----SPQ----DHHLLQYLHHLDQQQ-Q----------------ESAAMVRKRPAPDMD----------
----------------------------------------------------------------------
----------LPPPRRHVTGDLSDVTAAAASG-GGAPPPQ--SAATSAQLPALPTQL---HL--P-AFQA
Q-QQHHHHAEVDAP----------------PAGEVAA----STTAWVDGIIRDIIGS----SGGAVVSVA
QLIHNVREII-------HPCNPGLASLLELRLRSLLN---------------------------------
----------------------------------------------------------------------
--------------------------------------------------------------------AA
DPAAPLHQPLPHP---------------PALP-PVPALPPPPPPQLTVTDKRRH-------E------PQ
QVGEPTNPSPQQEEPK------PP-----------------------------TAEETAAAAAAAAAAAA
AAAKERKEEQRR--------------------KQRDEEGLHLL-TLLLQCAEAVNADNLDDAHQTLLEIA
ELATPFGTS-TQRVAAYFAEAMSARLVSSCL-------GLYAPLPPAS-PA----AARLHGR--VAAAFQ
V-FNG---------------ISPFVKFSHFTANQAIQEAFEREERVHIIDLDIM-QG--L----QWPGLF
HILA-SRP----GGP----P-RVRLTGLGASME------ALQATGKRLSDFADTLGLPFEFC-AVA-EKA
GNV-DP------EKL----------GVT-------RREAVAVHWLH--HSLY-DVTGS---DSNTLWLIQ
R-LAPKVVTMVEQDLS--H---------------SGSFLARFVEAIHYYSALFDSLDAS-----------
-----YG------EDS-PERHVVEQQLLSREIRNVLAVGGPAR--TG-DVKF-GSWREKLAQSGFRSASL
AGSA-AAQASLLL--GMF-P------SD--GYTLV---EE--NGVLKLGWKDLCLLTASAWRPIQTP-CR
--------------------------
>Seita.9G123000.1.p
----------------------------------------------------------------------
----------------------------------------------------------------------
---MKREYQDA---------GGS--GGDMGSSKDKMMAAA-----AGAGEQDEE-VDELL----------
----------------------------AALGYKVRSSDMADVAQKLEQLEMAMGM---GGAGAAAD---
----------------------------------------------------------------------
--------------------------------------------------------------------DG
FVSHLATDTV-------HYNPSDLSSWVESMLSELNAPPPPLPPA-------------------------
----------------------------------------------------------------------
```

Development • Supplementary information

```
--------------------------------------PPAPRLASTSSTVTGGAAAG---GVYFD-LPP
AVDSSSSTYALKPIPSPA-AASA------------------DP-STDSAREP------------------
--KRMRTGGGSTSSSS------SSSSSLDG-G---------------------RTRSSVVEAAPPAAQAS
AAASGPAVPVVV--------------------VDTQEAGIRLV-HALLACAEAVQQENFAAAEALVKQIP
MLASSQGGA-MRKVAAYFGEALARRVYRFRPAPD---------------------SSLLDAA--FADLLH
AHFYE---------------SCPYLKFAHFTANQAILEAFAGCRRVHVVDFGIK-QG--M----QWPALL
QALA-LRP----GGP----P-SFRLTGVGPPQP--DETDALQQVGWKLAQFAHTIRVDFQYRGLVA-ATL
ADL-EP------FML----------QPD-GEDTDDEPEVIAVNSVFELHRLL-AQPGA---LEKVLGTVR
A-VRPRIVTVVEQEAN--HN--------------SGSFLDRFTESLHYYSTMFDSLEGAGSDNSA-----
ADASPAP------AGG-TDQV-MSEVYLGRQICNVVACEGTERT-ER-HETL-GQWRNRLGRAGFEPVHL
GSNA-YKQASTLL--ALFAG------GD--GYRVE---EK--DGCLTLGWHTRPLIATSAWRAAAP----
--------------------------
>Bradi1g24310.1.p
----------------------------------------------------------------------
----------------------------------------------------------------------
---------------------------MIQGVLSRASAAD-----AAAMK-AKRVAPIP-----------
----------------------------------------------------------------------
----------------------------------------------------------------------
----------------------------------------------------------------------
----------------------------------------------------------------------
----------------------------------------------------------------------
----------------------------------------------------------------------
----------------------------------------------------------------------
-------------EDE------EAAMVGAR-G---------------------KRLQLHSSGVQQQQEA-
-PAEEGKVVVVE--------------------AAAETRGLRLL-SLLLRCAEAVAMDSLPEARDLLHEIA
ELASPFGTS-PERVAAYFGDALCARVLSSYL-------GAYSPLA--LA------SAQQSRR--VASAFQ
A-YNA---------------LSPLVKFSHFTANKAILQALDGEDRVHVVDLDIM-QG--L----QWPGLF
HMLASSRP----SKP---LL-SLRITGLGASLE------VLEATGRRLADFAGSLGLPFEFR-PIE-GKI
GHVADT------DVLLL--------GRD-------EGEATVVHWMH--HCLY-DVTGS---DAGTVRVLR
S-LRPKLVTIVEQDLG--H---------------GGDFLGRFVEALHYYSALFDALGDG-----------
-----AGP---EEEEA-QQRHAVERQLLGAEIRNIVAVGGPKR--TG-EVRV-ERWGDELRRAGFRPVSL
SGGP-AAQARLLL--GMC-P------WK--GYTLV---EE--DGCLKLGWKDLSLLTASSWEPTTTDGDE
EDDLGAAARQEECHGS----------
>Seita.3G200500.1.p
----------------------------------------------------------------------
----------------------------------------------------------------------
----------------------------------------------------------------------
----------------------------------------------------------------------
----------------------------------------------------------------------
----------------------------------------------------------------------
----------------------------------------------------------------------
----------------------------------------------------------------------
----------------------------------------------------------------------
----MSTGRGSDMQDSLSLM-QFHD--PYLYGGGAGGANLPLSSHSFLPHHHD-FVHA---------DDR
CPGK-DVPEFVD-QA---------AAEVSC-E---------------------QELAVSKEVSEGGGDGA
--VEEQNNGAAT----------------TAARGEEEAHGVRMI-ALLMECAVAVSVGNLADANGMLLELA
QMASPYAPSCGERLVAYFTKAMAARLMSSWV-------GICAPLA------------PPFAA--VHAAFR
A-FYN---------------VSPLARFAYLACNQGILEAFHGKCLVHIVDLDVV-PGGAL----QWLSLL
PALA-ARP----GGP----P-VLRVTGFGMSAS------ALHDTGNQLAGLASKLGVPFEFY-AVA-RRP
GD--DADAAAA-AVP----------SRR-------PGEALAVHWLR--HALY-DAAGD---DGATMRLVR
W-LEPKVLTLVEQERG--APGDGGGAGN-----ERGHFLDRFVSALHHYSALFDSLGAS-----------
-----RPA-----DED-ASRHLVEHGVLGREIGNVLAVGGPSR--SG-RDKF-GCWQTELARHGFLRAGG
```

Development • Supplementary information

```
-----GGRAQLVA--GACPA------GL--GYTVA---DD-QDGTVRLGWKGTPLYAVSTWTWCPSPHAQ
R-------------------------
>Pp3c1_2540V3.1.p
----------------------------------------------------------------------
----------------------------------------------------------------------
----------------------------------------------------------------------
----------------------------------------------------------------------
----------------------------------------------------------------------
-----------MSVQYRPELGTMVLTPGYPPGKEREYLSDTANSQQTPSYYGAQKSYADGQRQSAYGMKN
KSHSSPVSPLS------PQDSSQAASDNGQRMSAGWSSASYQSESSSHSDGSLE--------------GP
GKLEEADYYGRQHRHGEQLTGSVAYH--------NTPSSVLRPMGYPAETAQAYQ-------MPNYQQAV
RYIPEEQYAQSQSNYA-----QRNPEMAHMLQVLE-SALLD-----DDDGADLPGSLGNGHDPASEGNWA
DTIEEFMAADASPADSSTVTSATTP---------------------------------------------
-----------------------------------------------------PEYGKQCRNGSTNNYTG
AVTARVEEPPPQ--------------------KLVVGTRSRSE-QLLVACAEALSNNDMPLANVLIAQLN
QVVSIYGDP-MQRLAAYMVEGLVARVAASGK-------GIYRSLK---------CKDPPTRD--LLSAMQ
I-LYE---------------VCPYFKFGYMAANGSIAEAFQNESRVHIIDFQIA-QG--T----QWTTLI
QALA-ARP----GGP----P-HLRITGIDDPMPGPNSNAGVEMVGKRLAKLAEAVGVPFDFH-PVA-KKG
PEV-EA------WML----------ERQ-------PGEALAVNFALHLHHMPDESVCTSNPRDRILHMVK
A-LNPKVVTLVEQESN--TN--------------TAPFFPRFLEAMNYYAAIFESLDIT-----------
-----LA------RES-KERVNVEQQCLARDIVNIIACEGIDRV-ER-HEMM-GKWRARLTMAGFRPYPL
SQTV-NNTIKTLL--ESY--------SD--KYRLK---DE--GGALYLGWKNRSLIVSSAWQ--------
--------------------------
>LOC_Os11g31100.1
----------------------------------------------------------------------
----------------------------------------------------------------------
------------------------------------MAGG-----GAKLQ-QQQAPTSPT----------
----------------------------ASVSESNIVAST-----ASADPEANDALAGLQALRFDGG---
GDIDDVEIQSPDIALWESIFADQIGVSGAGADFLLS------MSSAAASPRRDFMAC-------------
-----SPKRDYMVTT-------------SSPKRDYMVTSSPKR-DYMVSSPKREYMVTSPRREM-ATSPR
RATFSNLYTS-------SHGGGGGGGHHLHHQSYVHG-GGMEGGGGGHGAQPQY-GGLAGH-----GKGK
AQ-SPLHKVYINNVGGGSGGGGVKSNTPSTLSCASSYVVHGGESGLPSLPSMDP-FLE----------EG
YLGSYQLPEKA--AGGVGGGGGGDINRSGASVSVVTAPASSQLLPTLSECLAMPEPAA--YRGGGDEAVA
AAMAVAGELPVGAFVQPEMYYGGGGEFGGE-----GMTP-------------------------------
-------PLQHQM--------------AAD-S---------------------SLHSMLGS---------
----VIQSEA----------------------EQEQDSGLQLV-HLLLACADLVSKGDHPAALRHLHLLR
RVASPLGDS-MQRVASHFADALAARLSLLSSPTS-------ASPSPRAAAAAAPYPFPPSPE--TLKVYQ
I-LYQ---------------ACPYIKFAHFTANQAIFEAFHGEDRVHVVDLDIL-QG--Y----QWPAFL
QALA-ARP----GGP----P-TLRLTGVGHPPA------AVRETGRHLASLAASLRVPFEFHAAAA-DRL
ERL-RP------AAL----------HRR-------VGEALAVNAVNRLHRVP---SSH---LPPLLSMIR
D-QAPKIITLVEQEAA--HN--------------GPYFLGRFLEALHYYSAIFDSLDAT-----------
-----FP------AES-TARMKVEQCLLAPEIRNVVACEGAERV-AR-HERL-ERWRRLMEGRGFEAVPL
SAAA-VGQSQVLL--GLYGA------GD--GYRLT---ED--SGCLLLGWQDRAIIAASAWRC-------
--------------------------
>Solyc07g043330.1.1
----------------------------------------------------------------------
----------------------------------------------------------------------
-------------------------MLFVSAIPINHTSSY-----SSSMS-SKRSISEFT----------
----------------------------------------------------------------------
----------------------------------------------------------------------
----------------------------------------------------------------------
----------------------------------------------------------------------
```

Development • Supplementary information

```
----------------------------------------------------------------------
----------------------------------------------------------------------
----------------------------------------------------------------------
------------PVSD------EPQLLTKR----------------------------------PRNERG
EEEEEEGEELLL--------------------VDADSIGLRLL-GLLLQCAEFVAMENLDEAADLLPEIA
ELSSPFGSS-AERVAAYFAESLSARIISSHL-------RFYSPLN--LKSL----TLTHSQK--LFTALQ
S-YNT---------------ISPLIKFSHYTANQAIYQALEGEDHVHVIDLDIM-QG--L----QWPGLF
QILS-SRS----RKL----R-SIRITGVGSSME------LLESTGRRLTEFANSFGLPFEFQ-PFE-GKI
GHITDL------NQL----------GVK-------IGETTVVNWMH--HCLY-NITGS---DLGTFRLLT
L-LRPKLITLVEQDLS--H---------------GGNFLSRFVEALHYYSALFDALGDG-----------
-----LS------EES-AERHRVEQQLFGSEIRNIVAVGGPKR--TG-EVPV-ERWGDELKRIGFLPVSL
SGTP-AAQASLLL--GMF-P-------R--GYTLV---EE--NGCLKLGWKDLSLLTASAWQPCD-----
--------------------------
>Pp3c2_21080V3.1.p
----------------------------------------------------------------------
--MLFTGELDEVFRLPAVKTGRSSSPSQPRSGVSSPHGAPGPGSSPGLSGSPSYRGVVGGNNRVLSPGKF
MLKKGLEPRQIIERRGEQLSNTDTAGSPSFRRAGTTMWPD-----QTATTCEAEKSSGQW----------
----------------------------RSPRCGEDHRSEGEPGGFFHQHQNQSASNSPLCPGTPIR---
----------------------------------------------------------------------
------------------------------QHYPRTAPSTPLQGISSPDVKKCIAPMSEPRSVLDLTISP
QQHQLPTIRA-------TLPRRFRETFLDHNVAPVAAPSLSSSPRVLNFESCGS-VSPISGPFPSQYGPS
LAKTTTWAPDLATQGYPTSITTHKVEELSVLPTSQNYVQATTSS--------------------------
------------------------------LDEWEAATTTSTQHNLRAKQWMASLMED--LRNGDYSTSA
QETEECTTPFTPGVDSCSPHFLAFQR--------------------------------------------
-------GHSSPCPSD------YTGDDNPG-G---------------------SGLEYLQVESPGGTPRG
GGDGDVDDTH----------------------NSPAVVGHELV-TLLIACAEAVSTQSLSLVNHLLPKLG
ELASPQGTA-MQRVAAYFTEGLACRVAHLWP-------HIYQPLPI--------ESSLNEEE--LQTAFH
L-LNH---------------VVPYTKFAHFTANDIILQGFEGADRVHVIDFDVK-QG--L----QWPALF
QSLA-VRE----CGP----PSHIRITGIGECKE------DLLETGDRLAEFAEEFNIPFTFH-AVI-DRL
EDV-RL------WML----------HVK-------ENEAVAVNCISQLHRLLYDSGET---IEGFLNLIG
S-TKPKVVAVVEQEGS--HN--------------SPQFEGRFLESLQYYSAVFDSLEAN-----------
-----IS------RES-SARVQVEQ-LFAREIRNILSCEGTDRM-ER-HENI-SRWRSIMSRSGFVKVPL
EDSA-YTQALILL--RMFD-------SD--GYTLA---EE--NGAVTLGWMEQPLLTASAWKPDKDFVPT
GTSLVTNEKQRAV-------------
>Seita.8G129500.1.p
----------------------------------------------------------------------
----------------------------------------------------------------------
---------------------------MGMLNCADTSS-------GAKLQ-QLQAPTSPT----------
----------------------------ASVSESNIVVS-------STDPDANDALAGLQALKFD-----
GDI-DVEIQSPDIAMWESLFAEQMGAS--GGDFLMSFSPRRDFTATGSP-RRDFMVS-------------
-----SPKRDYMM---------------SSPKRDYMV-SSPKR-DYMMSSPKREYMVTSPRRD---SSPR
RSTFSNLYSG-------TGS---------HQQGYVDGVHGAEGGSGSGGGQPLY-GGLANH-----GKGK
SQ-SPLHKVYINN--AHSNGG--RSTGPSSLSCSSSY-GHG---ESLSLPSMDP-FLD-----EYKEGGG
YLAGYQLPVKT--------GMENGAASAATVTTVAPSPSQ---LPTLSECLAMPEPVY----GGSEAAAA
G------GLQMG-AGLPAELYYGGQFGGDGF---------------------------------------
-------TLQHQMAKS------DQW--AGD-S---------------------SLHSMLGS---------
----VIQTEA----------------------EQEQDSGLQLV-HLLLACADFVSKGDQPSALRHLHLLR
RVASPLGDS-MQRVASYFADALAARLSLSSNPSSSSSSSGAATPRGGAAAGVAPYTFPPSPE--TLKIYQ
I-LYQ---------------ACPYIKFAHFTANQAIFEAFAGEDRVHVVDLDIL-QG--Y----QWPAFL
QALA-ARP----GGP----P-TLRLTGVGHPAA------AVRETGRHLASLAASLRVPFEFHAAAA-DRL
ERL-RP------AAL----------QRR-------VGEALAVNAVNRLHRVP---SAH---LGPLLSMIR
D-QAPKIMTLVEQEAG--HN--------------GPYFLGRFLEALHYYSAIFDSLDAT-----------
```

```
-----FP------ADS-APRMKVEQCLLAPEIRNVVACEGAERV-AR-HERL-DRWRRLMEGRGFEPVPL
SPAA-IGQSQVLL--GLYGA------SD--GYRLT---ED--KGCLLLGWQDRAIIAASAWQC-------
--------------------------
>Solyc07g065270.1.1
----------------------------------------------------------------------
----------------------------------------------------------------------
----------------------------------------------------------------------
----------------------------------------------------------------------
----------------------------------------------------------------------
--------------------------------------------------------MRPSQNPEEINEFN
KFYNQPIE---------YQESYFLPSVNNPNNNQSFYADVSALKPA------------------------
---------QHCYVESSAGNSSELVS--------DSPPIVN----FNPMMNQGSDSC-----RSDMHHSP
DDT--FQS--SGNSSC---YTSDVTDLKHKLRELE-TAMLG---PDSESLESFNNTIP--VTAANQVP--
----------------------------------------------------------------------
----------------------------------------------------------------------
----SESDKLVG--------------------MMEMMPSGDLK-EVLIACAKAIAENNLITAEWLMSELR
TVVSVCGSP-IQRLGAYMLEGLVARLASSGS-------SIYKALR---------CKEPTSVE--LFSYMH
L-LYE---------------ICPYFKFGYLSANGAIVDAMKDENSIHIIDFQIA-QG--S----QWITLI
HALA-ARP----GGP----P-RIRITGIDDSTSAYARGGGIEIVGRRLSSIAASCNVPFEFH-PVS-ASC
PDI-EI------EHL----------KVL-------PGEPLAVNFALVLHHMPDESVGTQNHRDRLLRMVK
S-LSPKIVTLVEQESN--TN--------------TAQFFPRFLETLNYYLSVFESIDVA-----------
-----LP------RDH-KERINVEQHCLAREIVNILACEGAERV-ER-HELL-ERWRSRFAVAGFKPYPL
SSSV-NATIKTLL--ENY--------YQ--SYTLN---ER--NGALYLGWMNRDLVASCAWK--------
--------------------------
>Solyc02g094340.1.1
----------------------------------------------------------------------
----------------------------------------------------------------------
---------------------------MGSLKNDIIKNEV-----GSEITSFKDQNSYIK----------
----------------------------LLPEDSLASSESKKVTPISSDFELDCGSLIPTSLTFPPG--G
GDDDDVEIQSPDNSIWESFFADQLEA--------------------------DFMIS-------------
-----SPVRNL--------------------------------------------------SSTSTFCTT
TTTHNNNTYT-------HHNQG-------------------IHGQSMMMCSPPR-SPLRPNNYNSTNKGK
GL-SPFQKVF------------------------NSPNNQFMQIESFNLPALES-FLDDDLASEYSTLKV
SDVGS-------------------------------SSESLSVIPDFLECLALPNSSS--NTSASFMGSL
LSNTSVGQ---------------------------VDDEIFQTGSIAPLSQQ------------------
-------LHQERHHEK------QQK---QI-P---------------------THVQLPSTQQQYTQMIN
HNLVVAAPDQ----------------------MQEQDSGLQLV-HLLLACAEAVSKEDYMLARRYLHHLN
RVVTPIGDS-MQRVASCFTEALTARLAATLATKPST--SVPKPFN---------PFPPNSLE--ILKIYQ
I-LYQ---------------ACPYVKFAHFTANQAIFEAFEAEERVHVIDLDIL-QG--Y----QWPAFM
QALA-ARP----GGA----P-FLRITGVGSYPE------AVRETGRCLTELAQSLHVPFEFH-PVG-EQL
EDL-KP------HMF----------NRR-------IGEALAVNSVNRLHRVP---GNC---IGNLLGMIR
D-QAPNIVTIVEQEAS--HN--------------GPYFLGRFLEALHYYSAIFDSLDAT-----------
-----FP------GDS-SQRAKLEQYIFGPEIMNIVSCEGMERM-VR-HERL-EKWRRVMEGKGFKGVAL
SANA-VTQSKILL--GLYS-------CD--GYKLT---ED--NGCLLLGWQDRAILAASAWRC-------
--------------------------
>Aco003635.1
----------------------------------------------------------------------
----------------------------------------------------------------------
--------------------------------MGGLKSSG-----TSSVVKQKHPPASPT----------
----------------------------ASVSESNIVTSSDPEHLQPDQPERNDAIAIPE-------LKF
ELDLGLEVQSPDNAMWDSLFADVAGA--------------------------DFMIE-------------
-----SPRRDYMV---------------CSPRRDYMA-CSPKRENVTISSPKRTFLM-------------
```

```
---NNNSYSS-------YHNNN---------------YAYVSGVHGLFGCTTQT-TYLSPNH----NKGK
SL-SPLHKVFNYS----------SSSSTSLLPLYASSAEFVRGDESLLLPVVDA-FLD-----EYKEGSN
AEVGFDVP----------------------------GSAATSALPTLLECLSMPEMNQYGEDAVAAGEAA
AGAQGGGE---------------------------GTDHVYQNWELQQLASC------------------
-------GGELADVAK------SEW---AD-P---------------------SYQDVLGA---------
---IVPPSDT----------------------EQEQDSGLELV-HLLLACAEAIAKDDYLAARRYLHHLH
RVVSPVGDS-MQRVASCFADALPLPAVPRRPP------HLPDSLPGR-----------------STRTSR
I-IYHDIRCTTRWSHILTAVVCPYVKFAHFTANQAIFEAFEGEDRVHVVDLDIL-QG--Y----QWPAFL
QALA-ARP----GGA----P-SLRITGVGHSAA------AVSETGRHLAELAHSLRVPFEFHAAAA-GRL
EDL-RP------RML----------HRR-------VGEALAVNAVNRLHCVA---APH---LGPLLAMIR
D-QAPKIVTLVEQEAS--HN--------------GPSFLGRFLEALHYYSAIFDSLDAT-----------
-----LP------GDS-AARMKVEQYLFGPEIRNIVACEGAERM-AR-HERL-ERWRKVMEGKGFHGVPL
SANA-VNQSRLLL--GLYTN------CD--GYRLT---ED--NGCLLLGWQDRALIAASAWRC-------
--------------------------
>Seita.2G369400.1.p
----------------------------------------------------------------------
----------------------------------------------------------------------
----------------------------------------------------------------------
----------------------------------------------------------------------
----------------------------------------------------------------------
-----------------------------------------MADTPTSRMV-HPFGNVPRQTPKQFLYSG
NTQHLCHP---------YQSASDTHVVPEHHYTMK----SHSP-DAVSEEHETR----------------
----------KQYTLDSSAASGCSRH--------DSPSSQSIHTG----------SGSPL--SHEDSHS-
GSTNGNGS--PVSASC--VT-EDPTDLKQKLKDLE-AVMLG---TDSEIVDSLEISVA--NQLS------
----------------------------------------------------------------------
----------------------------------------------------------------------
----LEPEKWV---------------------HMMSMPKGNLK-ELLIACARAVEQNNSFAIDLMIPELR
KMVSVSGEP-LERLGAYMVEGLVARLASSGN-------SIYKALK---------CKEPKSSD--LLSYMH
F-LYE---------------ACPYFKFGYMSANGAIAEAVKGEDRIHIIDFHIA-QG--A----QWISLL
QALA-ARP----GGP----P-FVRITGIDDSVSAYARGGGLELVGRRLSHIAGLYKVPFQFN-AVA-ISG
NEM-EE------GHL----------GIV-------PGEAVAVNFTLELHHIPDETVSTANHRDRILRLVK
S-LSPKVLTLVEQESN--TN--------------TAPFAQRFAETLDYYTAIFESIDLA-----------
-----LP------RED-RERINMEQHCLAREIVNLVACEGEERV-ER-HEVF-GKWKARLMMAGFRPSPL
SALV-NATIKTLL--QSY--------SP--DYKLA---ER--DGVLYLGWKNRPLIVSSAWH--------
--------------------------
>AT3G54220.1
----------------------------------------------------------------------
-----MAESGDFNGGQPPPHSPLRTTSSGSSSSNNRGPPPPPP---------------------------
---------------------------------------------PPLVMVRKRLASEMS----SNPDYN
NSSRPPRRV-------------------SHLLDSNYNTVTPQQPPSLT----------------------
-----------------------------AAATVSSQPNPPLSVCGFSGLPVFPSDR-------------
-----GGRNVMMSVQPM---------------DQDSSSSSASPTVWVDAIIRDLIHS------STSVSIP
QLIQNVRDII-------FPCNPNLGALLEYRLRSLM----------------------------------
----------------------------------------------------------------------
----------------------------------------------------------------------
-LLDPSSSSDPSPQTFEPLYQISNN--------------PSPPQQQQQHQQQQQ-------QHKPPPPPI
QQQERENS----STDA------PP-----------------------------QPETVTATVPAVQTNTA
EALRERKEEIKR--------------------QKQDEEGLHLL-TLLLQCAEAVSADNLEEANKLLLEIS
QLSTPYGTS-AQRVAAYFSEAMSARLLNSCL-------GIYAALPSRWMPQ----THSL--K--MVSAFQ
V-FNG---------------ISPLVKFSHFTANQAIQEAFEKEDSVHIIDLDIM-QG--L----QWPGLF
HILA-SRP----GGP----P-HVRLTGLGTSME------ALQATGKRLSDFADKLGLPFEFC-PLA-EKV
GNL-DT------ERL----------NVR-------KREAVAVHWLQ--HSLY-DVTGS---DAHTLWLLQ
```

```
R-LAPKVVTVVEQDLS--H---------------AGSFLGRFVEAIHYYSALFDSLGAS-----------
-----YG------EES-EERHVVEQQLLSKEIRNVLAVGGPSR--SG-EVKF-ESWREKMQQCGFKGISL
AGNA-ATQATLLL--GMF-P------SD--GYTLV---DD--NGTLKLGWKDLSLLTASAWTPRS-----
--------------------------
>AT2G01570.1
----------------------------------------------------------------------
----------------------------------------------------------------------
---MKRDHHQFQGRLSNHGTSSSSSSISKDKMMMVKKEED-----GGGNMD-----DELL----------
----------------------------AVLGYKVRSSEMAEVALKLEQLETMMSNVQ------------
----------------------------------------------------------------------
--------------------------------------------------------------------ED
GLSHLATDTV-------HYNPSELYSWLDNMLSELNPPPLPASSNGLDPVLP------------------
----------------------------------------------------------------------
-------------------------------------------------------------------SPE
ICGFPASDYDLKVIPGNA-IYQFPAIDSSSSSN--------NQNKRLKSCSSPDSMVTSTSTGTQIGGVI
GTTVTTTTTTTTAAGE------STR---------------------------------------------
--------SVIL--------------------VDSQENGVRLV-HALMACAEAIQQNNLTLAEALVKQIG
CLAVSQAGA-MRKVATYFAEALARRIYRLSPPQN-----------------------QIDHC--LSDTLQ
MHFYE---------------TCPYLKFAHFTANQAILEAFEGKKRVHVIDFSMN-QG--L----QWPALM
QALA-LRE----GGP----P-TFRLTGIGPPAP--DNSDHLHEVGCKLAQLAEAIHVEFEYRGFVA-NSL
ADL-DA------SML----------ELR-----PSDTEAVAVNSVFELHKLL-GRPGG---IEKVLGVVK
Q-IKPVIFTVVEQESN--HN--------------GPVFLDRFTESLHYYSTLFDSLEGV-----------
-------------PNS-QDKV-MSEVYLGKQICNLVACEGPDRV-ER-HETL-SQWGNRFGSSGLAPAHL
GSNA-FKQASMLL--SVFNS------GQ--GYRVE---ES--NGCLMLGWHTRPLITTSAWKLSTAAY--
--------------------------
>AT1G50600.1
----------------------------------------------------------------------
----------------------------------------------------------------------
----------------------------------------------------------------------
----------------------------------------------------------------------
----------------------------------------------------------------------
----------------------MRLSVFIIPLVESRQASGIINKQSTSLLIRFSLYLEASISTKSFFSKS
QRISQTQSPICLSANYYQPDNLDMEATQKHMIQEGSSMFYHQPSSVKQMDLSVQ----------------
-------TFDSYCTLESSSGTKSHPCLNNKN---NSSSTTSFSSNESPISQANNNNL-----SRFNNHSP
EEN--NNS--PLSGSS--ATNTNETELSLMLKDLE-TAMME---PDVDNSYNNQGGFG--QQHG------
----------------------------------------------------------------------
----------------------------------------------------------------------
----VVSSAMYR--------------------SMEMISRGDLK-GVLYECAKAVENYDLEMTDWLISQLQ
QMVSVSGEP-VQRLGAYMLEGLVARLASSGS-------SIYKALR---------CKDPTGPE--LLTYMH
I-LYE---------------ACPYFKFGYESANGAIAEAVKNESFVHIIDFQIS-QG--G----QWVSLI
RALG-ARP----GGP----P-NVRITGIDDPRSSFARQGGLELVGQRLGKLAEMCGVPFEFH-GAA-LCC
TEV-EI------EKL----------GVR-------NGEALAVNFPLVLHHMPDESVTVENHRDRLLRLVK
H-LSPNVVTLVEQEAN--TN--------------TAPFLPRFVETMNHYLAVFESIDVK-----------
-----LA------RDH-KERINVEQHCLAREVVNLIACEGVERE-ER-HEPL-GKWRSRFHMAGFKPYPL
SSYV-NATIKGLL--ESY--------SE--KYTLE---ER--DGALYLGWKNQPLITSCAWR--------
--------------------------
>Aco011304.1
----------------------------------------------------------------------
----------------------------------------------------------------------
----------------------------------------------------------------------
----------------------------------------------------------------------
----------------------------------------------------------------------
```

Development • Supplementary information

```
---------------------------------------------------------MSTSYGDTNLSAR
KSSSERMQMFISDERFHYRANSYLSDIYAQAYFTNSSRVHTSHREV------------------------
----------------SSAPYDLMAN--------TRSSIILNTPYNSCFSSAKQPDSSLS--SNISHHSS
QSVLDNRS--SEHEVD-----FTEDDIRLKLQELE-QALLNDNDPDQD--------------MGIDEEWV
EPIKELLV--------------------------------------------------------------
-------------------------------------------------------------------ASS
PKESSSESIIYC--------------------IGNRKEAQTPK-QLLFDCAAAILEDSIEEAQAIINELR
HMVSIQGDP-PQRLAAYLVEGLAARIASSGQ-------GLYKALK---------CKEPPTSD--RLSAMQ
I-LFE---------------VCPCFKFGFMAANYAITEAFKGEERVHIIDFDIN-QG--S----QYITLI
QSLS-TMS----NNP----P-HLRITGVDDPESVQRAVGGLKIIGQRLEKLAEGCGVPFEFR-AIA-SRT
EDV-TP------AML----------DCR-------PGEALLVNFAFQLHHMPDESVSTVNQRDQLLRMVK
G-LGPKLVTVVEQDVN--TN--------------TAPFFPRFVEVYNYYSAVFNSLDAA-----------
-----LP------RES-ADRMNVEKQCLARDIVNIVACEGADRI-ER-YEAA-GKWRARMTMAGFVSCPF
NANS-SGSIRSLL--KSY--------CD--RYKVK---EE--GGALYFGWEDKTLVVASAWQ--------
--------------------------
>GRMZM2G106548_P01
----------------------------------------------------------------------
----------------------------------------------------------------------
---------------------------MLQGVLSRAPATD--A--AAAMK-AKRAGASPG----------
----------------------------------------------------------------------
----------------------------------------------------------------------
----------------------------------------------------------------------
----------------------------------------------------------------------
----------------------------------------------------------------------
----------------------------------------------------------------------
----------------------------------------------------------------------
-------------EEE------EGDGRSAR-G---------------------KRQQLLGLGPATALASA
AAAE----------------------------EGPETRGLRLL-SLLLRCAEAVAMDQLTEARELLPEIA
ELASPFGSS-PERVAAYFGDALCARVLSSYL-------GAYSPLA--LRPL----AAAQSRR--VAVAFQ
A-YNA---------------LSPLVKFSHFTANQAILQALDGEDCLHVIDLDIM-QG--L----QWPGLF
HILA-SRP----RKP----R-SLRITGLGASLD------VLEATGRRLADFAASLGLPFEFR-PIE-GKI
GHVADA------AALLGSRQ-----RRR-------DDEATVVHWMH--HCLY-DVTGS---DVGTVRLLR
S-LRPKLITIVEQDLG--H---------------SGDFLGRFVEALHYYSALFDALGDG-----------
-----AGA---AEEES-AERYAVERQLLGAEIRNIVAVGGPKR--TG-EVRV-ERWSHELRHAGFRPVSL
AGSP-AAQARLLL--GMY-P------WK--GYTLV---EE--DACLKLGWKDLSLLTASAWEPADDAAAS
APTG----------------------
>Pp3c1_15560V3.1.p
-------------------------------------------------------------MREIGVLEY
GEMLYTGELDELFRLPAVKTGRSSSPSLPCSNASSQYGLHSQRSELGLSGSPSHRGGMSGNNRVLLPGKF
VLKKGLEPWSTVERRGEQLSSTDPAGSPSFRRAGTTMWPD-----KTSTACEAETSTGQW----------
----------------------------TSPRCGEDPRNEGDL-IHFHQHQNQSTSNSPLCPGTPIR---
----------------------------------------------------------------------
------------------------------HHYSQMAPSSPLQIISSPDVKKCIAPMSEPRSVLDLTISP
QQHQLPTIRA-------TLPRRFRETFLEHNVAPVTAPSLSPSPRVLNFESCSN-GSTKSGTFADQYGPS
PTNASTWAPDLATQGYPTSIPTHKVEELPVLPTAQSFIQ-ETSS--------------------------
------------------------------LDEWEAATITSPQHKLRAKQWMVSLMED--LRNAEDANSA
QETEECTTPFTPGVDSCAPYFLTSQN--------------------------------------------
-------GLGSPCPSD------YTGDDTPR-G---------------------SRLEHLQVESPCGTPRG
GGDG--EDAQ----------------------NSPVMVGHELV-TLLIACAEAVSTQSLSLVNHLLQKLG
EHASPQGTA-MQRVAAYFTEGLACRVAHLWP-------HVYQPLPT--------HSNLNDEQ--LQTAFH
L-LNH---------------VVPYTKFAHFTVNDIILQAFNGADRVHVIDFDIK-QG--L----QWPALF
QSLA-ERE----CGP----PSHIRITGIGECKD------DLLETGDRLAEFAEEFNIPFSFH-AVI-DRL
```

```
EDV-RL------WML----------HVK-------ENEAVAVNCISQFHRLLYDSGET---IKDFLNLIG
S-TKPRVVAIVEQEGS--HN--------------SPHFEGRFLESLKYYSAIFDSLEAN-----------
-----LS------RES-CVRVQVEQ-LFALEIRNILSCEGAERV-ER-HEDT-ARWSVLLSQSDFVNVPL
EDSA-NTQAQILL--RMFD-------SD--GYTLT---AE--NGSLTLGWVEQPLLTVSAWKPDKDFVLT
GKSLVTNEKQQAV-------------
>Bradi2g22010.1.p
----------------------------------------------------------------------
----------------------------------------------------------------------
----------------------------------------------------------------------
----------------------------------------------------------------------
----------------------------------------------------------------------
----------------------------------------------------------------------
----------------------------------------------------------------------
----------------------------------------------------------------------
------------------------------------------------------------------MINH
LMSGRVVSDMHHHDSSLGLM-QFHD--PHQYMYSSSSSTA---GGSFLPHHHAAFI-----------KDV
APAE-PLVEILE-EAP------GAAGAPAM-QE--------------------ERKVVDEAA--------
-----EEEEGEE--------------------AAAESHGVRMI-ALLMECAVAMSVGNLADANGALLELS
QMASPYAASCGERLVAYFARALAARLMSSWV-------GVCAPLSLQ-------QHDDAGIV--IHAAFR
A-FYN---------------VAPFARAAYLACNQAVLDAFRGQRAVHIVDLDMV-PGGAL----QWLSLL
PALA-ARP----GGP----PALLRVTGFGVSAA------LLHDAGNQLAGLAGKLGLPFEFY-AVA-KRP
GDA-AAAVSSGLLLP----------GKR-------PGEAVAVHWLR--HALY-DAAGD---EAAAIRLAR
W-LEPRVMTVVDQERSLSSSSSSGAAADD-----GGSFLDRFVSALHHYSAAFDSLGAA-----------
-----RPA-----GDD-ASRHLAENGMLGREIGNVLAIAGPSRS-GRERLLP-GSWQAELARHGFLRARW
GSG--GARAQMLA--GACPA------GL--GYTVA---DDAHDGTVRLGWKGTPLYAVSTWTWCATTHGH
--------------------------
>Solyc11g011260.1.1
----------------------------------------------------------------------
----------------------------------------------------------------------
---MKRDRDRDRERE-----KRAFSNGAVSSGKSKIWEED-----EEEKPDAG--MDELL----------
----------------------------AVLGYKVKSSDMADVAQKLEQLEMAMGTTM------------
----------------------------------------------------------------------
--------------------------------------------------------------------ED
GITHLSTDTV-------HKNPSDMAGWVQSMLSSISTN--------------------------------
----------------------------------------------------------------------
----------------------------------------FDMCNQENDVLVSGCGSS---SSIIDFSQN
HRTSTISDDDLRAIPGGA-VFNS------------------DSNKRHRSTTS------------------
--SFSTTSSSMVTDSS------ATR---------------------------------------------
--------PVVL--------------------VDSQETGVRLV-HTLMACAEAVQQENLTLADQLVRHIG
ILAVSQSGA-MRKVATYFAEALARRIYKIYPQDS------------------------MESS--YTDVLQ
MHFYE---------------TCPYLKFAHFTANQAILEAFTGCNKVHVIDFSLK-QG--M----QWPALM
QALA-LRP----GGP----P-AFRLTGIGPPQP--DNTDALQQVGWKLAQLAETIGVEFEFRGFVA-NSL
ADL-DA------TIL----------DIR-----PSETEAVAINSVFELHRLL-SRPGA---IEKVLNSIK
Q-INPKIVTLVEQEAN--HN--------------AGVFIDRFNEALHYYSTMFDSLESSGSSSSASPT--
GILPQPP------VNN-QDLV-MSEVYLGRQICNVVACEGSDRV-ER-HETL-NQWRVRMNSSGFDPVHL
GSNA-FKQASMLL--ALFAG------GD--GYRVE---EN--DGCLMLGWHTRPLIATSAWKLLPDSGTG
AGEVEL--------------------
>Brast05G288500.1.p
----------------------------------------------------------------------
--------MGS---LFFPSSSSSSPASSSSYPPSHASDP-------AFLHHRP-----------------
-----------LDHH-------HQYQQ------------------HAAAMVRKRPAPDMD----------
----------------------------------------------------------------------
```

Development • Supplementary information

```
---------NLPPPRRHVTGDLSDVLAAAA------PPAAQQQQLPTAQLPALPAQL---LPPPT-PFQ-
-----HHQQQQQAA----EQHHHMDVAAANPGG--AA----ATTAFVDGIIRDIIGSCH--GGGHGVSVA
QLIHNVREII-------HPCNPALASLLELRLLDLPQP--------------------------------
----------------------------------------------------------------------
----------------------------------------------------------------------
--------PPPHP----SLL-------PPVSPP-----PPPPPPALPPPDKL------------------
-------PAGSPPSPK------PP-----------------------------SAEQA-------AAAAQ
AAAKDRRESQRR--------------------KQRDEEGLHLL-TLLLQCAESVNADNLDDAQAALLEIA
ELATPFGTS-TQRVAAYFAEAVSARLVTSCL-------GLYAPIPPHS-AA----ASITGGRK-IAAAFQ
V-FNG---------------ISPFVKFSHFTANQAIQEAFEREDRVHIIDLDIM-QG--L----QWPGLF
HILA-SRP----GGP----P-RVRLTGLGASMD------ALEATGKRLSDFADTLGLPFEFC-AVA-DKA
GNL-DP------DKLLN-GSGGGGMVAR-------RREAVAVHWLH--HSLY-DVTGN---DANTLGLIQ
R-LAPKVVTMVEQDLS--H---------------SGSFLARFVEAIHYYSALFDSLDAS-----------
-----YG------EDS-PERHVVEQQLLSREIRNVLAVGGPAR--TG-DAKFVGSWRDRLARSGFGPASL
AGSA-AAQAALLL--GMF-P------SD--GYTLV---EE--NGALKLGWKDLCLLTASAWRPMVHTTPS
SSAR----------------------
>Pp3c2_20930V3.1.p
----------------------------------------------------------------------
---------------------------MMPPFGSLLEPSTDSRGGPGSFYVKPELGVDAVKKIGMDTPQS
AFSSYRDAHPPLRKPLQHELSAFGPFAAQGQSSVDASAVN-----VAPILPSVDAHLQLW----------
----------------------------RPTSRQPVQVKHYLQPTVLQSTTNNFLSKKQSTEVSQQF---
----------------------------------------------------NGMFR-------------
-----STNANAMHSK-------------SSDVGSSGLVTGIRDTTSDGFVVVSCKQVKCKQECDIASSSF
SAPPLSRKRS-------HSDLAEEESCDSELVLGIGGRSSVSRGGSEFRNSRAS-SPRKSLKSSDTRLDL
VSADLCLAPPVLAPEYSSSSSQQEVPSSSMLEVQPFGESSKVSHSRGPY---------------------
------------------------------------ASPTESHLSFQKIKSQDASRME--GVTCVSQAES
HPTGSRCCQPVRPLLNL------------------------EAIKRECDAVE------------------
-------RERDNEESL------AQSRTHHE-G---------------------VELGLFPGVFAADTG--
--------------------------------KKSETSGLQLV-NLLHDCAEAVSKGKIETATQKLEELY
SHASLFGDF-MQRVAAFFTEGLAARMVGKDK-------PMYKNLM----------VQSRLDD--YLSAFT
T-LYK---------------VCPYFQFGHFAANQAILEAVEGRSVVHIIDMDLM-QG--L----QWPGFI
QSLS-ERE----DGP----P-KLKITGIGTSCN------SLQDTGRRLASFAETYGVPFEFH-AVV-GEL
EDL-TP------MEL----------GAK-------PGEAVAVNCVMQLHRLL-NNGDK---LHNFIAGLR
S-LHPVMLTLVEQEAN--HN--------------TSSFLGRFVEAVHYYAAVFDSLDSS-----------
-----LP------LAS-EERAKIEQLYFAQQIKNIVACEGVDRI-ER-HETL-DLWQKRMVTAGFRQLPL
SSHA-VTQAKLLL--SLSP-------CG--GYRLS---QQ-PGGSISLNWQDQCLLSASSWVL-------
--------------------------
>Solyc11g012510.1.1
----------------------------------------------------------------------
----------------------------------------------------------------------
----------------------------------------------------------------------
----------------------------------------------------------------------
----------------------------------------------------------------------
--------------------------------------------------------MQTSQRTQMSGSVH
GLYNQQMQQV-------EQYYAPYDVLKNS-CKDNRSSGMQFSLQAQD----------------------
---------EQFFTLDSSPATDYVVN--------DSPPALSVSSNRSPFSPQCSQSY-----MSDLHHSS
DNT--CGS--PLSGCS----GIDDGDLRHVLRELE-NKLLG---PESDTDDSCSCSLN--DMVSK-----
----------------------------------------------------------------------
----------------------------------------------------------------------
---PSSVTRWNR--------------------VLDMAPGLNLK-ELLDACAEAVSDADISTAEALMSALE
QRVSVSGEP-MERLGAYVLEGIRARLLSSGS-------IIYKKLK---------CKEPTGLE--LLSYMQ
V-IFN---------------MCPYYKFAYMSANVVINEAMMNENRIHIIDFQIA-QG--S----QWMFLL
```

Development: doi:10.1242/dev.177543: Supplementary information

```
HYLA-HRP----GGP----P-FVRITGVDDDESAYARGGGLQLVGKRLAEVAKSCGVPFEFH-GAA-LSG
CEV-QL------ENL----------RVK-------HGEALAVNFPYMLHHMPDESVSTINHRDRLLRLVK
S-LSPKIVTLVEQESN--TN--------------TAPFLPRFRETLDYYTAMFESIDAA-----------
-----RP------RDD-KERISAEEHCVARDVVNIIACEGADRV-ER-HELF-GKWRLRLMMAGFTQCQL
SPSV-GETIKHML--KEY--------SP--NYRYA---EG--EGALYLGWKNRALATSSAWR--------
--------------------------
>LOC_Os12g02870.1
----------------------------------------------------------------------
--------MGSSSLLLFPSSSSSAT-------HSSYSPSSSSHAITSLLPPLP-----------------
-----------SDHHLLLYLDH-QEQH-H----------------LAAAMVRKRPASDMD----------
----------------------------------------------------------------------
----------LPPPRRHVTGDLSDVTAAAAGA---------PTLSASAQLPALPTQL-------P-AFH-
-----HTDMDLAAP-------APPAP-QQVAAGEGGP----PSTAWVDGIIRDIIAS----S-GAAVSVA
QLIHNVREII-------RPCNPDLASILELRLRSLL----------------------------------
----------------------------------------------------------------------
--------------------------------------------------------------------NS
DP-APPPPPPSHP----ALLPPDATAPPPPPT-SVAALPPPPPAQ---PDKRRR-------E-----PQC
QEQEPNQP----QSPK------PP-----------------------------TAEETAAAAAAAAAAAA
AAAKERKEEQRR--------------------KQRDEEGLHLL-TLLLQCAESVNADNLDEAHRALLEIA
ELATPFGTS-TQRVAAYFAEAMSARLVSSCL-------GLYAPLPSPS-PA----GARVHGR--VAAAFQ
V-FNG---------------ISPFVKFSHFTANQAIQEAFEREERVHIIDLDIM-QG--L----QWPGLF
HILA-SRP----GGP----P-RVRLTGLGASME------ALEATGKRLSDFADTLGLPFEFC-PVA-DKA
GNL-DP------EKL----------GVT-------RREAVAVHWLR--HSLY-DVTGS---DSNTLWLIQ
R-LAPKVVTMVEQDLS--H---------------SGSFLARFVEAIHYYSALFDSLDAS-----------
-----YS------EDS-PERHVVEQQLLSREIRNVLAVGGPAR--TG-DVKF-GSWREKLAQSGFRVSSL
AGSA-AAQAALLL--GMF-P------SD--GYTLI---EE--NGALKLGWKDLCLLTASAWRPIQAS-GR
--------------------------
>Sevir.9G121800.1.p
----------------------------------------------------------------------
----------------------------------------------------------------------
---MKREYQDA---------GGS--GGDMGSSKDKMMAAV-----AGAGEQDEE-VDELL----------
----------------------------AALGYKVRSSDMADVAQKLEQLEMAMGM---GGAGAAAD---
----------------------------------------------------------------------
--------------------------------------------------------------------DG
FVSHLATDTV-------HYNPSDLSSWVESMLSELNAPPPPLPPA-------------------------
----------------------------------------------------------------------
-------------------------------------PPAPRLASTSSTVTGGAAAG---GVYFD-LPP
AVDSSSSTYALKPIPSPA-AASA------------------DP-STDSAREP------------------
--KRMRTGGGSTSSSS------SSSSSLDG-G---------------------RTRSSVVEAAPPAAQAS
AAASGPAVPVVV--------------------VDTQEAGIRLV-HALLACAEAVQQENFAAAEALVKQIP
MLASSQGGA-MRKVAAYFGEALARRVYRFRPAPD---------------------SSLLDAA--FADLLH
AHFYE---------------SCPYLKFAHFTANQAILEAFAGCRRVHVVDFGIK-QG--M----QWPALL
QALA-LRP----GGP----P-SFRLTGVGPPQP--DETDALQQVGWKLAQFAHTIRVDFQYRGLVA-ATL
ADL-EP------FML----------QPD-GEDTDDEPEVIAVNSVFELHRLL-AQPGA---LEKVLGTVR
A-VRPRIVTVVEQEAN--HN--------------SGSFLDRFTESLHYYSTMFDSLEGAGSGNSA-----
ADASPAP------AGG-TDQV-MSEVYLGRQICNVVACEGTERT-ER-HETL-GQWRNRLGRAGFEPVHL
GSNA-YKQASTLL--ALFAG------GD--GYRVE---EK--DGCLTLGWHTRPLIATSAWRAAAP----
--------------------------
>Brast06G087800.1.p
----------------------------------------------------------------------
----------------------------------------------------------------------
---------------------------MIQGVLSRASAAD-----AAAMKAAKRAASIP-----------
```

Development • Supplementary information

-------------EDE------E-AMVSSR-G---------------------KRLQLHGSGVHQQQQGS
PAAEEGKV------------------------SAAETRGLRLL-SLLLRCAEAVAMDSLPEARDLLHEIA
ELASPFGSS-PERVAAYFGDALCARVLSSYL-------GAYSPLP--LAPA---QQQQQSRR--VASAFQ
A-YNA---------------LSPLVKFSHFTANKAILQALHGEDRVHVVDLDIM-QG--L----QWPGLF
HMLA-SRPCSNSSKP---LL-SLRVTGLGASLD------ALEATGRRLADFAASLGLPFEFR-PVE-GKI
GHVADT------DALL---------LRD-------EGEATVVHWMH--HCLY-DVTGS---DAGTLRVLR
S-LRPKLVTIVEQDLG--H---------------GGDFLGRFVEALHYYSALFDALGAG-----------
-----AGAGAGAEEEE-AQRHAVERQLLGAEIRNIVAVGGPKR--TG-EVRV-ERWGDELRRAGFRPVSL
SGGP-AAQARMLL--GMC-P------WK--GYTLA---EE--DGCLKLGWKDLSLLTASSWEPMSSDGDG
QDDLGAAARQEGHGS-----------
>Solyc10g074680.1.1

-------MAKALPVSGENCTGVNVVGDSSGEKTSKQLFHC-----ENGKMVRKRAASDME----IQTGAG
EEHRYLRRPAMIGGSHSQVGDSRVCGNSNFGHGMNSNLTTTMTMTTQVSNYSTMQLPSSTNLCGVT-SR
GGPGIDTGFSNSTPNLTYTDAITSHHQPQGTQTQNNNSQSP-SVCVFSGLPLFPPDR-------------
-----NRQNSGLLLQQPPAAAAAVVSSPLTTGRIDSMEDSTSATAWIDSIIKDLINS------SAQVSVP
QLIQNVREII-------HPCNPYLASLLEYRLRSLTSNNNGGADQNDPMECWRR----------------
----
-------------------------------KESLPAQLAGLQQAQNNANLLQHNILSLPDSSNNQYLNW
DIALPNSHNAPVAPSHNQHQQLGGNNPTA----TDLSFVTLSPQVQQQQQQQQQESPHSHSQQQAAVDLD
QQQKQQQS----SSSL------SPTSVA-------------------------DNSAKTKTSTPAPPVPI
NTYREKKEEERQ--------------------QKRDEEGLHLL-TLLLQCAEAVSADNLEEANKMLLEVS
ELSTPFGTS-AQRVAAYFSEAMSARLLNSCL-------GIYAALPMTSVPM------LYTQK--MASAFQ
V-FNG---------------ISPFIKFSHFTANQAIQEAFEREDRVHIIDLDIM-QG--L----QWPGLF
HILA-SRP----GGP----P-YVRLTGLGTSMD------ALEATGKRLSDFAERLGLPFEFL-PVA-DKV
GNL-DP------EKL----------NVS-------KREAVAVHWLQ--HSLY-DVTGS---DPNTLSLLQ
R-LAPKVVTVVEQDLS--H---------------AGSFLGRFVEAIHYYSALFDSLGAC-----------
-----YG------EES-EERHVVEQQLLSKEIRNVLAVGGPSR--SG-DAKF-NNWREKLQQSGFRSLSL
AGNA-AAQATLLL--GMF-P------SH--GYTLV---ED--NGTLKLGWKDLCLFTASAWRPNSLHAAP
GSRHFSRPNMD---------------
>Brast06G101600.1.p

---------------------MDLHQLLKYRLTGANVLYEIPTENNLTNSSWPT----------------
--------SPLKLEYNNSPYTPLSTQLECDNLSALSNTPDNQ-SSTETISAQ---PISPLEADSSYIQPG
SHLCENIQVRADPLYA-----RSRHNMQHALREIE-TVLMA---PDADDATTSTKHEFEETKPAQLMRQR
SRTWSHESRQ--
---PLPGVVRPQFASGGYPM
ASYEFRPEKRQR--------------------ELRDDPQIIVK-QLLTKCAEALSEDRTEEFLKLVQEAR
GIVSINGEP-IQRLGAYLLEGLVARHGNSGT-------NIYRALR---------CRKPESKE--LLSYMK

Development • Supplementary information

```
I-LYN---------------ICPYFKFGYMAANGAIAEALRSEDNIHIIDFQIA-QG--T----QWITLI
QALA-ARP----GGP----P-HVRITGIDDPVSEYARGEGLEIVGNMLKGMSKEFNIPLEFT-PLP-VYA
TQV-TK------EML----------EIR-------PGEALAVNFTLQLHHTPDESVDVNNPRDGLLRMVK
G-LSPKVTTLVEQESH--TN--------------TTPFMMRFAETMDYYSAMFESIDAN-----------
-----LP------RDN-KERISVEQHCLAKDIVNIIACEGKDRV-ER-HELL-GKWKSRLTMAGFKPYPL
SSYV-NSVIKKLL--ACY--------SD--KYTLE---EK--DGAMLLGWKKRKLISASAWH--------
--------------------------
>Bradi1g25370.1.p
----------------------------------------------------------------------
----------------------------------------------------------------------
----------------------------------------------------------------------
----------------------------------------------------------------------
----------------------------------------------------------------------
----------------------------------------------------------------------
---------------------MDLHQLLKYRLTGANVLYEIPTENNLTNSSWPA----------------
--------SPLKLEYNNSPYTPLSTQLECDNLSALSNTPDNQ-SSTETISAQ---PISPLEADSSYIQPG
SHLRENIQVRADPLYA-----RSRHNMQHALREIE-TVLMA---PDADDATTSAKHEFEETKPAQLMRQR
SRTWSHESRQ------------------------------------------------------------
-----------------------------------------------------PLPGVVRPQFASGGYPM
ASYEFRPEKRQR--------------------ELRDDPQIIVK-QLLTKCAEALSEDRTEEFLKLVQEAR
GIVSINGEP-IQRLGAYLLEGLVARHGNSGR-------NIYRALR---------CRKPESKE--LLSYMK
I-LYN---------------ICPYFKFGYMAANGAIAEALRSEDNIHIIDFQIA-QG--T----QWITLI
QALA-ARP----GGP----P-HVRITGIDDPVSEYARGEGLEIVGNMLKGMSKEFNIPLEFT-PLP-VYA
TQV-TK------EML----------EIR-------PGEALAVNFTLQLHHTPDESVDVNNPRDGLLRMVK
G-LSPKVTTLVEQESH--TN--------------TTPFMMRFAETMDYYSAMFESIDAN-----------
-----LP------RDN-KERISVEQHCLAKDIVNIIACEGKDRV-ER-HELL-GKWKSRLTMAGFKPYPL
SSYV-NSVIKKLL--ACY--------SD--KYTLE---EK--DGAMLLGWKKRKLISASAWH--------
--------------------------
>Solyc04g014830.1.1
----------------------------------------------------------------------
----------------------------------------------------------------------
----------------------------------------------------------------------
----------------------------------------------------------------------
----------------------------------------------------------------------
--------------------------------------------------------MQASQGPQRSCSVH
KLYNKPMQQV-------QQNYTPCRASDNSNYNDGSNSQAQVSLTTEN----------------------
---------EKFFTVDTFPATDCAIY--------DGDPSVSVSSNRSPFSSQCSQSN-----MFEQRRSY
EKT--AGS--PVSLCS---GVDDSNGKKHELRELN-NKLLR---PESDIDDSCSCSLN--GVVSK-----
----------------------------------------------------------------------
----------------------------------------------------------------------
---HFSLTRRNQ--------------------VLDVASRLDLK-ELLVACAEAVDEADTSTAEVLMDALE
KRVSVYGEP-MQRLSAYMLEGLRARLLSSGS-------NIYKKLK---------CNEPTSSE--LLSYMQ
V-LYH---------------ITPYFKFAYMSANVVISEAMKNENRIHIIDFQIA-QG--S----QWVFLI
HYLA-RRP----GGP----P-FLRITGIDDSQSAHARGGGLQLVGERLASIAKSCGVPFEFH-TAA-LSG
CMV-KL------ENL----------RVR-------HGESLAVNFPYMLHHMPDESVSTMNHRDRLLRLVK
S-LSPKIVALVEQEMN--TN--------------TAPFLPRFRETLDYHKAIFESVDVT-----------
-----RP------RND-MQRIRSEEHCIARDVVNLIACEGADRV-ER-HEVF-GKWRSRLLMAGFTPCPL
SPSV-AEAIKVML--KEY--------SS--NYKLA---ES--QGALYIGWNNRALATSSAWQLPHSLPLG
S-------------------------
>Aco009099.1
----------------------------------------------------------------------
----------------------------------------------------------------------
```

```
----------------------------------------------------------------------
----------------------------------------------------------------------
----------------------------------------------------------------------
----------------------------------------------------------------------
---------------------MDLHQLLKYGLTASDHSCEFPAPRTPAYSDWSA----------------
--------NSIKFNLGNSPHSPLLTQLDSDNLSVYSSSISKQRSPIESLSGNIHSQHSPLEPGSSLRQFQ
IKFSEFTE--PNSQNT-----ITSQNMRHALQELE-TVLMA---PDTDEGATSTNPNTDEHKPAKVARQL
SRTWSHESRR------------------------------------------------------------
-----------------------------------------------------EPIIMVQPQFTSNRYPV
QSFEVRPEKRPR--------------------EMKEDPQNKVK-QLLIKCAEALSENKIEEFDALVQEAH
GMVSISGEP-IQRLGAYLIEGLVARKESSGT-------NIYRALK---------CREPESTE--LLSYMR
T-LYD---------------ICPYFKFGYMAANGAIAEALRSEDRIHIIDFQIA-QG--T----QWITLI
QALA-ARP----GGP----P-HVRITGIDDPLSEYARGDGLQLVGKMLSDMSKKFNIPLEFT-GLP-VYC
PQV-TR------EML----------DIR-------PGEALAVNFTLHLHHTPDESVDVSNPRDGLLRMVK
G-LSPKVTTLVEQESN--TN--------------TTPFLTRFLETLDYYYAMFESIDVT-----------
-----LP------RDS-KERISVEQHCLAKDIVNIIACEGDGRV-ER-HELL-GKWRSRLTMAGFKPYPL
SSYV-NSVIKKLL--GFY--------SD--KYTLV---EK--DGAMLLGWKNRSLISASAWH--------
--------------------------
>GRMZM2G116638_P01
----------------------------------------------------------------------
----------------------------------------------------------------------
----------------------------------------------------------------------
----------------------------------------------------------------------
----------------------------------------------------------------------
--------------------------------------------------------MSARDSNHSYKCSD
DSQMPYYNNS--AP---VGENGRIHMAENSLGHHYSSSDIGSQRINNSNPQVFE----------------
---------AQYCTLESSSANGVYPAQ-------SSTSSHSISPL----------SGSPL--SQHDSHS-
DHT--YSS--PPSASC----LTEVADLLIKQKELE-NSIVG---PGLDISSDCSRSLL--QAHVP-----
----------------------------------------------------------------------
----------------------------------------------------------------------
----VRPDNWR---------------------QLLGINGGDLM-QVVIACGKAVAENDVFATELLISELG
HLVSVSGDP-MQRLGAYMLEGIVARLSSSGS-------MLYKSLK---------CKEPTSSE--LMSYMH
L-LYE---------------ICPFYKFGYMSANGAIAEAIKGENFVHIIDFQIA-QG--S----QWVTLL
QALA-ARP----GGP----P-YIRITGIDDSNSAYARGGGLDIVGRTLCDVANSCGLPFEFN-AVP-AAS
HEV-EL------QHL----------AIR-------HGEIIAVNFAYQLHHVPDESVSTENHRDRIIRMIK
S-INPRVVTLVEQESN--TN--------------TAPFFPRYMETLNYYTAMFESIDVA-----------
-----LP------RDD-RRRMSAEQHCVARDIVNLIACEGAERV-ER-HELF-GKWKSRFAMAGFRPYPL
SSVV-NNTINTLL--HTY--------NS--YYRLE---ER--DGVLYLGWKNRVLVVSSAWC--------
--------------------------
>Bradi4g44093.1.p
----------------------------------------------------------------------
--------MGS---LFFPSSS-----SSSSYPPSHASDS------TAFLHHHL-----------------
-----------LDHH----------QH------------------AAAAMVRKRPAPDMD----------
----------------------------------------------------------------------
---------NLPPPRRHVTGDLSDVLAAANAA----APPQQQQQLPTAQLPALPTQL---LPPPT-PFQ-
-----HQQHQQ-------EQQHHMDVAAANPGG--VA----ATTAFVDGIIRDIIGSCHAGHGGGGVSVA
QLIHNVREII-------HPCNPALASLLELRLRSLLSP--------------------------------
----------------------------------------------------------------------
----------------------------------------------------------------------
---AASDLPPPHP----SLQ------SLPVSPPAAALPPPPPP----PPDKL------------------
-------ISSPPPQPESPKQPPPP-----------------------------TPEQT-------AAAAQ
AAAKSRRETQLR--------------------KQRDEEGLHLL-TLLLQCAESVNADNLDDAQSALLEIA
```

Development • Supplementary information

```
ELATPFGTS-TQRVAAYFAEAVSARLVTSCL-------GLYAPLPPHS-AA----ASITGGRK-IAAAFQ
V-FNG---------------ISPFVKFSHFTANQAIQEAFEREDRVHIIDLDIM-QG--L----QWPGLF
HILA-SRP----GGP----P-RVRLTGLGASMD------ALEATGKRLSDFADTLGLPFEFC-AVA-DKA
GNL-DP------EKLLNGGGGGGGGVGR-------RREAVAVHWLH--HSLY-DVTGN---DANTLGLIQ
R-LAPKVVTMVEQDLS--H---------------SGSFLARFVEAIHYYSALFDSLDAS-----------
-----YG------EDS-PERHVVEQQLLSREIRNVLAVGGPAR--TG-DAKFVGSWRDKLARSGFGPASL
AGSA-AAQAALLL--GMF-P------SD--GYTLV---EE--NGALKLGWKDLCLLTASAWRPMVQTTPS
SSAR----------------------
>Pp3c1_15680V3.1.p
----------------------------------------------------------------------
---------------------------MMPPFGSLLEPPTNLRGGQSSFYVKQEPGVDSVRKLRMDTPQS
AFSSYRDTHPTLRKPLQHELSAFGPFSAQGQSSMDASTSN-----AAPHPPSGDTHTSLR----------
----------------------------KSASRQPIQVHQYLRPAVLQPTTSVSPSKKQCTEKSRQF---
----------------------------------------------------NDVFL-------------
-----SAHTNAMPSK-------------SSDVGSSGLATGIRDTTSDGFVVVSCKQMQCKQECDIASTSF
SAPPLSRKRS-------HSELA-EESCDSELVLGVGGRSSVSREGSEYRNSRAS-SPRKSQKSSDTRLDL
VSADLCLAPPIVAPEYSSSSSQQEAPSSSMLETQPLGESSKVSHSRGTY---------------------
------------------------------------VSPTQSHLSFQKIKSKDVSRAE--GVTCISQAES
HPTGSRCQQPVRPLLNL------------------------EVIKRECDAVE------------------
-------RERDNEESL------AQSRNHHE-G---------------------FELGLFPGVFAADQG--
--------------------------------KKSEASGLQLV-HLLLACADAISKNKIEIATQKLEELY
SHASLFGDS-MQRIAAFFTEALAARIVGKDN-------PAYKNLM----------LQSHLDD--YLSAFT
T-LYK---------------ICPYFQFGHFTANQAILEAVEGYSVVHIIDMDLM-QG--F----QWPGFI
QSLS-ERE----GGP----P-KLKITGVGTSCT------SLQDTGRRLAAFAETYGVPFEFH-AVV-GEL
EDL-SP------MEL----------GAK-------PGEAVAVNCVMQLHRLL-NNGDK---LQNFISGLR
S-IHPVMLTLVEQEAN--HN--------------TSSFMGRFVEALHYYAAVFDSLDSS-----------
-----LP------LAS-EERAKIEQLYFAQQIKNIVACEGADRI-ER-HETL-ELWQKRMKLAGFRQWPL
SSHS-VTQAKLLL--SLSP-------CD--GYCLS---QQ-PGGSISLNWQDRSLLTASTWVL-------
--------------------------
>Sobic.008G023401.1.p
----------------------------------------------------------------------
----------------------------------------------------------------------
-------------------------------------------------MVRKRPAPDVD----------
----------------------------------------------------------------------
----------LPPPRRHVTGDLSDVTAGAGA--AGAPPPHLPPFSASAQLPALPTQLPTAYQFQAHAHQ-
-----HQAAGVDVP-----------AAHAQ-AAEVAAAAAPSTTAWVDGIIRDIIGS----SGGAGVSVA
QLIHNVREII-------HPCNPGLASLLELRLRSLLA---------------------------------
----------------------------------------------------------------------
--------------------------------------------------------------------PA
DP-APLHPP---------LL---H--AAPHVAALPPAPPPPPP----LHSAEKH-------C---PPPPQ
QDQEPNNPPAPSQSPK------AP-----------------------------TAEET----------AA
AAAKERKEEPRR--------------------RQRDEEGLHLL-TLLLQCAEAVNADNLDDAHRCLLEIA
ELATPFGTS-THRVAAYFAEAMSARLVTSCL-------GIYAPLPPGN-PV----AARLHGRGVAAAAFQ
V-FNG---------------ISPFVKFSHFTANQAIQEAFEREDRVHIIDLDIM-QG--L----QWPGLF
HILA-SRP----GGP----P-RVRLTGLGASMD------ALQATGKRLSDFADTLGLPFEFC-AVA-EKA
GNV-DP------EKL----------GVT-------RREAVAVHWLH--HSLY-DVTGS---DSNTLWLIQ
R-LAPKVVTMVEQDLS--Q---------------SGSFLARFVEAIHYYSALFDSLDAS-----------
-----YG------EDS-PERHVVEQQLLSREIRNVLAVGGPAR--TG-DVKFVGSWREKLAQSGFRAASL
AGGA-AAQASLLL--GMF-P------SD--GYTLV---EE--NGALKLGWKDLCLLTASAWRPIHQAPCR
--------------------------
>Sobic.005G123000.1.p
----------------------------------------------------------------------
```


-------------------------------------MT-------------------------------
-------TLQHQMAKS------DQWAAAAD-S---------------------SLHSMLGS---------
----VIQAEA----------------------EQEQDSGLQLV-HLLLACADFVSKGDQPSALRHLHLLR
RVASPLGDS-MQRVASYFADALAARLTLSSNPSSCSSSGGVATPRGGAGAGVAPYTFPPSPD--TLKIYQ
I-LYQ---------------ACPYVKFAHFTANQAIFEAFHGEDRVHVVDLDIL-QG--Y----QWPAFL
QALA-ARP----GGP----P-TLRLTGVGHPSA------AVRETGRHLASLAASLRVPFEFHAAVA-DRL
ERL-RP------GAL----------QRR-------VGEALAVNAVNRLHRVP---GVH---LGPLLSMIR
D-QAPKIMTLVEQEAG--HN--------------GPYFLGRFLEALHYYSAIFDSLDAT-----------
-----FP------ADS-APRMKVEQCLLAPEIRNVVACEGAERV-AR-HERL-DRWRRLMEGRGFEPVPL
SPAA-VGQSQVLL--GLYGA------GD--GYRLT---ED--KGCLLLGWQDRAIIAASAWRC-------
----
>Pp3c22_7230V3.1.p

-----------------------------------MAYQY-----YPGSTRYEATGGALV----------
----------------------------DGQFRHANFMQPSDLVQHLEQLHSVLGTVSQDSPNIPAH---
----

HTLDAGAQTS-------NNRTSDLAGWIDGMIDELSFNN-------------------------------
----

---AGTMAAPQQRS-LTEDSLHQNELEASSSH
DSSLDTGSSRLPTLHY------QNTPAVGN-N---------------------FLATPQNDASQLNANRA
TGAVLEQQPSPM--------------------GEDEDNGVRLV-HSLLACAESIQRGNLNLAEQTLRRIQ
LLSLPPG-P-MGKVATHFIDALTCRIYGVAFSSGNN------------------VGSNQSDS--LSELLH
FHFYE---------------TCPYLKFAHFTANQAILEAFAGQKQVHVIDFNLM-HG--L----QWPALI
QALA-LRP----GGP----P-RLRLTGIGPPQS--GGSDVLQEIGMKLAQLAETVKVEFEFRGVVA-VKL
DDI-KP------WML----------QIC-------HGEAVAVNSVFQLHKLL-YSAGSVIPIDEVLRSAR
A-LKPKIFTIVEHEAN--HN--------------QPSFLGRFTEALHYYSTMFDSLEAC-----------
----SLP------SDS-SEQV-LAEMYLGREINNIVACEDAARV-ER-HENL-VQWQMRMLKAGYRPIQL
GLNA-FKQASMLL--TMFS-------GD--GYRVE---EK--LGCLTLGWHTRPLISASAWQCA------
----
>GRMZM2G144744_P01

---MKREYQDA---------GGS--GGDMGSSKDKMMAAA-----AGAGEQEEEDVDELL----------
----------------------------AALGYKVRSSDMADVAQKLEQLEMAMGMGGVGGAGATAD---
----
--DG
FVSHLATDTV-------HYNPSDLSSWVESMLSELNAPPAPLPPA-------------------------
----
--------------------------------------TPAPRLASTSSTVTSGAAAG---AGYFD-LPP
AVDSSSSTYALKPIPSPVAAPSA------------------DP-STDSAREP------------------
--KRMRTGGGSTSSSS------SSSSSMDG-G---------------------RTRSSVVEAAPPATQAS

```
AAANGPAVPVVV--------------------VDTQEAGIRLV-HALLACAEAVQQENFSAAEALVKQIP
MLASSQGGA-MRKVAAYFGEALARRVYRFRPPPD---------------------SSLLDAA--FADLLH
AHFYE---------------SCPYLKFAHFTANQAILEAFAGCRRVHVVDFGIK-QG--M----QWPALL
QALA-LRP----GGP----P-SFRLTGVGPPQP--DETDALQQVGWKLAQFAHTIRVDFQYRGLVA-ATL
ADL-EP------FML----------QPE-GDDTDDEPEVIAVNSVFELHRLL-AQPGA---LEKVLGTVR
A-VRPRIVTVVEQEAN--HN--------------SGTFLDRFTESLHYYSTMFDSLEGAGAGSGQS----
TDASPAA------AGG-TDQV-MSEVYLGRQICNVVACEGAERT-ER-HETL-GQWRSRLGGSGFAPVHL
GSNA-YKQASTLL--ALFAG------GD--GYRVE---EK--DGCLTLGWHTRPLIATSAWRVAAAAAP-
--------------------------
>Seita.2G343700.1.p
----------------------------------------------------------------------
----------------------------------------------------------------------
----------------------------------------------------------------------
----------------------------------------------------------------------
----------------------------------------------------------------------
----------------------------------------------------------------------
---------------------MDLHQLLKYRLTGANVFYEFPTENNLANNPWPG----------------
--------TPLKSEFSNSPYTPLSAQLECDNLSAISNTPDNQ-SSTETISAQ---PISPLEVDSSNRQAG
I-LWENTQVRPDHLYT-----TSRHNMQHALRKIE-TVLMA---PDADDAATSTKHEFEEHKPAPLMRQR
SRTWSHELRQ------------------------------------------------------------
-----------------------------------------------------PSPGVVRTQFAS-GYPT
ASYEFRPEKRQR--------------------ELREDPQSMVK-QLLTKCAEALSEERIEEFLKLVQQAR
GVVSITGEP-IQRLGAYLLEGLVARHGNSGT-------NIYRALK---------CREPESNE--LLSYMK
I-LYN---------------ICPYFKFGYMAANGAIAEALRNEDKIHIVDFQIA-QG--T----QWITLI
QALA-ARP----GGP----P-HVRITGIDDPVSEYARGEGLDLVGKMLKSMSEEFRIPLEFT-PLPGVYA
TQV-TK------EML----------DIR-------PGEALAVNFTLQLHHTPDESVDVNNPRDGLLRMVK
G-LSPKVTTLVEQESH--TN--------------TTPFLMRFSETMDYYSAMFESIDAN-----------
-----LP------RDS-KERINVEQHCLAKDIVNIIACEGKDRV-ER-HELL-GKWKSRLTMAGFKPYPL
SSYV-NSVIRKLL--ACY--------SD--KYTLE---EK--DGAMLLGWKSRKLISASAWH--------
--------------------------
>Bradi1g11090.1.p
----------------------------------------------------------------------
----------------------------------------------------------------------
---MKREYQDG---------GGSRGGGDMGSSKDKMMVAA-----PA---EEED-MDELL----------
----------------------------AALGYKVRASDMADVAQKLEQLEMAMGMGGV-GAGAAPD---
----------------------------------------------------------------------
--------------------------------------------------------------------DS
FVAHLATDTV-------HSNPSDLSSWVESMLSELNAPPPPLPPA-------------------------
----------------------------------------------------------------------
--------------------------------------PQAPRLSSNSSTVTGGGGSG---GGYFDGLPP
SVDSSSSTYALRPIPSPV-VTPA------------------EP-SADPAREP------------------
--KRMRTGGGSTSSSS------SSSSSLGG-G---------------------GTMSSVVEAAPPAAPAS
AAANAPALPVVV--------------------VDTQEAGIRLV-HALLACAEAVQQENLSAAEALVKQIP
LLAASQGGA-MRKVAAYFGEALARRVFRFRPQPD---------------------SSLLDAA--FADLLH
AHFYE---------------SCPYLKFAHFTANQAILEAFAGCRRVHVVDFGIK-QG--M----QWPALL
QALA-LRP----GGP----P-SFRLTGVGPPQP--DETDALQQVGWKLAQFAHTIRVDFQYRGLVA-ATL
ADL-EP------FML----------QPEGEEDPNEEPEVIAVNSVFEMHRLL-SQPGA---LEKVLGTVR
A-VRPRIVTVVEQEAN--HN--------------SGSFLDRFTESLHYYSTMFDSLEGAGSGQS------
EISPGAA------AGA-TDQV-MSEVYLGRQICNVVACEGPERT-ER-HETL-GQWRGRLGQAGFETVHL
GSNA-YKQASTLL--ALFAG------GD--GYKVE---EK--DGCLTLGWHTRPLIATSAWRMAAP----
--------------------------
>AT3G03450.1
```

Development • Supplementary information

```
----------------------------------------------------------------------
----------------------------------------------------------------------
---MKRGYGETWDPPPKPLPASRSGEGPSMADKKKADDDN-----NNSNMD-----DELL----------
----------------------------AVLGYKVRSSEMAEVAQKLEQLEMVLSN--------------
----------------------------------------------------------------------
--------------------------------------------------------------------DD
VGSTVLNDSV-------HYNPSDLSNWVESMLSELNNP--------------------------------
----------------------------------------------------------------------
---------------------------------------ASSDLDTTRSCV-------------------
----DRSEYDLRAIPGLS-AFPK------------------EEEVFDEEASS------------------
--KRIRLGSWCESSDE------STR---------------------------------------------
--------SVVL--------------------VDSQETGVRLV-HALVACAEAIHQENLNLADALVKRVG
TLAGSQAGA-MGKVATYFAQALARRIYRDYTAET-------------------DVCAAVNPS--FEEVLE
MHFYE---------------SCPYLKFAHFTANQAILEAVTTARRVHVIDLGLN-QG--M----QWPALM
QALA-LRP----GGP----P-SFRLTGIGPPQT--ENSDSLQQLGWKLAQFAQNMGVEFEFKGLAA-ESL
SDL-EP------EMF----------ETR------PESETLVVNSVFELHRLL-ARSGS---IEKLLNTVK
A-IKPSIVTVVEQEAN--HN--------------GIVFLDRFNEALHYYSSLFDSLEDS-----------
-----YS------LPS-QDRV-MSEVYLGRQILNVVAAEGSDRV-ER-HETA-AQWRIRMKSAGFDPIHL
GSSA-FKQASMLL--SLYAT------GD--GYRVE---EN--DGCLMIGWQTRPLITTSAWKLA------
--------------------------
>Sobic.009G177100.1.p
----------------------------------------------------------------------
----------------------------------------------------------------------
----------------------------------------------------------------------
----------------------------------------------------------------------
----------------------------------------------------------------------
----------------------------------------------------------------------
----------------------------------------------------------------------
----------------------------------------------------------------------
----------------------------------------------------------------------
------------MQDSLSLM-QFHD--PYLYSGSAGSANLPL-SHPFSPHHHQGFVDN------------
CTGK-DVAEFVD-PAT------TTAAEVSSDE---------------------QELAVSKEVSEGGGGGA
VVVEVEERNDAT----------------TARGGELEAHGVRMI-ALLMESAVAVSVGNLADANSMLLELA
QMASPYASSCGERLVAYFTKALAARLMSSWV-------GICAPLA------------PPCAA--VHAAFR
A-FYN---------------VSPFARFAYLACNQAILEAFHGKRLVHIVDLDVV-PGGAL----QWLSLL
PALA-ARP----GGP----P-VLRVTGFGMSRS------ALHDTGNQLAGLASKLNMPFEFY-AIA-KRP
GDV-VVGAAVA-DMP----------SRR-------PGEALAVHWLR--HALY-DAAGD---DGATMQLVQ
W-LEPKVLTLVEQERAGAAPGDVGGGGGD-----HGHFLDRFVSALHHYSALFDSLGAS-----------
-----RPS-----ELD-ASRHLVEQGVLGREIGNVLAVGGPSR--SG-RGKF-GCWQEELARHGFLRAGG
-----VGRAQLVA--GACPA------GL--GYTVA---DD-HHGTVRLGWKGTPLYAVSTWAWCPSPHGQ
AHR-----------------------
>Sevir.3G205300.1.p
----------------------------------------------------------------------
----------------------------------------------------------------------
----------------------------------------------------------------------
----------------------------------------------------------------------
----------------------------------------------------------------------
----------------------------------------------------------------------
----------------------------------------------------------------------
----------------------------------------------------------------------
----------------------------------------------------------------------
----MSTGRGSDMQDSLSLM-QFHD--PYLYGGGAGGANLPLSSHSFLPHHHD-FVHA---------DDR
```

Development • Supplementary information

```
CPGK-DVPEFVD-QA---------AAEVSC-E---------------------QELAVSKEVSEGGGDGA
--VEEQNNGAAT----------------TAARGEEEAHGVRMI-ALLMECAVAVSVGNLADANGMLLELA
QMASPYAPSCGERLVAYFTKAMAARLMSSWV-------GICAPLA------------PPFAA--VHAAFR
A-FYN---------------VSPLARFAYLACNQGILEAFHGKCLVHIVDLDVV-PGGAL----QWLSLL
PALA-ARP----GGP----P-VLRVTGFGMSAS------ALHDTGNQLAGLASKLGVPFEFY-AVA-RRP
GD--DADAAAA-AVP----------SRR-------PGEALAVHWLR--HALY-DAAGD---DGATMRLVR
W-LEPKVLTLVEQERG--APGDGGGGAGN----ERGHFLDRFVSALHHYSALFDSLGAS-----------
-----RPA-----DED-ASRHLVEHGVLGREIGNVLAVGGPSR--SG-RDKF-GCWQTELARHGFLRAGG
-----GGRAQLVA--GACPA------GL--GYTVA---DD-QDGTVRLGWKGTPLYAVSTWTWCPSPHAQ
R-------------------------
>Seita.2G356700.1.p
----------------------------------------------------------------------
----------------------------------------------------------------------
---------------------------MLQGVLSRAPATDAAA--AAAMK-AKRAPVSPD----------
----------------------------------------------------------------------
----------------------------------------------------------------------
----------------------------------------------------------------------
----------------------------------------------------------------------
----------------------------------------------------------------------
----------------------------------------------------------------------
----------------------------------------------------------------------
-------------EKG------DGDGCPAR-G---------------------KRQQLLGLGP------A
AAAE----------------------------EGPETRGLRLL-SLLLRCAEAVAMDQLTEARELLPEIG
ELASPFGSS-PERVAAYFGDALCARVLSSYL-------GAYSPLA--LRPL----AAAQSRR--VAGAFQ
S-YNA---------------LSPLVKFSHFTANQAILQALDGEDRLHVIDLDIM-QG--L----QWPGLF
HILA-SRP----RKP----R-SIRITGLGASLD------VLEATGRRLADFATSLGLPFEFH-PIE-GKI
GHVADA------AALLGPRHH----HQQ-------QDEATVVHWMH--HCLY-DVTGS---DVGTVRLLR
T-LRPKLITIVEQDLG--H---------------SGDFLGRFVEALHYYSALFDALGDG-----------
-----AGA---AEEEA-AERHAVERQLLGAEIRNIVAVGGPKR--TG-EVRV-ERWSDELRRAGFRPVSL
AGSP-ATQARLLL--GMY-P------WK--GYTLV---EE--DACLKLGWKDLSLLTASAWEPADDDTVA
AAPTPRHGSQET--------------
>Brast08G081600.1.p
----------------------------------------------------------------------
----------------------------------------------------------------------
----------------------------------------------------------------------
----------------------------------------------------------------------
----------------------------------------------------------------------
----------------------------------------------------------------------
----------------------------------------------------------------------
----------------------------------------------------------------------
----------------------------------------------------------------------
--------------------MQFHDHHQHQYLYSSSSGS----AGPFLPHHHAAFIQADEGRCAGSGKDV
AQGE-SLVEILD-EAP------AG----AV-EE--------------------RKGAVDHEAAEEE----
-----EEEEEEE--------------------ESAEAHGVRMI-ALLMECAVAMSVGNLADANGALLELS
QTASPYAASCGERLVAHFARALAARLMSSWV-------GVLAPLPPQ-------HAAAGISG--IHAAFR
A-MYN---------------VSPFARAAYLACNQAVLDAFRGQRVVHVVDMDVV-PGGAL----QWLSLL
PALA-ARP----GGP----PALLRVTGFGASPG------PLHDAGSQLAGLAGKLGLPFEFY-AVP-KRP
GDA-GLP-----LLP----------GKR-------PGEAVAVHWLR--HALY-DAAGD---EAAAMRLAR
W-LEPRVMTVVDQAQAAQPERPSSSSSPS-----AGGFLDRFVCALHHYSAVFDALGAA-----------
-----RPA-----GGD-ASRHLAESGVLGREIGNVLAVAGPSRS-GRRREAG-AGWQAELARHGFLRARW
GAG--GARARMLA--GACPA------GL--GYTVA---DD-HDGTVRLGWKGTPLYAVSTWTWCATTTTH
GHVRGFD-------------------
```

```
>Pp3c19_18560V3.1.p
MALVCPNPRERLGLVTKEPTGVGSRTHYNVGFPGYRDQLRMTDNLRDPFHSGPETGGRDPQDAERRGELQ
EHRVHLPDLDFGVGAKWNCTGQISSWSSYDSTSQSFPSTSAAGWVVSPLGSNNSPFPSDGSFSDHLSSGL
SNTSFPGALPASLQVQSGGSAGGFFSGSTQTASCNGNQLY-----QNPPSLMNAAPRDSW----------
----------------------------GKI--EDAPSPHHQLSCQYDMEQHGNYGYGVQPAREAVK---
----------------------------------------------------------------------
--------------------------------DHFSGMDEDSVAAWVDGMIMEMMEA------MPGVPIE
QILTNLSEVL-------APSNSQLERIIGSRVQSLYGGAGLRVQTFLHTGPSRF-GKRTR-------EGQ
IGWRNAKSSQNEARAY------------------------------------------------------
-------------------------------------QDTPSHHHVRPDNFMEQNAV---ASLARDELHY
P------RPQKRTIDNLQLSLEPADERSLRHLHTAQQPMPEDQYSHQVLRSRDQTTKPVQHQHYFQPHLQ
HQQQKSGEIQNDFSPP------SNAAQYPI-L---------------------NTRGFNDAVSLPQNVPQ
STPRNAQPEPP---------------------NATDEEGLQLL-ALLLQCAEAVSSDDFDQANSILPQLS
ELATPYGTS-VQRVVAYFAEGMASRLVTYCL-------GICPPLSSK--------QLVSNQS--FLSAMQ
V-FNE---------------ICPFVKFSHFTANQAIFDAFEGMFNVHVIDIDIM-HG--L----QWPPLF
QLLA-SRP----GGP----P-HVHITGLGTSIE------TLEAT--------------------------
-------------------------------------DAVAVHWMH--HSLY-DVTGS---DLNTLNLIE
K-LNPKVITLVEQDLR--H---------------GGTFLSRFVEALHYYSALFDSLGAS-----------
-----YK------ADS-PERHMVEQQLLSCEIKNILAFGGPAR--TG-EAKF-DQWRDELGKR-FKPVSL
SGKA-AHQAALLL-QGLF-P------CE--GYTLL---EH--RGTLKLGWKDLYLFTASAWTNE------
--------------------------
>Sevir.2G367700.1.p
----------------------------------------------------------------------
----------------------------------------------------------------------
---------------------------MLQGVLSRAPATDAAA--AAAMK-AKRAPVSPD----------
----------------------------------------------------------------------
----------------------------------------------------------------------
----------------------------------------------------------------------
----------------------------------------------------------------------
----------------------------------------------------------------------
----------------------------------------------------------------------
----------------------------------------------------------------------
-------------EKG------DGDGCPAR-G---------------------KRQQLLGLGP------A
AAAE----------------------------EGPETRGLRLL-SLLLRCAEAVAMDQLTEARELLPEIG
ELASPFGSS-PERVAAYFGDALCARVLSSYL-------GAYSPLA--LRPL----AAAQSRR--VAGAFQ
S-YNA---------------LSPLVKFSHFTANQAILQALDGEDRLHVIDLDIM-QG--L----QWPGLF
HILA-SRP----RKP----R-SIRITGLGASLD------VLEATGRRLADFATSLGLPFEFH-PIE-GKI
GHVADA------AALLGPRHH----HHQ-------QDEATVVHWMH--HCLY-DVTGS---DVGTVRLLR
T-LRPKLITIVEQDLG--H---------------SGDFLGRFVEALHYYSALFDALGDG-----------
-----AGA---AEEEA-AERHAVERQLLGAEIRNIVAVGGPKR--TG-EVRV-ERWSDELRRAGFRPVSL
AGSP-ATQARLLL--GMY-P------WK--GYTLV---EE--DACLKLGWKDLSLLTASAWEPADDDTVA
AAPTPRHGSQET--------------
>GRMZM2G037286_P01
----------------------------------------------------------------------
----------------------------------------------------------------------
----------------------------------------------------------------------
----------------------------------------------------------------------
----------------------------------------------------------------------
----------------------------------------------------------------------
----------------------------------------------------------------------
----------------------------------------------------------------------
----------------------------------------------------------------------
```

```
------------MQDSLSLM-QFHD--PYLYGGSAGSASLPL-PHPFLPQHHQGFVHG------------
CTSK-GVAEFVDPEA---------TAEVGG-E---------------------QGFAVSKEVSEGGGDGA
--VEVEERNDAA----------------TTARGEEEAHGVRMI-ALLMESAVAVSVGNLADANGMLLELA
QMASPYASSCGERLVAYFTKALAARLMSSWV-------GICAPLA------------PPCAA--VHAAFR
A-FYN---------------VSPLARFAYLACNQAILEAFHGKRLVHIVDLDVV-PGGAL----QWLSLL
PALA-ARP----GGP----P-VLRVTGFGISRS------ALHDTGNQLAGLASKLSMPFEFY-AIA-RRP
GDA-VVGAAAA-DMP----------SRR-------PGEALAVHWLR--HALY-DAAGD---DAATMQLVR
W-LEPKVLTLVEQERG--SPGDGGAGAGAAGHDEHGHFLDRFVSALHHYSAMFDSLGAS-----------
-----RPS-----DED-ASRHLVEQGVLGREIGNVLAVGGPSR--SG-RGKF-GCWQAELDRLGFLRAGG
-----GGRAQLVA--GACPA------GL--GYTVA---DD-QDGTVRLGWKGTPLYAVSTWAWCPSPHAQ
R-------------------------
>Sobic.001G120900.1.p
----------------------------------------------------------------------
----------------------------------------------------------------------
---MKREYQDA---------GGS--GGDMGSSKDKMMVAA-----AGAGEQEEE-LDEML----------
----------------------------ASLGYKVRSSDMADVAQKLEQLEMAMGMGGVGGAGATAD---
----------------------------------------------------------------------
--------------------------------------------------------------------DG
FISHLATDTV-------HYNPSDLSSWLESMLSELNAPPPPLPPA-------------------------
----------------------------------------------------------------------
------------------------------------TTPPAPRLASTSSTVTSGAAAG---AGYFD-LPP
AVDSSSSTYALKPIPSPVAVASA------------------DPSSTDSTREP------------------
--KRMRTGGGSTSSSS------SSSSSMDG-G---------------------RTRSSVVEAAPPATQAP
AAANGPAVPVVV--------------------MDTQEAGIRLV-HALLACAEAVQQENFSAADALVKQIP
MLASSQGGA-MRKVAAYFGEALARRVYRFRPTPD---------------------TSLLDAA--VADFLH
AHFYE---------------SCPYLKFAHFTANQAILEAFAGCRRVHVVDFGIK-QG--L----QWPALL
QALA-LRP----GGP----P-SFRLTGVGPPQH--DETDALQQVGWKLAQFAHTIRVDFQYRGLVA-ATL
ADL-EP------FML----------QPE-GDDKDEEPEVIAVNSVFELHRLL-AQPGA---LEKVLGTVR
A-VRPRIVTVVEQEAN--HN--------------SGTFLDRFTESLHYYSTMFDSLEGAGSGQS------
TDASPAA------AGG-TDQV-MSEVYLGRQICNVVACEGAERT-ER-HETL-SQWRGRLVGSGFEPVHL
GSNA-YKQASTLL--ALFNG------GD--GYRVE---EK--DGCLTLGWHTRPLIATSAWRLAAP----
--------------------------
>Pp3c6_16410V3.1.p
----------------------------------------------------------------------
----------------------------------------------------------------------
----------------------------------------------------------------------
----------------------------------------------------------------------
----------------------------------------------------------------------
-------------------------------------------------------------MQLFVPPLR
MRVHSHNFSR-------PQLSLRFPGGLHEMLGSVGSNASASSEPVPEVSSPHRTYGYVD----------
----------------------------------------------------------------------
----------------------------------------------------------------------
----------------------------------------------------------------------
----------------------------------------------------------------------
--------------------------------CSNSLGGLMLLRDILVDTAQYISQCDWERARPLLQVLR
RQVSSTGDS-SERVASCFFEALATRFSRVSGT------EINELLSSP-------TQEPSSEE--ILSAFL
A-LNQ---------------VTPFMRFAHLTANQALLEALTGEDFVHIVDLDIG-HG--V----QWPPFM
QALA-DIR----GEEGHTIQ-HLRITGVGKDRE------MLDRTGTRLAEFAQSIQLPFEFT-PLV-QAP
ENL-IP------SMF----------GLR-------IGEAVAFNCMLQLHQLLAKGSEK---LTSFLYMLE
S-LTPRVVTLAELEAS--HN--------------QPHFLDRFAEALNHYSTLFDSLDAT-----------
-----LP------PTS-PERIRVEQTWYKMEIINIVACDGTERT-VR-HQRC-EQWRRFFERAGFQLLPT
SRFA-TSQARLLL--RLHYP------CD--GYRLV---EDVEDGCLLLGWQDRPLFCVSSWHPSNM----
```

Development • Supplementary information

```
--------------------------
>Solyc03g110950.1.1
----------------------------------------------------------------------
----------------------------------------------------------------------
----------------------------------------------------------------------
-----------------------------------MIHDMANVFLSLEPCNGDQIGYDPMENSLYLT---
----------------------------------------------------------------------
----------------------------------------------------------------------
----------------------------------------------------------------------
----------------------------------------------------------------------
---------HHKELSYSLNPYTSVLKRNAPTNNMIISSLSNDSASFKRLRRTPSLGES--FGSNTTFYST
ESSSTGGSL-------------------------------------------------------------
-------PRIGSSNSV------NSLSLQPG-I---------------------HFRDHVWALNQRYLAAE
AFEEAAADIINQ--------------------EEENGEGMKLV-QLLITCAEAVACRDKSRASVLLSELR
ASALVFGTS-FQRVASCFMQGLSDRLALVQPL------GTVGYVATPAMN----KTDIALEK--KEEALR
L-LYE---------------ICPHIQFGHFVANCLILEAFEGESFIHVVDLGMS-LG--LPHGHQWRRLV
QSLV-NRP----GQP----PRRLRITAVGQNIE------KLQIIGDELEDYARSLGINLEFS-AVE-SNL
ENL-KP------KDI----------KVY-------DGEVLVVNSILQLHCVVKESRGA---LNSVLQVVH
E-LSPKILVLVEQDSS--HN--------------GPFFLGRFMEALHYYSAIFDSLDVM-----------
-----LP------KYD-TRRAKIEQFYFAEEIKNIVSCEGPARV-ER-HERV-DQWRRRMSRAGFQAAPI
KM---VSQAKQWL--AKVNG------HE--GFTIT---EE--KGCLVLGWKSKPIVAASCWKC-------
--------------------------
>AT5G41920.1
----------------------------------------------------------------------
----------------------------------------------------------------------
-------------------------------------------------MTTKRIDRDL-----------
----------------------------------------------------------------------
----------------------------------------------------------------------
----------------------------------------------------------------------
----------------------------------------------------------------------
----------------------------------------------------------------------
----------------------------------------------------------------------
----------------------------------------------------------------------
------------PSSD------DPSSAKRR----------------------------------------
----IEFPEETL--------------------ENDGAAAIKLL-SLLLQCAEYVATDHLREASTLLSEIS
EICSPFGSS-PERVVAYFAQALQTRVISSYLS------GACSPLS--EKPL----TVVQSQK--IFSALQ
T-YNS---------------VSPLIKFSHFTANQAIFQALDGEDSVHIIDLDVM-QG--L----QWPALF
HILA-SRP----RKL----R-SIRITGFGSSSD------LLASTGRRLADFASSLNLPFEFH-PIE-GII
GNLIDP------SQL----------ATR-------QGEAVVVHWMQ--HRLY-DVTGN---NLETLEILR
R-LKPNLITVVEQELSYDD---------------GGSFLGRFVEALHYYSALFDALGDG-----------
-----LG------EES-GERFTVEQIVLGTEIRNIVAHGGGRR-------KR-MKWKEELSRVGFRPVSL
RGNP-ATQAGLLL--GML-P------WN--GYTLV---EE--NGTLRLGWKDLSLLTASAWKSQPFD---
--------------------------
>AT2G04890.1
----------------------------------------------------------------------
----------------------------------------------------------------------
----------------------------------------------------------------------
----------------------------------------------------------------------
----------------------------------------------------------------------
----------------------------------------------------------------------
----------------------------------------------------------------------
----------------------------------------------------------------------
```

```
------------------------------MDNVRGSIMLQ---PLPEIAESIDDAIC--HELS------
----------------------------------------------------------------------
----------------------------------------------------------------------
-MWPDDAKDLLL--------------------IVEAISRGDLK-LVLVACAKAVSENNLLMARWCMGELR
GMVSISGEP-IQRLGAYMLEGLVARLAASGS-------SIYKSLQ---------SREPESYE--FLSYVY
V-LHE---------------VCPYFKFGYMSANGAIAEAMKDEERIHIIDFQIG-QG--S----QWIALI
QAFA-ARP----GGA----P-NIRITGVGD-------GSVLVTVKKRLEKLAKKFDVPFRFN-AVS-RPS
CEV-EV------ENL----------DVR-------DGEALGVNFAYMLHHLPDESVSMENHRDRLLRMVK
S-LSPKVVTLVEQECN--TN--------------TSPFLPRFLETLSYYTAMFESIDVM-----------
-----LP------RNH-KERINIEQHCMARDVVNIIACEGAERI-ER-HELL-GKWKSRFSMAGFEPYPL
SSII-SATIRALL--RDY--------SN--GYAIE---ER--DGALYLGWMDRILVSSCAWK--------
--------------------------
>Pp3c2_30770V3.1.p
----------------------------------------------------------------------
----------------------------------------------------------------------
----------------------------------------------------------------------
----------------------------------------------------------------------
----------------------------------------------------------------------
-----------MSVQYEPELGSMVLTPGFPPGKEREYFSDTANSQQAPNYFGARTSYANDHRQNAYGLKI
KSHGSPISPLS------PQDSSHAASDNGQRMSGGWNSASYQSESSSHSDTSLE--------------GH
VKQEEADYYGRQYGHIEQSEGSLAYH--------NSPSSVLKPMPYPGAIAQGYS-------MPNYQQDG
RHVPDGQYAQSQSNCA-----QRNPEMVHMLQVLE-NALLD-----DDDDDD---------HRAPEGNWA
DTIEKFLAADNSAIKPSPVTSRTTQ---------------------------------------------
-----------------------------------------------------PDYGKQQCNENAINFTG
AVTARVEELALQ--------------------KLVVATRSRSE-QLLVACAEAVSNNDMPLANVLIAQLN
QEVSIHGDP-MQRLAAYMVEGLVARVAASGK-------SIYTSLK---------CKEPPTRD--LLSAMQ
I-LYE---------------VCPYFKFGYMAANGAIAEAFQNESRVHIIDFQIA-QG--T----QWTTLI
RALA-ARP----GGP----P-HVRITGIDDPMPGPTPNVGVEMVGKRLANLAEAVGVPFVFH-PVA-KKG
TEI-EA------WML----------ERQ-------QGEALAVNFALQLHHMPDESVCTSNPRDRMLHMIK
G-LNPKVMTLVEQESN--TN--------------TAPFFPRFLEALSYYSAIFESLDIT-----------
-----LA------RES-KERVNVEQQCLARDIVNIIACEGIDRV-ER-HEMM-GKWRARLTMAGFRPYPL
SQTV-NNTIKTLL--ESY--------SD--KYRLK---EE--GGALFLGWKNRPLIVSSAWH--------
--------------------------
>GRMZM2G024973_P01
----------------------------------------------------------------------
----------------------------------------------------------------------
---MKREYQNA---------GGN--DGYRGSSKDKSMAAA-----AGAGEQEEE-VDELL----------
----------------------------AALGYKVRSSDMADVAQKLEQLEMAMGM---GGACPTAD---
----------------------------------------------------------------------
--------------------------------------------------------------------DG
FVSHLATDTV-------HYNPSDLSSWVESMLSELNAPPPPLPPA-------------------------
----------------------------------------------------------------------
--------------------------------------TPAPRLASTSSTVTSGAAAG---AGYFD-LPP
AVDSSSSTYALKPIPSPVAAASA------------------DP-SPDSAREP------------------
--KRMRTGGGSTSSSS------SSSSSMDG-G---------------------RTRSSVVEAAPPATQ--
-AANGPAVPVVV--------------------VDTQEAGIRLV-HALLACAEAVQQENFSAADALVKQIP
VLASSQGGA-MRKVAAYFGEALARRVYRLRPAPD---------------------GSLLDAA--FADLLH
AHFYE---------------SCPYLKFAHFTANQAILEAFAGCRRVHVVDFGIK-QG--M----QWPALL
QALA-LRP----GGP----P-SFRLTGVGPPQP--DETDALQQVGWKLAQFAHTIRVDFQYRGLVA-ATL
ADL-EP------FML----------RPEGGGDTDDEPEVIAVNSVCELHRLL-AQPGT---LDKVLGTVR
A-VRPRIVTVVEQEAN--HN--------------SGTFLDRFTESLHYYSTMFDSLEGAGSGSGSGSGSG
QPTDASP------PAG-TDQV-MSEVYLGRQICNIVACEGAERT-ER-HETL-VQWRGRLGGSGFEPVHL
```

Development • Supplementary information

```
GSNA-YKQASTLL--ALFAG------GD--GYRVE---EK--DGCLTLGWHTRPLIATSAWRVAAP----
--------------------------
>Seita.7G305500.1.p
----------------------------------------------------------------------
--------MGSSSLLLFPSSSSAASAA-----YSHATA--STSSHSSLLPPLP-----------------
-SSSSSPQ----DHHLLQYLHHLDQQH-Q----------------ESAAMVRKRPAPDMD----------
----------------------------------------------------------------------
----------LPPPRRHVTGDLSDVTAAAASG-GGAPPPQ--SAAASAQLPALPTQL---HL--P-AFQV
QHHHHHHHAEVDAP----------------PAGEVAA----STTAWVDGIIRDIIGS----SGGAGVSVA
QLIHNVREII-------HPCNPGLASLLELRLRSLLN---------------------------------
----------------------------------------------------------------------
--------------------------------------------------------------------AA
DPAAPLHQPLPHP---------------PALP-PVPALPPPPPPQLTVTDKRRH-------E------PQ
QAGEPTNPSPQQEEPK------PP-----------------------------TAEETAAAAAAAAAAAA
AAAKERKEEQRR--------------------KQRDEEGLHLL-TLLLQCAESVNADNLDDAHQTLLEIA
ELATPFGTS-TQRVAAYFAEAMSARLVSSCL-------GLYAPLPPAS-PA----AARLHGR--VAAAFQ
V-FNG---------------ISPFVKFSHFTANQAIQEAFEREERVHIIDLDIM-QG--L----QWPGLF
HILA-SRP----GGP----P-RVRLTGLGASME------ALQATGKRLSDFADTLGLPFEFC-AVA-EKA
GNV-DP------EKL----------GVT-------RREAVAVHWLH--HSLY-DVTGS---DSNTLWLIQ
R-LAPKVVTMVEQDLS--H---------------SGSFLARFVEAIHYYSALFDSLDAS-----------
-----YG------EDS-PERHVVEQQLLSREIRNVLAVGGPAR--TG-DVKF-GSWREKLAQSGFRSASL
AGSA-AAQASLLL--GMF-P------SD--GYTLV---EE--NGALKLGWKDLCLLTASAWRPIQTP-CR
--------------------------
>Brast10G114300.1.p
----------------------------------------------------------------------
----------------------------------------------------------------------
-----------------------MCSRMGTVNCPDTTS-------SVKLQ-QQQGPTSPT----------
----------------------------ASFSESNIVAS-------STDPDAIDALAGLQALRFD-----
GDI-DGEIQSPDLAMWESLFADQIGAS--GADFLMS-SPRRDFSP-----LRDFMVS-------------
-----SPKRDYMV---------------SSPKRDYMM-SSPKR-DYMMSSPKRDYMVSSPKREMGVSSPR
RSTFSNLYSS-------TINQA-------NQQSYMHSMEG--------SPQMQY-SNLASHG----NKGK
SSPSPLHKVYINNINAHSNSS--KSNGPSSLSCSSSY-THG---ENLPLPSMDP-FLE-----EYKEGYL
---AYQLPEKA-----------GGSESAGTT-----APISSQ-LPTLSECLAMPEPGY----GGGDVEAA
AAMVGR-GIQVGGLQQPDHLYYAGQFGAAE-----ESLS-------------------------------
-------SLQHQMAKP------EQW--ADS-S---------------------SLHSMLGS---------
----VIQSEAHQ--------------------QQDQDSGLQLV-HLLLACADFVSKGDQASALRHLHLLR
RVASPLGDS-MQRVASYFADALAARLSLT-------------CPSAVVSPGGAPFPFPPSPD--TLKIYQ
I-LYQ---------------ACPYIKFAHFTANQAIFEAFQGDDRVHVVDLDIL-QG--Y----QWPAFL
QALA-ARP----GGP----P-TLRLTGVGHPAA------AVRETGRHLASLAASLRVPFEFHAAVA-DKL
ERL-RP------ATL----------QRR-------VGEALAVNAVNRLHRVP---GAH---LAPLLSMIR
D-QAPKIMTLVEQEAG--HN--------------GPYFLGRFLEALHYYSAIFDSLDAT-----------
-----FP------ADS-APRMKVEQCLLAPEIRNVVACEGAERV-AR-HERL-DRWRRIMEGRGFEAVPL
SPAA-VGQSQVLL--GLYGA------GD--GYRLN---ED--KGCLLLGWQDRAIIGASAWRC-------
--------------------------
>Brast06G075300.1.p
----------------------------------------------------------------------
----------------------------------------------------------------------
----------------------------------------------------------------------
----------------------------------------------------------------------
----------------------------------------------------------------------
-----------------------------------------MANTPTSRMM-HPFGSVPRQNLKQFQYSD
NAQHPYHP---------YQSSPDTHVVPQHDYSLK----SHSP-DAGFENQVTH----------------
```

Development • Supplementary information

```
----------MKYTLDSSAAVGCMRH--------DSPSSHSFTPRSD--------SGSPL--SQEDSHS-
DST--NGS--PVSASCVTVT-EDPNDLRQKLKDLE-AVMLG---PDSEAVNSLESSIA--NQLS------
----------------------------------------------------------------------
----------------------------------------------------------------------
----LEPEKWV---------------------QMMRFPRDNLK-ELLVACARAVEEKNSYAIDMMIPELR
KMVSVSGEP-IQRLGAYMVEGLVARLASSGH-------SIYKALK---------CKEPKSSD--LLSYMH
F-LYE---------------ACPYFKFGYMSANGAIAEAVKGEDRIHIIDFHIS-QG--A----QWISLL
QALA-ARP----GGP----P-TVKITGIDDSVSAYARDGGLDIVGRRLSHIAGLCKVPFEFH-AVA-ICG
DEV-EE------GHL----------GVI-------PGEALAVNFTLELHHISDETVSTANHRDRILRLVK
S-LSPNVLTLVEQESN--TN--------------TAPFVQRFAETLDYYTAIFESIDLT-----------
-----LP------RDD-KERINMEQHCLAREIVNLVACEGSERV-ER-HEVF-GKWKARLMMAGFRPSPL
SSLV-NDTIRALL--QSY--------SV--NYQLA---ER--DGVLYLGWKNRPLVVSSAWH--------
--------------------------
>Aco031731.1
----------------------------------------------------------------------
----------------------------------------------------------------------
----------------------------------------------------------------------
----------------------------------------------------------------------
----------------------------------------------------------------------
----------------------------------------------------------------------
----------------------------------------------------------------------
----------------------------------------------------------------------
----------------------------------------------------------------------
----------------------------------------------------------------------
----------------------------------------------------------------------
-----------------------------------------MY-KLNIFC--------------------
---------------------------------------VYSPLIHHHKPL----VTSHHNR--IFNAFQ
V-YNS---------------ISPLVKFSHFTSNQAIFQALDGEERVHIIDLDIM-QG--L----QWPGLF
QILA-SRP----LKL----R-SLRITGLGSSLE------LLEATGRRLSDFAAALGLPFEFQ-PVE-GKI
GDQSKAGGGG--GAF-A--------PRD-------GREVAVVHWMH--HCLY-DVTGS---DEAAVSVIK
NVIRPRLVTVVEQDLG-RH---------------GGDFLGRFVEALHYYSAVFDALGEG-----------
-----AAAEGSREEEE-AERHDVERQLLGAEIRNIVAVGGPKR--TG-EVAV-GRWGEEMRRAGFEQVSL
AGGPAAAQANLLL--GMRLP------WK--GYTLV---EE--AGCLKLGWKDLSLLTASAWKTGNSHGPA
EGGEDRDHVQPPRQQQQHHHHSSLLT
>Pp3c22_13060V3.1.p
----------------------------------------------------------------------
----------------------------------------------------------------------
-------------------------------MRCDAKEPY-----QSPVPIMNAAPRDDW----------
----------------------------GKI--ADALSHHQQLPSHNDKAQQGSNGCGAQTREEAR----
----------------------------------------------------------------------
--------------------------------DQSAEMDEDSIAAWVDGTITEMMEA------MPGVAIE
QLFTNLPEFL-------SPCNLHLKGLIGCRLQLLLGGAGYPSKNFPPPGPNRS-GKRTR-------EVQ
VEWTNSKTFQNESQDL------------------------------------------------------
-------------------------------------HETLPRHHVRPENFISQKKVA--ASLSRVDLRC
P------KPLKRSIDNLQLSLEPADERNLRHLHIGLQATQKDQSSHQVRHSRAQSTEPVQ-QHYHKLHTH
NVQDKADGFLQRSPAP------SSASQFST-L---------------------NTMGSEDPPSQPQKLPQ
TTAQNAQRED----------------------TVAPDEGLQLM-SLLLQCAEAISADDNNQATAILPQLS
ELATPFGTS-VQRVVAYFAESMGSRLVTSSL-------GICRPLPCK--------QPASNQS--IVSAMQ
V-FNE---------------ICPFVKFSHFTANQAIAEAFEGKFNVHIIDVDIM-QG--L----QWPSLF
QVLA-SRA----GGP----P-HVHITGLGTSAE------SLDATGKRLKDFAGSFGISFEFT-AIA-DKM
SNV-DI------STL----------KVA-------FSDALAVHWMH--HSLY-DVTGS---DLDTLSLIQ
K-LNPKVITLVEQDFR--H---------------SGTFLSRFLEALHYYSAMFDSLGAT-----------
```

Development • Supplementary information

```
-----CK------DDS-PERYMVEQQLLSCEIKNIVAFDGPGR--KI-NHKF-DQWRDELSKAGFKPVSL
SGKA-SHQAALLL-QSLF-P------CD--GYTLL---EH--SGSLKLGWKDLYLFTASAWTRV------
--------------------------
>LOC_Os07g36170.2
----------------------------------------------------------------------
----------------------------------------------------------------------
----------------------------------------------------------------------
----------------------------------------------------------------------
----------------------------------------------------------------------
----------------------------------------------------------------------
---------------------MDLHQLLKYRLTGANVVYEIPTENNLQNSPWQA----------------
--------NPLKYEFSDSPYTPLSSQFECDNLSALTNTPDNQ-SSTETISAQ---PISPLEADSSYRQAG
ILLQENIQVGADPLYA-----TSRHNMQHALREIE-TVLMA---PDTDDATTSTKHEFEEIKPAQLVRQR
SRTWSHESRQ------------------------------------------------------------
-----------------------------------------------------PLPGVGRSQFASGGYPT
ASYEFRPEKRQR--------------------ELREDPQIIVK-QLLTRCAEALSEDRTEEFHKLVQEAR
GVVSINGEP-IQRLGAYLLEGLVARHGNSGT-------NIYRALK---------CREPESKE--LLSYMR
I-LYN---------------ICPYFKFGYMAANGAIAEALRTENNIHIIDFQIA-QG--T----QWITLI
QALA-ARP----GGP----P-RVRITGIDDPVSEYARGEGLDIVGKMLKSMSEEFKIPLEFT-PLS-VYA
TQV-TK------EML----------EIR-------PGEALSVNFTLQLHHTPDESVDVNNPRDGLLRMVK
G-LSPKVTTLVEQESH--TN--------------TTPFLMRFGETMEYYSAMFESIDAN-----------
-----LP------RDN-KERISVEQHCLAKDIVNIIACEGKDRV-ER-HELL-GKWKSRLTMAGFRPYPL
SSYV-NSVIRKLL--ACY--------SD--KYTLD---EK--DGAMLLGWRSRKLISASAWH--------
--------------------------
>LOC_Os07g39470.1
----------------------------------------------------------------------
----------------------------------------------------------------------
----------------------------------------------------------------------
----------------------------------------------------------------------
----------------------------------------------------------------------
-----------------------------------------MADTPTSRMI-HPFSNIPSQNLKQFQYSD
NPQHPCHP---------YRAPSDTHVVPHH-YGLK----SHSP-DAGYESQATP----------------
----------NKYTLDSSEGAGCMRH--------DSPSSQSFTTR----------SGSPL--SQEDSHS-
DST--DGS--PVGASC--VT-EDPNDLKQKLKDLE-AVMLG---PDSEIVNSLENSVA--NQLS------
----------------------------------------------------------------------
----------------------------------------------------------------------
----LEPEKWV---------------------RMMGIPRGNLK-ELLIACARAVEEKNSFAIDMMIPELR
KIVSVSGEP-LERLGAYMVEGLVARLASSGI-------SIYKALK---------CKEPKSSD--LLSYMH
F-LYE---------------ACPYFKFGYMSANGAIAEAVKGEDRIHIIDFHIS-QG--A----QWISLL
QALA-ARP----GGP----P-TVRITGIDDSVSAYARGGGLELVGRRLSHIASLCKVPFEFH-PLA-ISG
SKV-EA------AHL----------GVI-------PGEALAVNFTLELHHIPDESVSTANHRDRLLRMVK
S-LSPKVLTLVEMESN--TN--------------TAPFPQRFAETLDYYTAIFESIDLT-----------
-----LP------RDD-RERINMEQHCLAREIVNLIACEGEERA-ER-YEPF-GKWKARLTMAGFRPSPL
SSLV-NATIRTLL--QSY--------SD--NYKLA---ER--DGALYLGWKSRPLVVSSAWH--------
--------------------------
>GRMZM2G015080_P01
----------------------------------------------------------------------
--------MGSSSVLLFPSSSSAAPSAPHSFPHSHATAIASSH---SLLPPLP--------------CSN
PPPPLSSQ----DHVLIHYIHQLDEQE-------------------AATMVRKRPAPDMD----------
----------------------------------------------------------------------
----------LPPPRRHVTGDLSDVTAAAAAG-GGPGAPS----SASAQLPALPTQL---HQLPP-AFQ-
-----HHAAEVDVP------PQPHPPAHSQAGGEAPA----STTAWVDGIIRDIIGS----SGGGAVSIT
```

Development • Supplementary information

QLIHNVREII-------HPCNPGLASLLELRLRSLL----------------------------------
----
--AA
DP-APL------PQQQRALL---H--GAPAAA-AGLALPLPPP----LPDKRRH-------E---PAPRC
QQQQQEEPHPAPQSPK------VP-----------------------------TAEET-------AAASA
AAAKERKEVQRR--------------------KQRDEEGLHLL-TLLLQCAEAVNADNLDDAHQTLLEIA
ELATPFGTS-TQRVAAYFAEAMSARVVSSCL-------GLYAPLPPGS-PA----AARLHGR--VAAAFQ
V-FNG---------------ISPFVKFSHFTANQAIQEAFEREERVHIIDLDIM-QG--L----QWPGLF
HILA-SRP----GGP----P-RVRLTGLGASME------ALEATGKRLSDFADTLGLPFEFC-AVD-EKV
GNV-DP------QKL----------GVT-------RREAVAVHWLH--HSLY-DVTGS---DSNTLRLIQ
R-LAPKVVTMVEQDLS--Q---------------SGSFLARFVDAIHYYSALFDSLDAS-----------
-----YG------EDS-PERHVVEQQLLAREIRNVLAVGGPARAGAG-GARF-GSWREELARSGFRAASL
AGGA-AAQASLLL--GMF-P------SD--GYTLV---EE--KGALRLGWKDLCLLTASAWRPVQTPPCR

>Sevir.8G008100.1.p

--------MGSSSLLLFPSSSSAASPA-----YSHATA--STSSHSSLLPPLP-----------------
-----SPQ----DHHLLQYLHHLDQQQ-Q----------------ESAAMVRKRPAPDMD----------
----
----------LPPPRRHVTGDLSDVTAAAASG-GGAPPPQ--SAAASAQLPALPTQL---HL--P-AFQV
QHHHHHHHAEVDAP----------------PAGEVAA----STTAWVDGIIRDIIGS----SGGAXVSVA
QLIHNVREII-------HPCNPGLASLLELRLRSLLN---------------------------------
----
--AA
DPAAPLHQPLPHP---------------PALP-PVPALPPPPPPQLTVTDKRRH-------E------PQ
QXGEPTNPSPQQEEPK------PP-----------------------------TAEETAAAAAAAAAAAA
AAAKERKEEQRR--------------------KQRDEEGLHLL-TLLLQCAEXVNADNLDDAHQTLLEIA
ELATPFGTS-TQRVAAYFAEAMSARLVSSCL-------GLYAPLPPAS-PA----AARLHGR--VAAAFQ
V-FNG---------------ISPFVKFSHFTANQAIQEAFEREERVHIIDLDIM-QG--L----QWPGLF
HILA-SRP----GGP----P-RVRLTGLGASME------ALQATGKRLSDFADTLGLPFEFC-AVA-EKA
GNV-DP------EKL----------GVT-------RREAVAVHWLH--HSLY-DVTGS---DSNTLWLIQ
R-LAPKVVTMVEQDLS--H---------------SGSFLARFVEAIHYYSALFDSLDAS-----------
-----YG------EDS-PERHVVEQQLLSREIRNVLAVGGPAR--TG-DVKF-GSWREKLAQSGFRSASL
AGSA-AAQASLLL--GMF-P------SD--GYTLV---EE--NGXLKLGWKDLCLLTASAWRPIQTP-CR

>Aco012593.1

--------MGSSSLLLLPPTTTTTTTTNTTSSSHDSHFSHYS----------------------------
------------EEEKELLYLLYLQQQQQQQQQQQQQQQV-----GEVIMVRKRPASELDLQHESSSSSY
HNPRLPRRGGGGGAA-------------AAGGGASSSTAAAQGPFAES--------------------AA
AGHPSSLLYSASSGGGALDAPILVQHGGNPTQFLPSLPPPAPAVCGFSGLPLFPAEL-------------
-----ERGGGGGAAAANEGV------------AGRGGADEGAASAWVDGIIRDLLSS-----AAPAVSIP
QLIHNVREVI-------HPCNPALAAVLEHRLRSLA----------------------------------
----
--SS
DPLPPPPPPPPPLPSL--
-------------PSD------APSLTSSP-P---------------------RARPSPAPAPTGPASSQ
VATK----------------------------PTTAAAAAAQL-AGGARCAESVAADNLDEANRLLLEIS
ELATPFGSS-AQRVAAYFSEAMSARLVSSCL-------GLYAPLPASS-PA----ALRVHHR--LAAAFQ
V-FNG---------------ISPFVKFSHFTANQAIQEAFDREERVHIIDLDIM-QG--L----QWPGLF
HILA-SRP----GGP----P-RVRLTGLGASLD------ALEATGKRLSDFADTLGLPFEFH-PVA-DKV
GNL-DP------DRL----------GVS-------RREALAVHWLH--HSLY-DVTGS---DTNTLWLLQ

```
R-LAPKVVTMVEQDLS--P---------------GGSFLARFVEAIHYYSALFDSLGAS-----------
-----YG------EDS-PERHVVEQQLLSREIRNVLAVGGPAR--SG-DVKF-GNWREKLAQSGFRSVSL
AGNA-AAQATLLL--GMF-P------SD--GYTLV---EE--NGALKLGWKDLCLLTASAWRPIQTANPP
R-------------------------
>Sobic.005G017500.2.p
----------------------------------------------------------------------
--------MGSSSLLLFPSSSSAASTAPHAFPHSHATATASTSTSTSLLPPLP------------SPHHL
PLPPPSSSSQSQDHFLLHYLHQLDRQEAA----------------AAAAMVRKRPAQDMD----------
----------------------------------------------------------------------
----------LPPPRRHVTGDLSDVTAAAAG--SGGAPPS----SASAQLPALPTQL---HQLPP-AFQ-
-----HHAAEVGVPVPAPPPPPPNPAAHAQAGGEAAA----STTAWVDGIIRDIIGS----SGGAAVSIT
QLIHNVREII-------HPCNPGLASLLELRLRSLL----------------------------------
----------------------------------------------------------------------
--------------------------------------------------------------------AA
DP-APLPPPPPQPSQQHALL---HGVGAPAAA-AGLTLPPPPP----LQDKRRH-------EPPPPPPPP
QQQQQEEPHPASQSPK------AP-----------------------------TAEETAAAAAAAAAAAA
AAAKERKEEQRR--------------------KQRDEEGLHLL-TLLLQCAEAVNADNLDDAHQTLLEIA
ELATPFGTS-TQRVAAYFAEAMSARLVSSCL-------GLYAPLPPGT-PA----AARLHGR--VAAAFQ
V-FNG---------------ISPFVKFSHFTANQAIQEAFEREERVHIIDLDIM-QG--L----QWPGLF
HILA-SRP----GGP----P-RVRLTGLGASME------ALEATGKRLSDFADTLGLPFEFC-AVA-EKA
GNV-DP------EKL----------GVT-------RREAVAVHWLH--HSLY-DVTGS---DSNTLWLIQ
R-LAPKVVTMVEQDLS--H---------------SGSFLARFVEAIHYYSALFDSLDAS-----------
-----YG------EDS-PERHVVEQQLLSREIRNVLAVGGPAR--TG-DVKF-GSWREKLAQSGFRAASL
AGSA-AAQASLLL--GMF-P------SD--GYTLV---EE--NGALKLGWKDLCLLTASAWRPIQMPPCR
--------------------------
>GRMZM2G342217_P01
----------------------------------------------------------------------
----------------------------------------------------------------------
---------------------------MGMLNCVDTTSGA-----GAKLQHQQQAPTSPT----------
----------------------------ASVSESNIVAPS-----VSADIDANDALASLQALRFD-----
GDI-DVEIQSPDIAMWESLFAEQMGAA--GGDFLML-SPRRDFMAAGSPSRRDFMVS-------------
-----SPKRDYMV-S-------------SSPKRDYMM-SSPMR-DLMASSPKREYMVSSPRRD--SSSSR
RSPFSPNLFS-------TSGGG-------HQHGYAA--HGPTDGGVGGAGQPLY-GGLANHH----GKGK
SQ-SPLHKGYINN------SGNKQSTGPSSLSCSSSY-GHA----DNDLPSMDTCFLD-----DYKDGGY
IGYQQQMPGKQQAAPGIMVNNNGGCSTAVTTVGVAPSPSSHQ-LPTLSECLAMPEPAF----VGGEEAAA
G------GLQMG-VGLPSDLYYAGQFAGGGGGGLTTTSS-------------------------------
-------SLQHQMAKS------DHQWAAAE-S---------------------SLHSMLGS---------
----VIQTEAD---------------------EQEQDSGLQLV-HLLLACADFVSKGDQPSALRHLHLLR
RVASPLGDS-MQRVASYFADALAARLS-SNNPSS--------SAGAGAGAGVAPYTFPPSPD--TLKVYQ
I-LYQ---------------ACPYIKFAHFTANQAIFEAFHGEDRVHVVDLDIL-QG--Y----QWPAFL
QALA-ARP----GGP----P-TLRLTGVGHPAA------AVRETGRHLASLAASLRVPFEFHAAVA-DRL
ERL-RP------AAL----------HRR-------VGEALAVNAVNRLHRVP---AVH---LGPLLSMIR
D-QAPKIMTLVEQEAG--HN--------------GPYFLGRFLEALHYYSAIFDSLDAT-----------
-----FP------ADS-AQRMKVEQCLLAPEIRNVVACEGAERV-AR-HERL-DRWRRIMEGRGFEPVPL
SPAA-VAQSQVLL--GLYGA------GD--GYRLT---ED--RGCLLLGWQDRATIAASAWRC-------
--------------------------
>Pp3c11_23910V3.1.p
----------------------------------------------------------------------
----------------------------------------------------------------------
----------------------------MTATHVASNKRS-----STSFPEFVTAPCKRW----------
----------------------------KSEAAWHHVEEIVPEPPPAQDFDLKVKTCYSN----------
------------------------------------------------------MVH-------------
```

```
-----PEPTSVLDLQ-------------TSPGRSC-SSTSLSSGT-----------------DSPHSVST
DSSNFSTTLQ-------QVEQDDIANWVDCMGLPEFEECGRPLEEVLKSDIDFN----------------
--TTDIDFGFEMTSLEHCSAVAEHDFSVSLLSDFLGEP--------------------------------
------------------------------------DENLLLPESFHYAKRLQELDDS--LSSMLNEVRS
SGSDSESSI-------------------------------------------------------------
-------PTTAELVRL------VEAQSCED-L---------------------GRQRACGAR-NHHHSRA
GSLGSTSEPQTL--------------------QQPENSGLQLV-HLLLACAEAIDKSHFHKANPILDQLG
RFSNAYGGP-MQRIALYFGNALSNHLA-----------GVVSP-----------TDPHSPSD--SKFAYQ
A-FYK---------------ILPFAKFSHVTANQTIYEAVLRSQNVHVVDLDIQ-QG--L----QWPCFI
QSLA-MRP----GGA----P-HLRISAVGMNME------SLQTTKRWLTEFAEDLKVPFEFT-PVL-STL
ENL-TP------AML----------NIR-------ADEDLAINCSQVLHTLS-GDEAV---LEKLLCMFR
N-LRPNVVTLLEAEAN--YN--------------AASFITRFIEALHYYCALFDSLEGA-----------
-----LG------RDS-ADRFHIESTAFAAEINDILASKDSSRR-VR-HVRS-ETWRALFKKAGFRSMAF
SSYT-VRQAQMLL--EILTSKHLMQANSPIPYKLS---EE--STSLILGWQETPVIGVSAWSC-------
--------------------------
>Sevir.2G354000.1.p
----------------------------------------------------------------------
----------------------------------------------------------------------
----------------------------------------------------------------------
----------------------------------------------------------------------
----------------------------------------------------------------------
----------------------------------------------------------------------
---------------------MDLHQLLKYRLTGANVFYEFPTENNLANNPWPG----------------
--------TPLKSEFSNSPYTPLSAQLECDNLSAISNTPDNQ-SSTETISAQ---PISPLEVDSSNRQAG
I-LWENTQVRPDHLYT-----TSRHNMQHALRKIE-TVLMA---PDADDAATSTKHEFEEHKPAPLMRQR
SRTWSHELRQ------------------------------------------------------------
-----------------------------------------------------PSPGVVRTQFAS-GYPT
ASYEFRPEKRQR--------------------ELREDPQSMVK-QLLTKCAEALSEERIEEFLKLVQQAR
GVVSITGEP-IQRLGAYLLEGLVARHGNSGT-------NIYRALK---------CREPESNE--LLSYMK
I-LYN---------------ICPYFKFGYMAANGAIAEALRNEDKIHIVDFQIA-QG--T----QWITLI
QALA-ARP----GGP----P-HVRITGIDDPVSEYARGEGLDLVGKMLKSMSEEFRIPLEFT-PLPGVYA
TQV-TK------EML----------DIR-------PGEALAVNFTLQLHHTPDESVDVNNPRDGLLRMVK
G-LSPKVTTLVEQESH--TN--------------TTPFLMRFSETMDYYSAMFESIDAN-----------
-----LP------RDS-KERINVEQHCLAKDIVNIIACEGKDRV-ER-HELL-GKWKSRLTMAGFKPYPL
SSYV-NSVIRKLL--ACY--------SD--KYTLE---EK--DGAMLLGWKSRKLISASAWH--------
--------------------------
>Aco022504.1
----------------------------------------------------------------------
----------------------------------------------------------------------
-----------------------------------MLAGW-----HQYLRSEASLQLQVC----------
----------------------------QFQLQEKMGTQRLDLPCGFAKRENHPRVSLS-----------
----------------------------------------------------------------------
----------------------------------------------LEKPIDPRSTSCSFRQNPLAAPSS
SSL--------------------VAKIEPTDQGALWEESSRRLKRFHERDCFEE-SILARAKRTRTGEAG
QLVGGGGGSGGGDEDIWFPESIERTPFV-----------------------------------------E
EEKVFLVP-------------------------------SAASFPVDNTSLVGSFESE---KDFEWPNTK
SQSDS-----------------------------------------SSSSGT------------------
-------GGLSPKPEI------ETE------------------------------------NRNDVVAYT
VGGAGVDES-----------------------TRIESEGLGLL-NLLTSCVEAIGAGNHEAVNYLLARLG
EQASPKGTP-IHRVVAYFTEAVALRVAKLWP-------HIFSITSPRELTD----HLEDEEN--DAIALR
L-LNH---------------VSPIPKFLHFTCNERLLRAFEGKERVHIVDFDIK-QG--L----QWPSLF
QSLA-SRP----QPP----A-HVRITGVGECRQ------ELLNTGVRLSRFAEALNLPFEFH-AVT-DRL
```

Development • Supplementary information

```
EDV-RL------WML----------HVK-------RDECVAVNCVLVMHKLLYDETGSA--LMDFLGLIR
S-VKPEVVLTAEQEAD--HN--------------DERWELRLGKSVQYYSAVFDSLDLC-----------
-----LA------SDSPAARAKIEQ-MFAREIRNLVACEGDERF-ER-HERF-SKWIKKMEDGGYRSAGI
GERE-IVQSQLIL--RMYA-------SD--KYSVEKRGEGV-SEGLSLRWVDQPLYTVSAWALSEVGGSS
STSLPC--------------------
>Sevir.8G136700.1.p
----------------------------------------------------------------------
----------------------------------------------------------------------
---------------------------MGMLNCADTSS-------GAKLQ-QLQAPTSPT----------
----------------------------ASVSESNIVVS-------STDPDANDALAGLQALKFD-----
GDI-DVEIQSPDIAMWESLFAEQMGAS--GGDFLMSFSPRRDFTATGSP-RRDFMVS-------------
-----SPKRDYMM---------------SSPKRDYMV-SSPKR-DYMMSSPKREYMVTSPRRD---SSPR
RSTFSNLYSG-------TGS---------HQQGYVDGVHGAEGGSGSGGGQPLY-GGLANH-----GKGK
SQ-SPLHKVYINN--AHSNGG--RSTGPSSLCSSSY-GHG---ESLSLPSMDP-FLD-----EYKEGGG
YLAGYQLPVKT--------GMENGAASAATVTTVAPSPSQ---LPTLSECLAMPEPVY----GGSEAAAA
G------GLQMG-AGLPAELYYGGQFGGDGF---------------------------------------
-------TLQHQMAKS------DQW--AGD-S----------------------SLHSMLGS---------
----VIQTEA----------------------EQEQDSGLQLV-HLLLACADFVSKGDQPSALRHLHLLR
RVASPLGDS-MQRVASYFADALAARLSLSSNPSSSSSSSGAATPRGGAAAGVAPYTFPPSPE--TLKIYQ
I-LYQ---------------ACPYIKFAHFTANQAIFEAFAGEDRVHVVDLDIL-QG--Y----QWPAFL
QALA-ARP----GGP----P-TLRLTGVGHPAA------AVRETGRHLASLAASLRVPFEFHAAAA-DRL
ERL-RP------AAL----------QRR-------VGEALAVNAVNRLHRVP---SAH---LGPLLSMIR
D-QAPKIMTLVEQEAG--HN--------------GPYFLGRFLEALHYYSAIFDSLDAT-----------
-----FP------ADS-APRMKVEQCLLAPEIRNVVACEGAERV-AR-HERL-DRWRRLMEGRGFEPVPL
SPAA-IGQSQVLL--GLYGA------SD--GYRLT---ED--KGCLLLGWQDRAIIAASAWQC-------
--------------------------
>LOC_Os07g38030.1
----------------------------------------------------------------------
----------------------------------------------------------------------
---------------------------MLQGVLSRAPGAD-----AAAMK-AKRAADD------------
----------------------------------------------------------------------
----------------------------------------------------------------------
----------------------------------------------------------------------
----------------------------------------------------------------------
----------------------------------------------------------------------
----------------------------------------------------------------------
----------------------------------------------------------------------
-------------EEE------GGERERAR-G---------------------KRLAAEGKQGLVVVSTG
EEEE----------------------------AAAETRGLRLL-SLLLRCAEAVAMDQLPEARDLLPEIA
ELASPFGSS-PERVAAYFGDALCARVLSSYL-------GAYSPLA--LRPL----AAAQSRR--ISGAFQ
A-YNA---------------LSPLVKFSHFTANQAIFQALDGEDRVHVIDLDIM-QG--L----QWPGLF
HILA-SRP----TKP----R-SLRITGLGASLD------VLEATGRRLADFAASLGLPFEFR-PIE-GKI
GHVADA------AALLG--------PRH-------HGEATVVHWMH--HCLY-DVTGS---DAGTVRLLK
S-LRPKLITIVEQDLG--H---------------SGDFLGRFVEALHYYSALFDALGDG-----------
-----AGA---AEEEA-AERHAVERQLLGAEIRNIVAVGGPKR--TG-EVRV-ERWGDELRRAGFRPVTL
AGSP-AAQARLLL--GMY-P------WK--GYTLV---EE--DGCLKLGWKDLSLLTASSWEPTDGDADA
DVAVAGDTHHESHDS-----------
>AT1G66350.1
----------------------------------------------------------------------
----------------------------------------------------------------------
---MKREHNHR---------ESSAGEGGSSSMTTVIKEEA-----AG--------VDELL----------
----------------------------VVLGYKVRSSDMADVAHKLEQLEMVLG---------------
```

Development • Supplementary information

---D
GISNLSDETV-------HYNPSDLSGWVESMLSDLDPTRIQE----------------------------
----

---KPDSEYDLRAIPGSA-VYPR------------------DEHVTRRSKRT------------------
---------RIESELS------STR---
--------SVVV--------------------LDSQETGVRLV-HALLACAEAVQQNNLKLADALVKHVG
LLASSQAGA-MRKVATYFAEGLARRIYRIYPRDD-----------------------VALSS--FSDTLQ
IHFYE---------------SCPYLKFAHFTANQAILEVFATAEKVHVIDLGLN-HG--L----QWPALI
QALA-LRP----NGP----P-DFRLTGIGYSLT------DIQEVGWKLGQLASTIGVNFEFKSIAL-NNL
SDL-KP------EML----------DIR------PGLESVAVNSVFELHRLL-AHPGS---IDKFLSTIK
S-IRPDIMTVVEQEAN--HN--------------GTVFLDRFTESLHYYSSLFDSLEGP-----------
--------------PS-QDRV-MSELFLGRQILNLVACEGEDRV-ER-HETL-NQWRNRFGLGGFKPVSI
GSNA-YKQASMLL--ALYAG------AD--GYNVE---EN--EGCLLLGWQTRPLIATSAWRINRVE---
----
>Pp3c7_1494V3.1.p

----------------------------------MADGDL-----ASIFRQHTQPVSDPW----------
----------------------------GVAVPQHQLSHFQQMPQTLQDTDNVRWDY-------------
----
---PNS
LSPQSPFDIQ-------DHNSNFSTRMLQSKVGSYNHSYSSLRPSTIP----------------------
----------------PQSHLQFSTISPQLVEPVQSLQSQTLSGNKRDF---------------------
QGSSFQVQNKQPTRSAGRNIYSEVVTSTSPVGQENLVTVSHHQSQFSGNNYNISHGQP--STWPTAPTGC
KVGE-------------------------------------LATELQPEILGDLRPGPGYLADHYPGRLL
QSGQAYNQNQNLGPQG------NPNDLPSG-S---------------------GTLSSSLQQNFPGDDGM
IRVLDTACSRRN--------------------QQTSATGLQLV-HLLLLCAEAISNQQMDLAHVVLTRLN
AMLVPCTST-MQRLAAVFVDALHARITNSATT------GRYKGLER--------DNDVAILD--MLQSFS
V-IYD---------------HTPFIKLPHLTLNQIILDAVEGEPHVHVIDLNTGWRG--M----QWPGFI
QALA-LRP----GGP----P-KLRITAIGKAD-------DLEHSREKLQDYARHLQVPFEFC-PLV-VDM
KSF-DV------RLL----------DMR-------DWEVVCINSANQFHQLLIWGDEC---FHKFLCDLK
S-LNPRVLAFTENDAD--HN--------------SPKFLNRFFECLRYYSAVYDALDSS-----------
-----LP------NGS-AALQQVEHLFTGQKIRNIVAMEGEDRI-TR-HESL-TSWSRRMEMAGFRPVPV
SSRA-ISQAGLLL--RMYFA------QS--GYTLR---TE--NGNVSLGWDNMSLVGASAWRA-------
----
>Sobic.002G354900.1.p

---MADTPTSRMV-HPFGDVPRQTPKQFLYSG
NSQHLCYP---------YQSASDTHVVPQRHCTMR----SHSP-DAGSEDHDNH----------------
----------KQYTLDSSATSGCSRH--------DSPSSQSVHAG----------SGSPV--SLEDSHS-
GSTNGNGS--PVSASC--VT-EDPTDLKQKLKDLE-AAMLG---TDPEIVNSLEISIA--DQLS------
----

----LEPEEWK---------------------HMMSMPGGNLK-ELLIACARAVEYNNSYAIDLMIPELR
KKVSVSGEP-LERLGAYMVEGLVARLAASGS-------SIYKALK---------CKEPRSSD--LLSYMH
F-LYE---------------ACPYFKFGYMSANGAIAEAVKGEDRIHIIDFHIA-QG--A----QWISLL

Development • Supplementary information

```
QALA-ARP----GGP----P-FVRITGIDDSVSAYARGGGLELVGRRLSHIAGLYKVPFQFD-AVA-ISS
SEV-EE------GHL----------GIV-------PGEAVAVNFTLELHHIPDETVSTANHRDRILRLVK
G-LSPKVLTLVEQESN--TN--------------TAPFAQRFAETLDYYTAIFESIDLA-----------
-----LP------RDD-RERINIEQHCLAREIVNLVACEGEERV-ER-HEVF-GKWKARLMMAGFRPSPL
SALV-NATIKTLL--QSY--------SP--DYKLA---ER--DGVLYLGWKNRPLIVSSAWH--------
--------------------------
>Bradi4g18390.1.p
----------------------------------------------------------------------
----------------------------------------------------------------------
-----------------------MCSRMGTLNCSDTTS-------SVKLQ-QQQGPTSPT----------
----------------------------ASFSESNIVAS-------STDPDAIDALAGLQALRFD-----
GDI-DGEIQSPDLAMWESLFADQIGAS--GADFLMS-SPRRDFSP-----LRDFMVS-------------
-----SPKRDYMV---------------SSPKRDYMM-SSPKR-DYMMSSPKRDYMVSSPKREMGVSSPR
RSTFSNLYSS-------TINQA-------NQQSYMHGMEG--------SPQTQY-SNLASQG----NKGK
SSPSPLHKVYINNVNAHSNSG--KSNGPSSLSCSSSY-AHG---ENLPLPSMDP-FLE-----EYKEGYL
---AYQLPEKA-----------GGSESARTT-----APTSSQ-LPTLSECLAMPEPGY----GDGDDDTA
AAIVARAGIQVGGLQQTDHLYYASQFGAAE-----GSLS-------------------------------
-------SLQHQMAKP------EQW--ADS-S---------------------SLHSMLGS---------
----VIQSEADQ--------------------QQEQDSGLQLV-HLLLACADLVSKGDQPSALRHLHLLR
RVASPLGDS-MQRVASYFADALAARLALA-------------CPSSVVSPGGAPFPFPPSPD--TLKIYQ
I-LYQ---------------ACPYIKFAHFTANQAIFEAFQGEDRVHVVDLDIL-QG--Y----QWPAFL
QALA-ARP----GGP----P-TLRLTGVGHPAA------AVRETGRHLASLAASLRVPFEFHAAVA-DKL
ERL-RP------AAL----------QRR-------VGEALAVNAVNRLHRVP---GAH---LAPLLSMIR
D-QAPKIMTLVEQEAG--HN--------------GPYFLGRFLEALHYYSAIFDSLDAT-----------
-----FP------ADS-APRMKVEQCLLAPEIRNVVACEGAERV-AR-HERL-DRWRRIMEGRGFEAVPL
SPAA-VGQSQVLL--GLYGA------GD--GYRLN---ED--KGCLLLGWQDRAIIGASAWRC-------
--------------------------
>Bradi1g23350.1.p
----------------------------------------------------------------------
----------------------------------------------------------------------
----------------------------------------------------------------------
----------------------------------------------------------------------
----------------------------------------------------------------------
-----------------------------------------MANTPTSRMM-HPFGSVPRQNLKQFQYSD
NAQHTCHP---------YQSSPDTHVVPQHDYSLK----SHSP-DAGFENQVTH----------------
----------MKYTLDSSAEVGCMRH--------DSPSSHSFTPRSD--------SSSPL--SQEDSHS-
DST--NGS--PVSASCVTVT-EDPNHLRQKLKDLE-AVMLG---PDSEVVNSLESSIA--NQLS------
----------------------------------------------------------------------
----------------------------------------------------------------------
----LEPEKWV---------------------QMMRFPRDNLK-ELLVACARAVEEKNGYAIDMMIPELR
KMVSVSGEP-LQRLGAYMVEGLVARLASSGH-------SIYKALK---------CKEPKSSD--LLSYMH
F-LYE---------------ACPYFKFGYMSANGAIAEAVKGEDRIHIIDFHIS-QG--A----QWISLL
QALA-ARP----GGP----P-TVKITGIDDSVSAYARDGGLDIVGRRLSHIAGLCKVPFEFH-AVA-ISG
DEV-EE------RHL----------GVI-------PGEALAVNFTLELHHISDETVSTANHRDRILRLVK
S-LSPNVLTLVEQESN--TN--------------TAPFVQRFAETLDYYTAIFESIDLT-----------
-----LP------RDD-KERINMEQHCLAREIVNLVACEGSERV-ER-HEVF-GKWKARLMMAGFRPSPL
SSLV-NDTIRTLL--QSY--------SV--NYQLA---ER--DGVLYLGWKNRPLVVSSAWH--------
--------------------------
>Solyc08g080400.1.1
----------------------------------------------------------------------
----------------------------------------------------------------------
----------------------------MLAGCSSSLLSP-----RHRLRSEASAQFQAC----------
```

```
----------------------------NFP---SMSTQRLDLPCSFARKDTSRSQSISRPVGLSVE---
----------------------------------------------------------------------
------------------------------------------------KPIEAKNSTCSLKQNIRLPPSS
TSAQTSYVEG-------RRESKD------EFWEKGRSLKRYAEQGCIDDESCMS-RAKRKKSNRKSGDSS
EDGYDLSLSQLGGGDFWLQSGFNAARSVPLIAGLHPPQAAPFSFSCSG---------------------E
EESVCYVP----------------------------NEVISPPLPMSNNPWIESVVTE--ITNFGDKNVS
TSQDLAR----------------------------------EASVSSASLDS------------------
-------HGLVLRPNE------NLGEHEVG-N---------------------GSRLPNPNDRGEVVTAH
NGHN----------------------------NHREDDAAELI-SLLVSCVEAIGSRNVTGVNQLIARLG
QLASPRGSP-VSRLTAYFTEALALRVARIWP-------HIFHIIPPRDL-------DRLDDD--SSTALR
L-LNQ---------------VSPIPKFIHFTSNEILLRAFEGKDRVHIIDFDIK-QG--L----QWPSLF
QSLA-SRP----NPP----T-HVRITGIGESKQ------DLVETGDRLAEFAEALNLAFEFH-PVV-DRL
EDV-RL------WML----------HVK-------EGESVAVNCVLQMHRLLYDSSGGI--LRDFLGLIR
S-TNPTIILMAEQEAE--HN--------------EPSLEARLVNSLRYYAAVFDSIAFG-----------
-----LP------LDS-PARIKIEE-LFARDIRNIIACEGRDRT-ER-HECF-GKWRKLMEQGGFRCTGI
TERE-LLQSQMLL--KMYS-------CE--DYRVTKQGND--DAALTLSWLDQPLCTVSAWTPIDAAGSS
SSYYQPS-------------------
>Brast02G278200.1.p
----------------------------------------------------------------------
----------------------------------------------------------------------
---MKREYQDA---------GGSRGGGDMGPSKDKMMVAA-----PA---EEED-VDELL----------
----------------------------AALGYKVRASDMADVAQKLEQLEMAMGMGGV-GAGAAPD---
----------------------------------------------------------------------
--------------------------------------------------------------------DG
FAAHLATDTV-------HYNPSDLSSWVESMLSELNAPPPPLPPA-------------------------
----------------------------------------------------------------------
--------------------------------------PQAPRLSCNSSTVTG----G---GGYFD-LPP
SVDSSSSTYALKPIPSPV-VTPA------------------EP-SADPAREP------------------
--KRMRTGGGSTSSSS------SSSSSLGG-G---------------------GTMSSVVEAAPP-----
-AANAPALPVVV--------------------VDTQEAGIRLV-HALLACAEAVQQENLSAAEALVKQIP
LLAASQGGA-MRKVAAYFGEALARRVFRFRPQPD---------------------SSLLDAA--FADLLH
AHFYE---------------SCPYLKFAHFTANQAILEAFAGCRRVHVVDFGIK-QG--M----QWPALL
QALA-LRP----GGP----P-SFRLTGVGPPQP--DETDALQQVGWKLAQFAHTIRVDFQYRGLVA-ATL
ADL-EP------FML----------QPEGEEDPNEEPEVIAVNSVFEMHRLL-SQPGA---LEKVLGTVR
A-VRPRIVTVVEQEAN--HN--------------SGSFLDRFTESLHYYSTMFDSLEGAGSGQS------
EISPGAA------AGA-TDQV-MSEVYLGRQICNVVACEGPERT-ER-HETL-GQWRGRLGQAGFETVHL
GSNA-YKQASTLL--ALFAG------GD--GYKVE---EK--DGCLTLGWHTRPLIATSAWRMAAP----
--------------------------
>Sobic.002G342800.1.p
----------------------------------------------------------------------
----------------------------------------------------------------------
---------------------------MLQGVLSRAPATDAAA--AAAMK-AKRSAASPG----------
----------------------------------------------------------------------
----------------------------------------------------------------------
----------------------------------------------------------------------
----------------------------------------------------------------------
----------------------------------------------------------------------
----------------------------------------------------------------------
----------------------------------------------------------------------
-------------EEE------EGDGRSARGG---------------------KRQQLLALGPATALASA
VAAE----------------------------EGPETRGLRLL-SLLLRCAEAVAMDQLTEARELLPEIA
ELASPFGSS-PERVAAYFGDALCARVLSSYL-------GAYSPLA--LRQL----AAAQSRR--VAVAFQ
```

```
A-YNA---------------LSPLVKFSHFTANQAILQALDGEDCLHVIDLDIM-QG--L----QWPGLF
HILA-SRP----RKP----R-SLRITGLGASLD------VLEATGRRLADFAASLGLPFEFH-PIE-GKI
GHVADA------AALLGSRQHQN--QQR-------DDEATVVHWMH--HCLY-DVTGS---DVGTVRLLR
S-LRPKLITIVEQDLG--H---------------SGDFLGRFVEALHYYSALFDALGDG-----------
-----AGA---AEEES-AERYAVERQLLGAEIRNIVAVGGPKR--TG-EVRV-ERWGDELRRAGFRPVSL
AGSP-ATQARLLL--GMY-P------WK--GYTLV---EE--DACLRLGWKDLSLLTASAWEPADDAAAS
APTTTS--------------------
>GRMZM2G131516_P02
----------------------------------------------------------------------
------------------------------------------------MPPPPPPPPLTPYCRRCPPPHL
PPPPPSSP----NHFLLHYLHQLDHQE-A----------------AAAAMVRKRPASDMD----------
----------------------------------------------------------------------
----------LPPPRRHVTGDLSDVTAAAAAGVGGSGAPS----SASAQLPALPTQL---HQLPP-AFQ-
-----HHAPEVDVPA------HPAPAAHAQAGGEATA----STTAWVDGIIRDIIGS----SGGAAVSIT
QLIHNVREII-------HPCNPGLASLLELRLRSLL----------------------------------
----------------------------------------------------------------------
--------------------------------------------------------------------AA
DP-APL-PPPPQP-QQHALL---H--GAPAAAPAGLTLPPPPP----LPDKRRH-------E---HPPPC
QQQQQEEPHPAPQSPK------AP-----------------------------TAEETAAAAAAAQAAAA
AAAKERKEEQRR--------------------KQRDEEGLHLL-TLLLQCAEAVNADNLDDAHQTLLEIA
ELATPFGTS-TQRVAAYFAEAMSARLVSSCL-------GLYAPLPPGS-PA----AARLHGR--VAAAFQ
V-FNG---------------ISPFVKFSHFTANQAIQEAFEREERVHIIDLDIM-QG--L----QWPGLF
HILA-SRP----GGP----P-RVRLTGLGASME------ALEATGKRLSDFADTLGLPFEFC-AVA-EKA
GNV-DP------EKL----------GVT-------RREAVAVHWLH--HSLY-DVTGS---DSNTLWLIQ
R-LAPKVVTMVEQDLS--H---------------SGSFLARFVEAIHYYSALFDSLDAS-----------
-----YG------EDS-PERHVVEQQLLSREIRNVLAVGGPAR--TG-DVKF-GSWREKLAQSGFRAASL
AGSA-AAQASLLL--GMF-P------SD--GYTLV---EE--NGALKLGWKDLCLLTASAWRPIQVPPCR
--------------------------
>GRMZM2G431309_P01
----------------------------------------------------------------------
----------------------------------------------------------------------
----------------------------------------------------------------------
----------------------------------------------------------------------
----------------------------------------------------------------------
-----------------------------------------MADTPTSRMV-HPFGDAPRQTPKQFLYSG
NPQHLCHP---------YQSAPDTHVVLQRRYTVRSQ--SHSPNNAGSEDHETH----------------
----------KQYTLESSAASGCSRH--------GSPSSQSVHAG----------SGSPV--SHDDSHS-
GSTNGHGS--PVSASC--VTGEDPTDLKQKLKDLE-AVMLGTSETDPEIVNSLEISAA--NQLS------
----------------------------------------------------------------------
----------------------------------------------------------------------
----LEPEEWE---------------------HMVSMPRGNLK-ELLIACARAVERNNSYAIDLMITELR
KMVSVSGEP-LERLGAYMVEGLVARLAASGS-------SIYKALK---------CKEPRSSD--LLSYMH
F-LYE---------------ACPYFKFGYMSANGAIAEAIKGEDRIHIIDFHIA-QG--A----QWVSLL
QALA-ARP----GGP----P-FVRVTGIDDSVSAYARGGGLELVGRRLTHIAGLYKVPFQFD-AVA-ISG
SEV-EE------EHL----------GVV-------PGEAVAVNFTLELHHIPDETVSTANHRDRILRLVK
G-LSPKVLTLVEQESN--TN--------------TAPFAQRFAETLDYYTAIFESIDLA-----------
-----LP------RDD-RERINIEQHCLAREIVNLVACEGEERV-ER-HEVF-GKWKARLMMAGFSPSPL
SALV-NATIKTLL--QSY--------SP--DYKLA---ER--DGVLYLGWKNRPLIVSSAWH--------
--------------------------
>AT1G14920.1
----------------------------------------------------------------------
----------------------------------------------------------------------
```

```
---MKRDHHHH---------------HHQDKKTMMMNEED-----DGNGM------DELL----------
----------------------------AVLGYKVRSSEMADVAQKLEQLEVMMSNVQ------------
----------------------------------------------------------------------
--------------------------------------------------------------------ED
DLSQLATETV-------HYNPAELYTWLDSMLTDLNPP--------------------------------
----------------------------------------------------------------------
----------------------------------------------------------------------
---SSNAEYDLKAIPGDA-ILNQFAIDSASSSNQGGGGDTYTTNKRLKCSNG------------------
------VVETTTATAE------STR---------------------------------------------
--------HVVL--------------------VDSQENGVRLV-HALLACAEAVQKENLTVAEALVKQIG
FLAVSQIGA-MRKVATYFAEALARRIYRLSPSQS-----------------------PIDHS--LSDTLQ
MHFYE---------------TCPYLKFAHFTANQAILEAFQGKKRVHVIDFSMS-QG--L----QWPALM
QALA-LRP----GGP----P-VFRLTGIGPPAP--DNFDYLHEVGCKLAHLAEAIHVEFEYRGFVA-NTL
ADL-DA------SML----------ELR-----PSEIESVAVNSVFELHKLL-GRPGA---IDKVLGVVN
Q-IKPEIFTVVEQESN--HN--------------SPIFLDRFTESLHYYSTLFDSLEGV-----------
-------------PSG-QDKV-MSEVYLGKQICNVVACDGPDRV-ER-HETL-SQWRNRFGSAGFAAAHI
GSNA-FKQASMLL--ALFNG------GE--GYRVE---ES--DGCLMLGWHTRPLIATSAWKLSTN----
--------------------------
>Pp3c7_1530V3.1.p
----------------------------------------------------------------------
----------------------------------------------------------------------
----------------------------MAATHVVSKKRS-----PTIFSEVATATCKRW----------
----------------------------KSEAAWHHVEEMAPEPPPIQDFDLKVKTSCLK----------
------------------------------------------------------MVQ-------------
-----PEPTSVLDLQ-------------ASPGRSCSSSTSLSSGT-----------------DSPHSIST
DSPNFSTTVQ-------QVEPVDIASWVDCMALPEFEDCDRHLEEVLKSDIDFA----------------
--STDLDFGFELTSLDHCSVVTEHGYSVSLLSEFLGDP--------------------------------
------------------------------------DENLLLPESFHDAKRLQELDDS--LSSMLSEVRS
SGSDSGSSV-------------------------------------------------------------
-------PTTVELARL------VESLPCSD-L---------------------RRHGGVDTKHHHHHSRS
ESWGSTSKLQTL--------------------QHPEDSGLQLV-HMLLACAEAIEKSDFNKAKPILDQLL
RSSDPYGDP-MQRIALYFGEALTDHLA-----------GVVSP-----------SETHLLSD--SKLAYQ
A-FYK---------------VLPFAKFSHVTANQTIYEAVVRSQNVHVVDLDIQ-LG--L----QWPCFI
QSLA-MRP----GGA----P-HLRISAIGTNAE------NLQTTKRRLSEFAEALKVPFEFT-PVL-SSL
ENL-TA------AML----------DIR-------SEEDLAINCSQVLHTLS-GEEAV---LDKLLSMFH
N-LKPNVVTLLEAEAN--HN--------------GASFIARFVEALHYYCALFDSLEGA-----------
-----LG------RDS-ADRYHIESTALAAEIKEIVAFKGNRRR-VR-HVRS-ETWRGLFAKAGFLSMAF
SSYT-VQQAQMLL--EVLTSKPMQQANATMPYKLS---QE--STSLILGWQETPVIGVSAWTC-------
--------------------------
>GRMZM2G098517_P02
----------------------------------------------------------------------
----------------------------------------------------------------------
----------------------------------------------------------------------
----------------------------------------------------------------------
----------------------------------------------------------------------
-----------------------------------------MADPPTSRMVQHPFGDIPSQTPKQFLYSG
SSQHLCHP---------YQSASDAHVAPQRHYTVRSQSQSQSP-DAGSEDFETH----------------
---------SRQYTLDSSSASGCSGH--------GSPSCQSVHAG----------SRSPVSHSHDDSHS-
GSTNGNGS--PASASC--VT-EDPTDLKQLKLDLE-AVMLGT-DTDPETVDSLEIAIA--DRLS------
----------------------------------------------------------------------
----------------------------------------------------------------------
----VEPEEWKN--------------------NMVSVPRGDLK-ELLIACARAVEQNNGYSIDLMVPELR
```

```
KMVSVSGEP-LERLGAYMVEGLVARLAASGS-------SIYKALR---------CKEPRSSD--LLSYMH
F-LYE---------------ACPYFKFGYMSANGAIAEAVKGEDRIHIIDFHIA-QG--A----QWVSLL
QALA-ARP----GGP----P-FVRVTGIDDPVSAYARGGGLELVGKRLSHIAGLYKVPFQFD-AVA-ISG
SEV-EE------GHL----------GVV-------PGEAVAVNFTLELHHIPDETVSTANHRDRVLRLVK
G-LSPRVLTLVEQESN--TN--------------TAPFAQRFAETLDYYAAIFESIDLA-----------
-----LP------RGD-RERINIEQHCLAREIVNLVACEGEERV-ER-HEVF-GKWKARLMMAGFRPSPL
SALV-NATIKTLL--QSY--------SP--DYKLA---ER--EGVLYLGWKNRPLIVSSAWH--------
--------------------------
>Aco010118.1
----------------------------------------------------------------------
----------------------------------------------------------------------
----------------------------------------------------------------------
----------------------------------------------------------------------
----------------------------------------------------------------------
-------------------------MTEQGFEADCALGIIQSNDQWLCNQIGFALPPDNMQMTAPPYDRS
LLDGHYTATV-------TATATDLSEFMDQLIRPNPSLS-------------------------------
----------------------------------------------------------------------
----------------------------------------------------------------------
----------------------------------------------------------------------
-------QNTDDFSID------NTPSNN-------------------------NYVAQSDVHTPPQKAPR
RDCYERSGDEGG----------------E---RPHHDHEMRMI-TLLMECAVQISTENLGEAQNMLLELT
QMASPYAASCAERVVAYFAEAMASRIVNSCI-------GVCSPL------------LLPYKT--ILSAFQ
A-FNN---------------LSPFIKFGHFTANQTILEAFHQEERIHIIDLDIM-QG--L----QWPALF
HILA-TRP----NGP----P-QVRMTGLGPSGP------VLEETGMQLAGFARRLGMRFEFH-PIA-RRS
GDL-DP------SSVIIPP------GRR-------SGEAVAVHLMR--HALY-DATGD---ESNTMRLLE
H-LGPRVVTLVEQEMG--HN--------------CGPFLGRFVGALHYFSTVFDSLGSS-----------
-----WA------VDD-VGRHRVESGLLAREIANVVAVGGPGR--GG-EEKV-GRWRDELARRGFVQVEM
SGNA-MAQAQLIV--NMFPP------AL--GYSVLH--GS--DGSLGLGWKGAGLYTASAWTARSSL---
--------------------------
>Pp3c2_18410V3.1.p
----------------------------------------------------------------------
----------------------------------------------MAEITHYRPHSDTNTVKFATVPDL
QTYLGISSWLELNQHWRLGDATKFFKVAEHEGQNSMSSAV-----DSKRLDSGKSGNQHS----------
----------------------------TALAMSKEPTSVLDLPSSCSVSLSSQHSMNSVSDGISKA---
----------------------------------------------------------------------
--------------------------------------------------LQTVADQLLSDEQDSAHPMQ
SATPETSNML-------MDQ--DVMTWMSLTRGSDHSASDMDPVPDWFHKQPER-LDLNAQQYYCSKTGL
DFHKNPGTMERICNNLPSSHHHYVAQPFKLISQAQLTMSETAGNQQTGFFD-------------------
------------------------------------TSTSSNQRAFQQCRNVKAQGAT--LMSMIDGTGA
ITGSQLLNSTVVPTPELWKLLSSQ-----------------HDASPQCGNQG------------------
-------PPNKQAPIT------QARDQQNR-S---------------------TSLAIPTQQPQPQLPYH
FGQTLYPGVT----------------------PQDQETGIHMV-HLLLACAEAVDMCQSATAGPMLARLR
SIYDPEGEP-MRRIALYFAEALFERLTIEMNRKQSSHHGSCVRFPEPEVD----SAASPSLE--CDIAYQ
A-YYQ---------------ILPFKKFTHLTANQALLEGVANYPRVHIIDFNIR-QG--L----QWPSFI
QSLA-MLP----RGP----P-QLKFTAVQTDAA------TVQKTGNRLAEFARTMHVPFEFY-ILE-ESV
ESF-HQ------GMI----------SPR-------AEEALAVNCSDMLHRLL-RKEGK---LTELLGKIR
S-LQPVVVTVLEVDAN--HN--------------EPSFMPRFVHALHYYCAVFDSLEAA-----------
-----LL------RNS-LDRLRIENHCFSTQIRSIIALEDVDRE-IR-HVRA-ETWQSHFLQAGFRAVTV
SRYA-ADQAQLLL--GLYKP------SDRMPFTLS---SG--FGGLSLGWRETPVVAVSSWTFSPPPY--
--------------------------
>Solyc04g064550.1.1
----------------------------------------------------------------------
```

Development • Supplementary information

```
----------------------------------------------------------------------
----------------------------------------------------------------------
----------------------------------------------------------------------
----------------------------------------------------------------------
----------------------------------------------------------------------
MSLVRSLRSIGNGKLYFQNGHNDNSSLATSMYTKNARGIMYATESSST----------------------
------DSYDPKYLLESPSPSEELLN--------TSPTDVLGNPFHQRHSSSFHPSRDY---NQVSYDSA
DCV-----NQSPDSSE-----YNDGRVTMKLQELE-RVLFDDNEIEGDDVFARG------ETVDIDDEWF
NQIRTELL--------------------------------------------------------------
-------------------------------------------------------------QESPKESTS
ADSNTSSSSSYK--------------------EISVSAPQTPK-QMLFSCAAAIQDGHIEQASSMINELR
QMVSIQGDP-LGRTAAYMVEALAARMATSGR-------GLYKALK---------CKEATSSE--RLSAMQ
V-LFE---------------VCPYFRFGFMAANGAILEAFKDEKRVHIIDFDVN-QG--S----QYYTLL
QTLG-SMP----GKP----P-HVRLTGVDDPESVQRAIGGLNVIGLRLAQLAKDLKISFEFQ-AVS-SNT
ALV-TP------AML----------NCR-------PGEAVLVNFAFQLHHMPDESVSTVNQRDQLLRMVK
S-LNPKLVTVVEQDMN--TN--------------TAPFLQRFAEVYNYYCAVFESLDAT-----------
-----LS------RDS-QERVNVERQCLARDIINIVACEGLERI-ER-YEVA-GKWRARMMMAGFTPSPI
SRNV-YESIRNLI--KQY--------SE--RYKAE---EE--AGALYFGWEDKTLTVASAWR--------
--------------------------
>Sevir.2G380500.1.p
----------------------------------------------------------------------
----------------------------------------------------------------------
----------------------------------------------------------------------
----------------------------------------------------------------------
----------------------------------------------------------------------
-----------------------------------------MADTPTSRMV-HPFGNVPRQTPKQFLYSG
NTQHLCHP---------YQSASDTHVVPQHHYTMK----SHSP-DAVSEEHETR----------------
----------KQYTLDSSAASGCSRH--------DSPSSQSIHTG----------SGSPL--SHEDSHS-
GSTNGNGS--PVSASC--VT-EDPTDLKQKLKDLE-AVMLG---TDSEIVDSLEISVA--NQLS------
----------------------------------------------------------------------
----------------------------------------------------------------------
----LEPEKWV---------------------HMMSMPKGNLK-ELLIACARAVEQNNSFAIDLMIPELR
KMVSVSGEP-LERLGAYMVEGLVARLASSGN-------SIYKALK---------CKEPKSSD--LLSYMH
F-LYE---------------ACPYFKFGYMSANGAIAEAVKGEDRIHIIDFHIA-QG--A----QWISLL
QALA-ARP----GGP----P-FVRITGIDDSVSAYARGGGLELVGRRLSHIAGLYKVPFQFN-AVA-ISG
NEM-EE------GHL----------GIV-------PGEAVAVNFTLELHHIPDETVSTANHRDRILRLVK
S-LSPKVLTLVEQESN--TN--------------TAPFAQRFAETLDYYTAIFESIDLA-----------
-----LP------RED-RERINMEQHCLAREIVNLVACEGEERV-ER-HEVF-GKWKARLMMAGFRPSPL
SALV-NATIKTLL--QSY--------SP--DYKLA---ER--DGVLYLGWKNRPLIVSSAWH--------
--------------------------
>Aco002115.1
----------------------------------------------------------------------
----------------------------------------------------------------------
---------------------------MLQSLLPRSPITNAASSDAPPMSRPKRSADARD----------
----------------------------------------------------------------------
----------------------------------------------------------------------
----------------------------------------------------------------------
----------------------------------------------------------------------
----------------------------------------------------------------------
----------------------------------------------------------------------
----------------------------------------------------------------------
----------LTPSSS------SPAAAAAACDDDDD-----------------RLRKRKQLQLYPQPEPQ
```

Development • Supplementary information

```
PPQQQQQQHVVDVIQERKNDNTYFSAGSSSSSSAVESRVLRLL-GLLLHCAEAVAADRLSEARDILPEIN
ELASPFGSS-PERVAAYFADALGARILSSYL-------GVYSPLIHHHKPL----VTSHHNR--IFNAFQ
V-YNS---------------ISPLVKFSHFTSNQAIFQALDGEERVHIIDLDIM-QG--L----QWPGLF
QILA-SRP----LKL----R-SLRITGLGSSLE------LLEATGRRLSDFAAALGLPFEFQ-PVE-GKI
GDQSKAGGGG--GAF-A--------PRD-------GREVAVVHWMH--HCLY-DVTGS---DEAAVSVIK
NVIRPRLVTVVEQDLG-RH---------------GGDFLGRFVEALHYYSAVFDALGEG-----------
-----AAAEGSREEEE-AERHDVERQLLGAEIRNIVAVGGPKR--TG-EVAV-GRWGEEMRRAGFEQVSL
AGGPAAAQANLLL--GMRLP------WK--GYTLV---EE--AGCLKLGWKDLSLLTASAWKTGNSHGPA
EGGEDRDHVQPPPQQQQHHHHSSLLT
>Sobic.002G330300.1.p
----------------------------------------------------------------------
----------------------------------------------------------------------
----------------------------------------------------------------------
----------------------------------------------------------------------
----------------------------------------------------------------------
----------------------------------------------------------------------
---------------------MDLHQLLKYRLTGANVFYEFPTENNLANTPWPA----------------
--------SPLKSEFSGSPYTPLSSQLECDNLSALSNTPDNQ-SSTETISAQ---PISPLEVDSSYRQAA
I-LRENTQVRPDPLYA-----TSRHNMQHALREIE-TVLMA---PDAEDAATSTKHEFEEHKPAQLMRQR
SRTWSHESRQ------------------------------------------------------------
-----------------------------------------------------PSPGVVRTQFAS-GYPT
ASYEFRPEKRLR--------------------ELREDPQSMVK-QLLTKCAEALSEERIEEFLTLVQQAR
GVVSITGEP-IQRLGAYLLEGLVARHANSGT-------NIYRALK---------CREPESNE--LLSYMK
I-LYN---------------ICPYFKFGYMAANGAIAEALRNEDKIHIIDFQIA-QG--T----QWITLI
QALA-AKP----GGP----P-HVRITGIDDPVSEYARGEGLDLVGKMLKSMSEEFRIPLEFT-PLPGIYA
TQV-TK------EML----------DIR-------PGEALAVNFTLQLHHTPDESVDVSNPRDGLLRMVK
G-LSPKVTTLVEQESH--TN--------------TTPFLMRFTETLDYYSAMFESIDAN-----------
-----LP------REN-KERINVEQHCLAKDIVNIIACEGKDRV-ER-HELL-GKWRSRLTMAGFRPYPL
SSYV-NSVIRNLL--AYY--------SD--KYTLE---EK--DGAMLLGWKNRKLISASAWH--------
--------------------------
>Aco013815.1
----------------------------------------------------------------------
----------------------------------------------------------------------
----------------------------------------------------------------------
--------------------------------------------MQIAKTPINSGIEVSIAQKLARL---
----------------------------------------------------------------------
----------------------------------------------------------------------
----------------------------------------------------------------------
----------------------------------------------------------------------
-EEIFLVP-------------------------------GASCFPL------------------------
----------------------------------------------------------------------
-------SVNFFKPDE------GFDRYEG------------------------------------------
--------------------------------LILEQPDAELV-SLLVSCAESISSLNHEAVDYFLARLG
ERASPRGTT-VQRVAAYFTEAFAFRAVRLFP-------HMFSIAPPRELTYHIEEYSDDDDD--GATALR
L-FNY---------------VSPIPKFLHFTLNERLLQAFDGKERVHVIDFDIK-DG--L----QWPSLL
QSLA-SRP----NPP----S-HVRITGIGKYRE------NLQDAGVRLARFAEALNLPFKFH-AVV-ERL
DDV-RL------WML----------HVK-------RDECIAVNCVLVMHKLLSDVSGSA--LRRFLELIR
S-TNPSIVLLGEQEDE--NN--------------EQRWEVRFGKSLQYYSAIFDSLDSL-----------
-----LP------ENS-ALRIKVEE-MFAREIRTIVAWEGSERV-ER-HESF-RRWREIMENGGFRNLDI
GERE-MAQSQTIV--RMYS-------SE--NFTVERQGEGEGYRGLTLKWLDQPLYTVSAWAPNEPAHVG
YSSLSLPT------------------
>LOC_Os05g40710.1
```

```
----------------------------------------------------------------------
----------------------------------------------------------------------
----------------------------------------------------------------------
----------------------------------------------------------------------
----------------------------------------------------------------------
----------------------------------------------------------------------
----------------------------------------------------------------------
----------------------------------------------------------------------
----------------------------------------------------------------------
------------MQDSLGLM-QFLD--HHQYLYSSSSSNLPL-QQPLLSHHHRFLEAN----------EG
CAGEDDSPEFVEPPAA------AAAAGTFE-QR--------------------PELGACKEVYSEEGGAA
-----EERTGVAMAGA--------DVEQVAVEDEEEAHGVRMI-ALLMECAAAMSVGNLAGANGALLELS
QMASPYAASCGERLVAYFARAMAARLVGSWV-------GVVAPMA----------PPPSCGA--INAAFR
A-LYN---------------VAPFARLAYLACNQAILEAFHGKRLVHIVDLDVV-PGGAL----QWLSLL
PALA-ARP----GGP----P-VIRVTGFGMSAS------VLHDTGNQLAGLARKLCMFFEFY-AVA-KRP
GDA-DAVA----DMP----------GRR-------PGEAVAVHWLR--HAMY-DAAGD---DGASMRLVR
W-LEPAAVTLVEQERA--HGGGGG----------HGRFLDRFVSALHHYSAVFDAMGAS-----------
-----RPD-----GED-ASRHLAEHGVLGREIANVLAVGGPARS-SG-REGP-GSWREVLARHGFAHAGG
GG---GGRAQLVA--AACPG------GL--GYTVA---GD-HDGTVRLGWKGTPLYAVSAWTWCSPPHAR
A-------------------------
```

Development • Supplementary information
